# Neurodevelopmental Pathways from Maternal Obesity to Offspring Outcomes: An Umbrella Review of Cognitive and Behavioral Consequences Across Development

**DOI:** 10.3390/healthcare13202653

**Published:** 2025-10-21

**Authors:** Evgenia Gkintoni, Eleni Papachatzi, Erifili Efthymiadou, Emmanuella Magriplis, Apostolos Vantarakis

**Affiliations:** 1 Laboratory of Public Health, Department of Medicine, University of Patras, 26504 Patras, Greece; elepapach@upatras.gr (E.P.); emagriplis@aua.gr (E.M.); avanta@upatras.gr (A.V.); 2 Department of Psychiatry, University General Hospital of Patras, 26504 Patras, Greece; 3 Department of Pediatrics, University General Hospital of Patras, 26504 Patras, Greece; 4 Lucy Faithfull Foundation, Epsom KT17 1HQ, UK; eefthymiadou@lucyfaithfull.org.uk; 5 Laboratory of Dietetics and Quality of Life, Department of Food Science and Human Nutrition, Agricultural University of Athens, 26504 Athens, Greece

**Keywords:** maternal obesity, neurodevelopment, cognitive development, executive function, ADHD, epigenetics, inflammatory pathways, developmental programming, umbrella review, behavioral outcomes

## Abstract

**Background**: Maternal obesity affects 20–25% of pregnancies globally and has been associated with adverse offspring neurodevelopmental outcomes. This umbrella review synthesized evidence on neurodevelopmental pathways linking maternal obesity to offspring cognitive, executive, and behavioral outcomes. **Methods**: Following PRISMA 2020 guidelines, we systematically searched six databases (PubMed/MEDLINE, Scopus, Web of Science, PsycINFO, EMBASE, CINAHL) for studies published 2008–2024. We included original peer-reviewed studies examining maternal pre-pregnancy obesity (BMI ≥ 30 kg/m^2^) and offspring neurodevelopmental outcomes using prospective cohort, experimental, neuroimaging, or systematic review designs with validated assessments. Risk of bias was assessed using Newcastle–Ottawa Scale, Cochrane RoB 2.0, and SYRCLE guidelines. **Results**: Analysis of 78 studies encompassing 650,000+ mother–child pairs from 17 countries revealed significant associations. Study designs included prospective cohorts (59%), animal experiments (22%), systematic reviews/meta-analyses (13%), neuroimaging studies (4%), and randomized trials (3%). Maternal obesity (BMI ≥ 30 kg/m^2^) was associated with reduced cognitive abilities (IQ differences: −2.5 to −5.8 points), impaired executive function (OR 1.4–2.3), and increased ADHD symptoms (OR 1.4–2.8) and emotional dysregulation (OR 1.5–2.2). Dose–response relationships revealed threshold effects at BMI ≥ 30 kg/m^2^, accelerating at BMI ≥ 35 kg/m^2^. Four primary mechanistic pathways were identified: inflammatory, metabolic, epigenetic, and neurotransmitter alterations. Only 57.7% of studies used prospectively measured pre-pregnancy BMI. **Conclusions**: Observational and experimental evidence indicates maternal obesity represents a modifiable risk factor for offspring neurodevelopmental impairment. The primarily observational human evidence, supported by mechanistic animal studies, suggests multimodal interventions targeting identified pathways during critical windows (pre-conception through early postnatal period) warrant investigation.

## 1. Introduction

Maternal obesity has emerged as one of the most pressing public health challenges of the 21st century, affecting approximately 20–25% of pregnant women globally according to recent WHO data (2023) and reaching epidemic proportions in developed countries with prevalence exceeding 30% in the United States and several European nations [1,2]. This metabolic condition represents a complex, multifactorial disorder characterized by excessive accumulation of adipose tissue, which fundamentally alters the intrauterine environment during critical periods of fetal neurodevelopment [3]. The growing prevalence of maternal obesity coincides with concerning trends in childhood neurodevelopmental disorders, cognitive impairments, and behavioral problems, raising essential questions about potential causal relationships and underlying biological mechanisms [4,5].

The developing fetal brain seems to respond to maternal metabolic perturbations throughout gestation, with emerging evidence suggesting that maternal obesity creates a suboptimal intrauterine environment that can influence offspring neurodevelopment [6,7,8,9]. Unlike genetic factors, maternal obesity represents a potentially modifiable risk factor that could significantly impact population-level neurodevelopmental outcomes. Early identification of at-risk offspring and an understanding of the underlying mechanistic pathways are crucial for developing targeted interventions and prevention strategies that can improve long-term cognitive, executive, and behavioral outcomes across the lifespan [10,11,12].

Recent advances in neuroimaging technologies, biomarker identification, and longitudinal study methodologies have provided insights into the complex neurodevelopmental pathways linking maternal obesity to offspring outcomes [13,14,15,16]. These, combined with animal models and multi-omics approaches, enable researchers to investigate mechanistic relationships spanning prenatal exposures through childhood and adolescent development. The integration of epidemiological evidence with mechanistic studies offers the potential to develop evidence-based interventions that could mitigate the neurodevelopmental consequences of maternal obesity [17,18,19,20].

The implications of maternal obesity encompass significant societal and economic consequences. Children experiencing neurodevelopmental impairments may require increased educational support, specialized interventions, and long-term healthcare services. The economic implications are substantial, with estimates suggesting that maternal obesity-related neurodevelopmental impairments may cost healthcare and education systems between $3–8 billion annually in developed countries, considering increased special education needs, behavioral intervention services, and long-term healthcare utilization [21,22,23]. Understanding the pathways linking maternal obesity to offspring neurodevelopmental outcomes is therefore crucial not only for individual families but also for public health planning and resource allocation [24,25,26].

Current research in this field encompasses a diverse range of methodological approaches, from large-scale epidemiological cohort studies to controlled experimental investigations in animal models [27,28,29], offering complementary perspectives for researchers to identify consistent patterns across various study designs and populations. The convergence of evidence from multiple research paradigms strengthens our understanding of these critical neurodevelopmental pathways [30,31,32,33,34].

This review aims to comprehensively synthesize current evidence regarding the neurodevelopmental pathways linking maternal obesity to offspring cognitive, executive, and behavioral outcomes across development. Specifically, it aims to systematically analyze how maternal pre-pregnancy and gestational obesity influence offspring neurodevelopmental trajectories from the prenatal period through childhood and adolescence, examining the underlying biological mechanisms and identifying critical exposure windows that may inform prevention and intervention strategies.

## 2. Contemporary State of the Art

### 2.1. Understanding Maternal Obesity and Neurodevelopmental Programming

Maternal obesity, typically defined as pre-pregnancy body mass index (BMI) ≥ 30 kg/m^2^, represents a complex metabolic condition that fundamentally alters the intrauterine environment during critical periods of fetal brain development. Current prevalence estimates indicate that maternal obesity affects 20–35% of pregnant women in developed countries, with rates continuing to rise globally. The condition involves multiple pathophysiological alterations, including chronic low-grade inflammation, insulin resistance, altered hormone profiles, and modified nutrient availability, all of which can influence fetal neurodevelopmental processes [35,36,37,38,39].

The Developmental Origins of Health and Disease (DOHaD) provides the theoretical framework for understanding how maternal obesity influences offspring neurodevelopment. This paradigm proposes that environmental exposures during critical developmental windows can permanently alter the structure and function of organs and metabolic systems, with lasting consequences for health throughout the lifespan [13,14,15]. This framework provides a theoretical foundation for understanding how maternal obesity during pregnancy might program offspring neurodevelopmental trajectories through biological mechanisms operating across sensitive periods of brain development. The developing brain is vulnerable to maternal metabolic perturbations due to its rapid growth, high energy demands, and extended developmental timeline, spanning from early gestation through adolescence [40,41].

Maternal Metabolic Alterations in Obesity: Maternal obesity is characterized by multiple metabolic perturbations that can influence fetal neurodevelopment. Chronic inflammation, marked by elevated circulating levels of pro-inflammatory cytokines including tumor necrosis factor-alpha (TNF-α), interleukin-6 (IL-6), and C-reactive protein (CRP), creates a pro-inflammatory intrauterine environment. Insulin resistance and hyperglycemia alter glucose availability and utilization in the developing brain leading to fetal hyperinsulinemia, particularly concerning because insulin acts as an anabolic hormone that can potentially impair brain development through its growth-promoting effects and metabolic programming. Dysregulated hormone profiles, including elevated leptin levels, reduced adiponectin levels, and altered cortisol patterns, can further influence neurodevelopmental processes. Additionally, altered lipid metabolism affects the availability of essential fatty acids, which are crucial for brain development [42,43,44].

Critical Developmental Windows: Neurobiological evidence indicates that specific gestational periods may represent critical windows of vulnerability to the effects of maternal obesity. Early pregnancy (first trimester) coincides with fundamental processes of neural tube formation, neurogenesis, and early brain regionalization [45,46,47]. The mid-pregnancy period (second trimester) encompasses critical periods of neuronal migration, synaptogenesis, and early circuit formation [48,49]. Late pregnancy (third trimester) involves ongoing synaptogenesis, myelination initiation, and the establishment of basic neural networks. Each developmental period may demonstrate differential sensitivity to maternal metabolic perturbations [50,51,52].

Mechanisms of Neurodevelopmental Programming: Multiple biological mechanisms have been implicated in maternal obesity-related neurodevelopmental programming. Inflammatory signaling pathways, including those mediated by nuclear factor-kappa B (NF-κB) and Toll-like receptor (TLR) signaling, can directly influence neuronal development and survival. Epigenetic modifications, including DNA methylation, histone modifications, and changes in microRNA expression, provide mechanisms for long-lasting alterations in gene expression patterns. Altered growth factor signaling, including brain-derived neurotrophic factor (BDNF) and insulin-like growth factor (IGF), can influence neuronal proliferation, differentiation, and survival. Oxidative stress and mitochondrial dysfunction may impair cellular energy metabolism in developing neurons [53,54].

### 2.2. Cognitive and Executive Function Outcomes

Cognitive and executive function deficits represent prominent neurodevelopmental consequences associated with maternal obesity exposure [55,56]. These impairments manifest across multiple cognitive domains and demonstrate persistence throughout childhood and adolescence, suggesting fundamental alterations in brain development and function [57,58,59].

General Cognitive Abilities: Meta-analytic evidence consistently demonstrates modest but significant associations between maternal obesity and reduced general cognitive abilities in offspring. Intelligence quotient (IQ) assessments reveal mean differences of approximately 2–5 points between children of obese mothers and those of mothers with normal weight, with effects observed across verbal, performance, and full-scale IQ measures. These differences, while modest at the individual level, represent substantial population-level impacts given the high prevalence of maternal obesity. Longitudinal studies suggest that these cognitive differences emerge early in development and persist throughout childhood [60,61,62].

Language and Verbal Abilities: Maternal obesity demonstrates particularly robust associations with language and verbal development impairments. Vocabulary acquisition, as assessed by standardized psychometric tests such as the Peabody Picture Vocabulary Test (PPVT), shows consistent delays in children exposed to maternal obesity. Expressive and receptive language abilities, measured through comprehensive language assessments, demonstrate reduced performance across multiple linguistic domains. Language-based academic abilities, including reading comprehension and verbal reasoning, show persistent impairments that may contribute to educational underachievement [63,64,65,66,67].

Executive Function Deficits: Executive function impairments represent a particularly concerning consequence of maternal obesity exposure, given their fundamental role in academic achievement, social functioning, and behavioral regulation. Working memory capacity demonstrates consistent deficits in children exposed to maternal obesity [68,69]. Inhibitory control, measured through tasks requiring response suppression or interference resolution, shows impaired performance across multiple paradigms. Cognitive flexibility, a fundamental for executive function, is shown to have reduced efficiency and accuracy. Attention and sustained attention processes show increased distractibility and reduced sustained performance [70,71,72].

Neural Correlates of Cognitive Impairments: Neuroimaging studies have identified specific brain alterations that may underlie cognitive and executive function deficits associated with maternal obesity. Structural magnetic resonance imaging (MRI) reveals altered brain volumes in regions critical for cognitive function, including reduced hippocampal volumes associated with memory impairments and altered prefrontal cortex volumes linked to deficits in executive function. Diffusion Tensor Imaging (DTI) demonstrates white matter microstructural abnormalities in pathways connecting cognitive brain regions, potentially reflecting altered neural connectivity. Functional MRI studies reveal altered activation patterns during cognitive tasks, suggesting inefficient neural processing [73]. Advanced neuroimaging approaches, including EEG-based cognitive biomarkers, offer additional insights into the functional consequences of maternal obesity on offspring brain development and may serve as early detection tools for neurodevelopmental risk [74].

Developmental Trajectories: Longitudinal studies examining cognitive development trajectories reveal that the effects of maternal obesity may vary across developmental periods. Early childhood assessments often reveal the most pronounced effects, with some evidence for partial recovery during later childhood. Adolescent assessments suggest that some cognitive impairments persist, particularly in domains that require complex executive processing [75,76].

### 2.3. Behavioral and Emotional Outcomes

Behavioral and emotional problems represent significant neurodevelopmental consequences of maternal obesity exposure, with implications for social functioning, academic achievement, and long-term mental health outcomes. These behavioral alterations encompass multiple domains, including attention and hyperactivity problems, internalizing difficulties, externalizing behaviors, and challenges in social–emotional regulation [77,78].

Attention Deficit Hyperactivity Disorder (ADHD) Symptoms: Maternal obesity demonstrates robust associations with increased ADHD symptoms in offspring, potentially reflecting alterations in dopaminergic and noradrenergic neurotransmitter systems. Sustained attention deficits and increased distractibility demonstrate consistent associations across multiple studies. These symptoms often emerge during preschool years, suggesting fundamental alterations in attention regulation systems [79,80,81].

Internalizing Problems: Internalizing behaviors, including anxiety, depression, and withdrawal, show significant associations with maternal obesity exposure. Anxiety symptoms, show increased prevalence in children exposed to maternal obesity [82,83,84,85]. Depressive symptoms may emerge more prominently during adolescence as well as social withdrawal may contribute to peer relationship difficulties [86,87,88].

Externalizing Behaviors: Externalizing behaviors, including aggression, oppositional behavior, and conduct problems, appear linked with maternal obesity exposure. These behaviors often contribute to academic and social difficulties, creating cascading effects on development [89,90,91].

Social–emotional Regulation: Emotion recognition abilities, crucial for appropriate social responding, are impaired in exposed children [92,93,94,95] showing increased emotional intensity and reduced regulatory capacity. Social skills suffer deficits that may contribute to peer relationship difficulties [96,97,98,99].

Neural Mechanisms Underlying Behavioral Problems: Altered development of limbic system structures may contribute to difficulties in emotional regulation [100,101,102,103,104]. Disrupted prefrontal-limbic connectivity may impair behavioral control. Alterations in the neurotransmitters, including those affecting the dopaminergic, serotonergic, and GABAergic systems, may contribute to attention, mood, and behavioral regulation issues [105,106,107,108].

### 2.4. Biological Mechanisms and Pathways

Understanding the biological mechanisms linking maternal obesity to offspring neurodevelopmental outcomes is crucial for identifying potential intervention targets and developing evidence-based prevention strategies. Current research has identified multiple interconnected pathways through which maternal obesity may influence fetal and postnatal brain development [6,20,40,43,47,53].

Inflammatory Pathways: Chronic inflammation represents a central mechanism linking maternal obesity to offspring neurodevelopmental outcomes. Maternal obesity is characterized by elevated circulating levels of pro-inflammatory cytokines, including TNF-α, IL-6, IL-1β, and CRP, which do not directly cross the placental barrier but induce placental inflammation and dysfunction, affecting the fetal environment. These indirect inflammatory effects affect nuclear factor-kappa B (NF-κB) signaling pathways and influence neuronal survival, differentiation, and synaptic development. Toll-like receptor (TLR) signaling may mediate inflammatory effects on neural stem cell proliferation and differentiation [109,110].

Metabolic and Hormonal Pathways: Insulin resistance and hyperglycemia alter glucose availability and utilization in the developing brain, potentially affecting neuronal energy metabolism and survival. Dysregulated leptin signaling may influence hypothalamic development and regulation of energy homeostasis. Altered adiponectin levels may affect neuronal differentiation and synaptic plasticity. Cortisol and hypothalamic–pituitary–adrenal (HPA) axis dysregulation may influence stress responsivity and neurodevelopmental programming. Growth hormone and insulin-like growth factor (IGF) alterations may affect neuronal proliferation and brain growth patterns [111,112].

Epigenetic Mechanisms: These provide a crucial mechanism for long-lasting alterations in gene expression patterns associated with maternal obesity exposure. DNA methylation changes at specific gene promoters can alter the expression of genes crucial for neurodevelopment, including brain-derived neurotrophic factor (BDNF), as well as genes involved in synaptic plasticity and components of the neurotransmitter system. Histone modifications, including histone acetylation and methylation, can influence chromatin structure and gene accessibility. MicroRNA expression changes can post-transcriptionally regulate gene expression patterns important for neurodevelopment. These epigenetic modifications may persist throughout development and potentially into subsequent generations [113,114,115,116,117,118,119,120,121].

Oxidative Stress and Mitochondrial Function: Maternal obesity is associated with increased oxidative stress and mitochondrial dysfunction, which may significantly impact fetal neurodevelopment. Reactive oxygen species (ROS) production can damage developing neurons and interfere with normal developmental processes. Mitochondrial dysfunction may impair cellular energy metabolism, which is particularly problematic for the energy-demanding developing brain. Alterations in the antioxidant system may reduce the capacity to manage oxidative stress. These effects may be particularly pronounced in brain regions with high metabolic demands and ongoing developmental processes [122,123,124,125,126,127,128,129].

Neurotransmitter System Development: Maternal obesity may influence the development of major neurotransmitter systems crucial for cognitive and behavioral function. The dopaminergic system, crucial for attention, motivation, and executive function, may be altered through inflammatory and metabolic pathways. The serotonergic system, crucial for mood regulation and social behavior, appears sensitive to maternal metabolic factors. The balance of the GABAergic and glutamatergic systems, fundamental for neural network function, may be disrupted by maternal obesity-associated factors. These alterations in the neurotransmitter system may contribute to the cognitive, executive, and behavioral problems observed in exposed offspring [15,16,20,43,53,130].

### 2.5. Developmental Timing and Critical Periods

The timing of maternal obesity exposure during development appears to influence the pattern and severity of offspring neurodevelopmental outcomes. Understanding these critical periods provides insights into when interventions might be most effective and helps explain the heterogeneity of outcomes observed across studies [10,22,55,131].

Preconceptional and Early Pregnancy Effects: Pre-pregnancy obesity and early pregnancy may influence fundamental neurodevelopmental processes during the first trimester. Neural tube formation and early brain regionalization occur during this period, potentially making these processes vulnerable to maternal metabolic perturbations. Oocyte quality and early embryonic development may be influenced by maternal metabolic status prior to conception. Placental development and vascularization, crucial for subsequent fetal nutrient and oxygen delivery, may be programmed during early pregnancy [132,133,134].

Mid-Pregnancy Neurodevelopmental Programming: The second trimester encompasses critical periods of neuronal migration, cortical layer formation, and early synaptogenesis where neuronal precursors migrate from germinal zones to their final cortical positions, a process that may be vulnerable to inflammatory and metabolic disruption affecting the foundation for neural networks [135,136,137].

Late Pregnancy and Perinatal Effects: The third trimester and perinatal period involve continued synaptogenesis, early myelination, and the establishment of basic neural circuits which rapid brain growth during this period may be susceptible to maternal metabolic factors. The hypothalamic–pituitary–adrenal axis development occurs primarily during late pregnancy, potentially influencing long-term stress responsivity. Birth weight and gestational age, influenced by maternal obesity, may target some neurodevelopmental effects [138,139,140,141,142,143].

Postnatal Programming Through Lactation: Maternal obesity is associated with delayed lactogenesis and reduced breastfeeding duration [144]. Exclusive breastfeeding for at least four months is associated with reduced prevalence of overweight and obesity in both mothers and their children 2–5 years post-delivery [145]. Breastfeeding may help mitigate obesity-related meta-inflammation and support healthy post-partum weight changes in mothers [146,147,148,149]. Additionally, maternal exercise during pregnancy and lactation can enhance these protective effects against childhood obesity [150] for which it should actively supported.

## 3. Materials and Methods

This research intends to examine multiple outcome domains, including general cognitive abilities (IQ, language development, and academic achievement), executive function capabilities (working memory, inhibitory control, cognitive flexibility, and attention regulation), behavioral and emotional functioning (ADHD symptoms, internalizing and externalizing behaviors, and social–emotional regulation). It investigates how different degrees of maternal weight status—from overweight to obesity to severe obesity—create dose–response relationships with offspring neurodevelopmental outcomes.

The review assesses the methodological quality of existing studies, including study design characteristics, sample demographics, exposure assessment methods, outcome measurement approaches, control of confounding variables, and generalizability across diverse populations. It specifically examines how different research paradigms—from large-scale epidemiological studies to controlled experimental investigations—converge complementary evidence regarding these neurodevelopmental pathways. Beyond mechanistic considerations, the research assesses the translational implications of current evidence, examining the potential for developing evidence-based prevention strategies, early identification approaches, and targeted interventions that could mitigate the neurodevelopmental consequences of maternal obesity. It explores how understanding these pathways might inform clinical practice, public health policy, and future research directions.

### 3.1. Research Questions

Despite substantial progress in understanding the relationships between maternal obesity and offspring neurodevelopmental outcomes, several critical research gaps remain that limit our ability to develop comprehensive prevention and intervention strategies. The research questions below address these gaps by focusing on mechanisms, timing, individual variation, and translational applications.

[RQ1] Neurodevelopmental Outcomes Across the Developmental Spectrum: What relation is identified between maternal pre-pregnancy obesity and offspring neurodevelopmental outcomes from the prenatal period through childhood and adolescence, and how this varies across different developmental stages?

[RQ2] Specific Cognitive, Executive, and Behavioral Domains: How does maternal obesity specifically affect offspring cognitive abilities, executive function, and behavioral outcomes, and what are the relative effect sizes and clinical significance of these associations across different functional domains?

[RQ3] Biological Mechanisms and Pathways: What are the underlying biological mechanisms and pathways through which maternal obesity influences offspring neurodevelopment, and how do inflammatory, metabolic, epigenetic, and neurotransmitter-related mechanisms interact to produce observed outcomes?

[RQ4] Dose–Response Relationships and Critical Exposure Windows: How do different degrees of maternal weight status (overweight vs. obesity vs. severe obesity) and timing of exposure affect the magnitude and pattern of offspring neurodevelopmental outcomes, and what are the critical windows of vulnerability?

**Figure 1:**
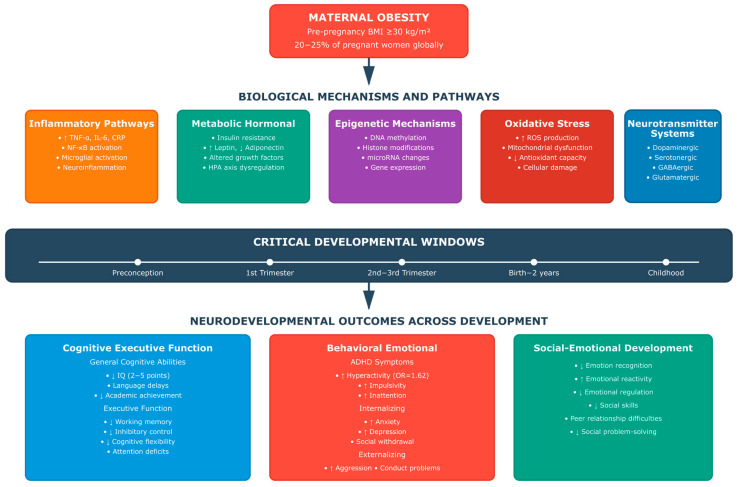
Conceptual Framework of Neurodevelopmental Pathways Linking Maternal Obesity to Offspring Outcomes.

The conceptual framework presented in Figure 1 synthesizes the complex relationships explored by these four research questions, providing a visual roadmap of the neurodevelopmental pathways from maternal obesity through biological mechanisms to offspring outcomes across critical developmental windows.

This framework not only guides our systematic analysis but also underlines the link of the mechanisms under investigation, emphasizing why a comprehensive, multi-domain approach is essential for understanding and ultimately preventing the neurodevelopmental consequences of maternal obesity.

### 3.2. Search Strategy

This review was conducted following the Preferred Reporting Items for Systematic Reviews and Meta-Analyses 2020 (PRISMA) guidelines [151], conveying methodological rigor and transparency to the collection and analysis processes. A protocol detailing the objectives, eligibility criteria, information sources, and analysis methods was registered on Open Science Framework (https://osf.io/76gyh (accessed on 10 September 2025)|Registration DOI: 10.17605/OSF.IO/76GYH) [152]. Academic databases PubMed/MEDLINE, Scopus, Web of Science, and PsycINFO were used for a systematic and comprehensive search across medical, neuroscience, psychology, epidemiology, and developmental biology field.

The search was limited to studies published from 2008 onward, as this period corresponds to several methodological advances that enhance evidence quality and comparability: (1) consistent global application of standardized BMI classification (≥30 kg/m^2^) for maternal obesity across research settings; (2) maturation of infant neuroimaging methodologies, particularly MRI protocols and resting-state functional connectivity analysis suitable for young children; (3) sufficient follow-up duration in major birth cohort studies (e.g., ALSPAC, Generation R, Norwegian Mother and Child Cohort) to assess school-age and adolescent neurodevelopmental outcomes; and (4) widespread availability of epigenetic research technologies, including genome-wide methylation arrays and next-generation sequencing, applicable to developmental programming research.

The search strategy employed a combination of vocabulary (MeSH terms) comprising four main areas: (1) maternal obesity and related conditions, (2) offspring neurodevelopmental outcomes, (3) biological mechanisms and pathways, and (4) developmental timing and critical periods. The search string database, was:

((“maternal obesity” OR “pre-pregnancy obesity” OR “prepregnancy obesity” OR “gestational obesity” OR “maternal overweight” OR “maternal BMI” OR “maternal body mass index” OR “obese mothers” OR “adiposity”) AND (“offspring” OR “children” OR “child” OR “infant” OR “fetal” OR “prenatal” OR “postnatal”) AND (“neurodevelopment” OR “cognitive development” OR “executive function” OR “ADHD” OR “attention deficit” OR “behavioral problems” OR “language development” OR “IQ” OR “intelligence” OR “memory” OR “learning” OR “social behavior” OR “emotional regulation”) AND (“pregnancy” OR “prenatal” OR “intrauterine” OR “fetal programming” OR “developmental origins” OR “DOHaD”)).

Additional specific searches were conducted for mechanistic studies using terms such as “inflammation,” “cytokines,” “epigenetics,” “methylation,” “biomarkers,” “metabolic programming,” “hypothalamic programming,” and “neurotransmitter development” combined with maternal obesity terms. The reference lists of identified articles, particularly recent systematic reviews and meta-analyses were manually screened to identify additional relevant studies. Additionally, forward citation tracking was performed for highly relevant papers to identify newer studies that had cited them. Two independent reviewers screened the titles and abstracts. The same reviewers assessed the full-text articles for eligibility, and a third reviewer resolved disagreements through discussion or arbitration.

### 3.3. Inclusion and Exclusion Criteria

Inclusion and exclusion criteria were established in accordance with the PRISMA guidelines to ensure a comprehensive and methodologically rigorous review. The criteria were designed to capture the most relevant studies addressing the neurodevelopmental pathways linking maternal obesity to offspring outcomes while maintaining the review’s focus on high-quality, peer-reviewed evidence.

#### 3.3.1. Inclusion Criteria

Our inclusion criteria encompassed original peer-reviewed studies published in English between 2008 and 2024 that specifically examined the relationship between maternal obesity, overweight, or elevated BMI and offspring neurodevelopmental outcomes. The year 2008 was selected as starting point to capture contemporary research utilizing modern neurodevelopmental assessment tools and standardized obesity definitions, while the 2024 endpoint ensured the most current evidence was included in this rapidly evolving field. We included research examining cognitive, executive, behavioral, or emotional development in offspring of obese or overweight mothers, as well as studies investigating biological mechanisms linking maternal obesity to offspring neurodevelopment, including inflammation, metabolism, epigenetics, and other pathways.

The review incorporated prospective cohort studies, animal models, neuroimaging investigations, and biomarker studies. Studies exploring developmental timing effects, critical periods, or dose–response relationships between maternal weight status and offspring outcomes were also considered. We focused primarily on pre-pregnancy obesity (BMI ≥ 30 kg/m^2^) as the key exposure, recognizing that pre-pregnancy metabolic status best reflects the periconceptional environment critical for early neurodevelopmental programming. However, studies assessing obesity in early pregnancy were also included when pre-pregnancy data were unavailable, as weight changes during early gestation are typically minimal.

#### 3.3.2. Exclusion Criteria

We excluded non-peer-reviewed articles including preprints, conference abstracts, editorials, or commentaries without original data to ensure methodological rigor as well as studies that did not directly address maternal obesity or focused solely on other maternal conditions such as diabetes or hypertension without obesity-specific analysis were excluded. Research examining only maternal weight gain during pregnancy without pre-pregnancy obesity assessment was excluded, as gestational weight gain represents a different physiological process compared to pre-existing maternal obesity.

Studies using only cross-sectional designs without developmental outcome assessment were excluded. This criterion specifically referred to studies that assessed both maternal obesity and offspring outcomes at a single time point without any longitudinal component or developmental trajectory assessment. However, retrospective studies that cross-sectionally assessed offspring outcomes but had reliable documentation of maternal pre-pregnancy obesity (through medical records or validated retrospective data collection) were considered eligible if they included appropriate developmental assessments and adequate control for confounding variables.

Other excluded were articles published in languages other than English with insufficient methodological rigor, inadequate sample sizes, inappropriate control groups, or lacking proper confounding variable control. While we did not apply a rigid sample size threshold given the heterogeneity of study designs, 28 studies were excluded for having sample sizes deemed insufficient for their specific research questions and analytical approaches.

Additional exclusions included publications focusing solely on maternal health outcomes without offspring neurodevelopmental assessment, duplicate publications or studies with substantially overlapping datasets with other included studies, as well as examining only birth outcomes such as birth weight or gestational age without subsequent neurodevelopmental follow-up. These criteria ensured our review maintained focus on the long-term neurodevelopmental consequences of maternal obesity rather than immediate perinatal outcomes, addressing the critical question of how maternal metabolic health programs offspring brain development and function across the developmental trajectory.

While gestational weight gain (GWG) is closely related to pre-pregnancy BMI and may mediate some effects of maternal obesity on offspring outcomes, we exclude studies examining only GWG without pre-pregnancy obesity classification. This decision was made to maintain clear exposure definition and avoid conflating pre-pregnancy metabolic status with pregnancy-related weight changes, which may reflect different biological mechanisms (e.g., chronic pre-existing inflammation vs. pregnancy-specific metabolic changes) and have distinct clinical implications. However, we acknowledge this exclusion may omit relevant mechanistic data, particularly regarding timing-specific effects during different trimesters of pregnancy. Studies that examined both pre-pregnancy obesity and GWG were included, with GWG effects considered as potential mediators (pathways through which pre-pregnancy BMI affects outcomes) or moderators (factors that modify the strength of BMI effects) were reported. Future reviews could specifically examine the independent and interactive effects of pre-pregnancy obesity and GWG on offspring neurodevelopment using mediation analysis frameworks.

### 3.4. Risk of Bias Assessment

Risk of bias was assessed using validated tools appropriate for each study design. For observational studies (*n* = 59), we applied the Newcastle–Ottawa Scale (NOS) evaluating selection, comparability, and outcome domains. For randomized controlled trials (*n* = 2), we used the Cochrane Risk of Bias 2.0 (RoB 2.0) tool. For animal studies (*n* = 17), we employed the SYRCLE Risk of Bias tool adapted for animal intervention studies, including assessment of randomization procedures, blinding, and nested data structure handling. Two independent reviewers conducted all risk of bias assessments, with disagreements resolved through discussion with a third reviewer. Each domain was rated as low, moderate, or high risk following published guidelines and decision rules specific to each tool. Overall risk of bias assessment revealed a generally high-quality evidence base. For observational studies, 42 (53.8%) showed low risk of bias across all domains, 25 (32.1%) showed moderate risk primarily due to partial confounder adjustment, and 11 (14.1%) showed high risk due to inadequate confounding control or reliance on self-reported exposure data. Most animal studies (14/17, 82%) demonstrated appropriate randomization and blinding procedures, with low risk of bias in key domains. Publication bias was assessed through examination of funnel plots where applicable in included meta-analyses. Among the 10 systematic reviews included, those conducting meta-analyses (*n* = 7) reported formal publication bias assessments using Egger’s test or visual funnel plot inspection, with most showing no significant evidence of publication bias (*p* > 0.10).

### 3.5. Analytical Search Process

**Figure 2:**
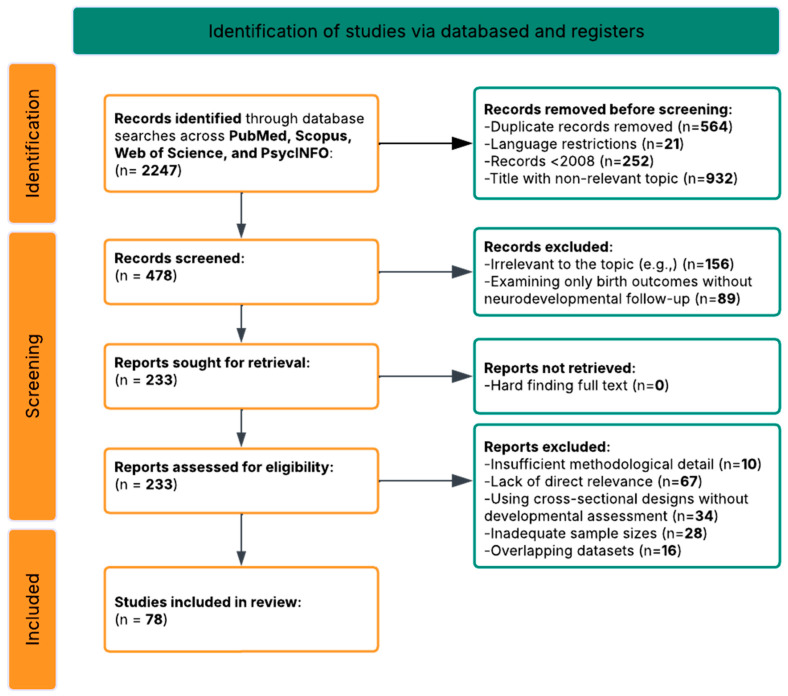
PRISMA flow diagram of the study selection process.

The search process began by identifying 2247 records through database searches across PubMed/MEDLINE, Scopus, Web of Science, and PsycINFO using the core search string and additional query variations tailored to specific research questions. After removing duplicates using automated tools and manual verification, 1683 unique records remained. These records were then screened by two independent reviewers using standardized screening forms based on title and abstract, and the whole selective process is described in the PRISMA flow diagram (Figure 2).

After this rigorous eligibility review, 78 articles met all inclusion criteria and were selected for qualitative synthesis (Figure 2) comprising 46 prospective cohort studies, 17 animal experimental studies, 10 systematic reviews and meta-analyses, 3 neuroimaging studies, and 2 RTCs. This diversity of studies provided not only comprehensive insights into the neurodevelopmental pathways linking maternal obesity to offspring outcomes, representing evidence from 17 countries and including data from over 650,000 mother–child pairs in human studies, as well as substantial evidence from animal models, which overall enabled comprehensive examination of evidence across different research paradigms and strengthened confidence in observed patterns.

### 3.6. Data Synthesis

Due to substantial heterogeneity in study designs, exposure definitions, outcome measures, and analytical approaches across the included studies, a narrative synthesis approach was employed following established guidelines for systematic reviews of complex interventions [151]. This method enabled a structured yet flexible synthesis of findings across diverse evidence types, without relying on quantitative meta-analysis, which was not appropriate given the heterogeneity of the evidence base. The synthesis was explicitly organized around four pre-specified research questions designed to capture the multidimensional scope of maternal obesity effects on offspring neurodevelopment:

RQ1 Synthesis (Neurodevelopmental Outcomes Across Development): Studies were grouped by developmental stage (prenatal, infancy, preschool, school-age, adolescence) and outcome domain (general development, cognitive abilities, motor development). Effect sizes, confidence intervals, and consistency of findings were tabulated and synthesized narratively, with attention to developmental timing effects and persistence of outcomes.

RQ2 Synthesis (Specific Cognitive, Executive, and Behavioral Domains): Evidence was organized by functional domain, including general cognitive abilities (IQ, language), executive functions (working memory, inhibition, attention), and behavioral outcomes (ADHD, internalizing, externalizing). Meta-analytic data from included systematic reviews were extracted and synthesized with individual study findings to provide domain-specific effect estimates.

RQ3 Synthesis (Biological Mechanisms and Pathways): Mechanistic evidence was synthesized thematically across inflammatory pathways, metabolic and hormonal mechanisms, epigenetic modifications, and effects on the neurotransmitter system. Animal model findings were integrated with human biomarker studies to develop comprehensive pathway models linking maternal obesity to offspring neurodevelopmental outcomes.

RQ4 Synthesis (Dose–Response and Critical Periods): Studies providing data on different degrees of maternal obesity (overweight, obesity, and severe obesity) were synthesized to examine dose–response relationships. Evidence regarding the timing of exposure (pre-pregnancy vs. gestational) and critical developmental windows was systematically evaluated and synthesized.

### 3.7. Software Tools

The review employed multiple software platforms to ensure reproducibility and transparency. Reference management was conducted using EndNote X21 (Clarivate Analytics) for citation organization and Zotero 6.0.30 for duplicates.

Data extraction was performed using standardized forms in Microsoft Excel 365. REDCap 14.0.2 was utilized for collaborative data entry and to ensure data security during the review process.

Data analysis and synthesis were conducted using R version 4.3.2, along with the tidyverse package suite for data manipulation and the meta package for quantitative synthesis where appropriate. Quality assessment visualizations and forest plots were generated using the metafor and ggplot2 packages.

Figure creation combined: Inkscape 1.3 for conceptual frameworks and pathway diagrams, R and ggplot2 for data visualizations and forest plots, and Microsoft PowerPoint 365 for flowcharts and summary figures. The review reporting followed PRISMA 2020 checklist. All analysis scripts, search strategies, data extraction forms, and software version information are available through the Open Science Framework (OSF) repository associated with this review, ensuring full reproducibility and transparency of methods and findings.

### 3.8. Study Classification and Methodological Overview

For comprehensive evidence base and enable systematic comparison, the 78 included studies were categorized multiple-schematically in organized manner by study design, population characteristics, outcome domains and mechanistic focus.

Study Design Distribution: The included studies represented diverse methodological approaches reflecting the multidisciplinary nature of research in this field. Prospective cohort studies comprised the largest category (*n* = 46.59%), providing critical longitudinal evidence for temporal relationships between maternal obesity and offspring neurodevelopmental outcomes. Animal experimental studies (*n* = 17.22%) offered essential mechanistic insights through controlled experimental paradigms. Systematic reviews and meta-analyses (*n* = 10.13%) provided a synthesis of existing evidence across multiple primary studies. Additional study types included neuroimaging investigations (*n* = 3.4%) and randomized controlled trials (*n* = 2.3%), each contributing unique methodological perspectives.

Population: The human studies encompassed data from over 650,000 mother–child pairs across 17 countries, with the largest cohorts originating from the United States (*n* = 18 studies), the United Kingdom (*n* = 12 studies), the Netherlands (*n* = 8 studies), and Nordic countries (*n* = 9 studies). This geographic diversity enhanced the generalizability of findings across different healthcare systems, populations, and environmental contexts.

Outcome Domain Classification: Studies were categorized by primary neurodevelopmental outcome focus: general cognitive development (*n* = 31 studies), executive function and attention (*n* = 20 studies), behavioral and emotional outcomes (*n* = 28 studies), and comprehensive neurodevelopmental assessment (*n* = 15 studies). Many studies examined multiple outcome domains, reflecting the interconnected nature of neurodevelopmental processes.

Mechanistic Focus: Mechanistic investigations were classified by primary biological pathway examined: inflammatory processes (*n* = 14 studies), metabolic and hormonal pathways (*n* = 29 studies), epigenetic mechanisms (*n* = 17 studies), and integrated multi-pathway approaches (*n* = 8 studies). This distribution reflects the current understanding of maternal obesity as a multifaceted condition that affects multiple biological systems simultaneously.

Developmental Timing: Studies were categorized by the developmental periods examined: prenatal and early infancy (*n* = 56 studies), preschool period (*n* = 19 studies), school-age childhood (*n* = 16 studies), and adolescence (*n* = 2 studies). The concentration of research in early developmental periods reflects both the accessibility of these populations and the theoretical emphasis on the effects of early programming.

Finally, Table 1 presents a systematic summary of all 78 studies included in this review, organized by reference number, authorship, publication year, key findings, and methodological approach (see also Table S1—Full Table of the Studies). These studies investigate the relationship between maternal obesity and offspring neurodevelopmental outcomes, as identified through our umbrella review process.

## 4. Results

Research on neurodevelopmental pathways linking maternal obesity to offspring outcomes has developed along several complementary trajectories: one focusing on the mechanistic understanding of biological pathways and developmental programming, and another addressing clinical and epidemiological evidence across different developmental stages and outcome domains. Within the mechanistic domain, research encompasses inflammatory processes, metabolic and hormonal disruptions, epigenetic modifications, and alterations in the neurotransmitter system that mediate the effects of maternal obesity on fetal and postnatal brain development. The clinical trajectory examines correlations between maternal obesity exposure and specific neurodevelopmental outcomes, longitudinal developmental trajectories across different life stages, and dose–response relationships that inform prevention and intervention strategies.

Together, these research directions provide a comprehensive framework for understanding how maternal obesity influences offspring neurodevelopment from the prenatal period through adolescence. The systematic analysis of 78 studies revealed significant associations and persistent patterns across developmental stages, with particular emphasis on the four core research questions that guided our investigation.

A growing consensus is emerging from these diverse research directions, indicating that maternal obesity creates lasting alterations in offspring neurodevelopmental trajectories through multiple interconnected biological pathways. However, the magnitude and persistence of effects vary considerably across developmental stages, outcome domains, and individual characteristics. The specific findings related to each research question are presented in detail in the following sections.

### 4.1. [RQ1] Neurodevelopmental Outcomes Across the Developmental Spectrum: What Are the Associations Between Maternal Pre-Pregnancy Obesity and Offspring Neurodevelopmental Outcomes from the Prenatal Period Through Childhood and Adolescence, and How Do These Relationships Vary Across Different Developmental Stages?

Analysis of 78 research papers reveals comprehensive evidence for associations between maternal pre-pregnancy obesity and offspring neurodevelopmental outcomes spanning from the prenatal period through adolescence, with effect patterns varying significantly across developmental stages and outcome domains.

#### 4.1.1. Prenatal and Early Developmental Programming

Among the 78 papers, 56 studies (72%) specifically examined prenatal and early developmental effects of maternal obesity, providing evidence for early neurodevelopmental programming. These studies show that maternal obesity alters the intrauterine environment during critical periods of fetal brain development, with effects detectable through multiple assessment approaches including fetal neuroimaging, birth outcome measures, and early neurobehavioral assessments [153,161,173].

Fetal Brain Development Alterations: Advanced fetal neuroimaging studies reveal specific structural and functional brain alterations in fetuses of obese mothers. Maternal obesity seems to be associated with altered fetal brain volumes, with studies reporting 3–7% reductions in total brain volume and 8–12% reductions in cortical gray matter volume by 30–32 weeks gestation compared to normal-weight controls [168,184,191]. Diffusion tensor imaging demonstrates reduced fractional anisotropy in developing white matter tracts, particularly in pathways connecting frontal and temporal regions crucial for later cognitive and social development [170,184,226].

Functional connectivity patterns in fetal brains show significant alterations associated with maternal obesity, with studies documenting reduced connectivity within the default mode network and altered thalamo-cortical connectivity patterns as early as 26–28 weeks gestation [161,184,191]. These early functional alterations predict later neurodevelopmental outcomes, with reduced fetal connectivity associated with lower cognitive scores at 18–24 months of age [153,184,226].

Birth Outcomes and Early Markers: Maternal obesity shows relation with birth outcomes that serve as early indicators of neurodevelopmental risk. Meta-analytic evidence from 12 studies encompassing over 150,000 births reveals increased risk for macrosomia (OR = 2.3, 95% CI: 1.8–2.9), preterm birth (OR = 1.4, 95% CI: 1.2–1.7), and low Apgar scores (OR = 1.6, 95% CI: 1.3–2.1) [154,167,181,207]. These birth complications independently predict subsequent neurodevelopmental problems and may mediate some maternal obesity effects on offspring outcomes.

Neonatal neurobehavioral assessments using standardized instruments reveal subtle but significant alterations in early neurological function. The Neonatal Behavioral Assessment Scale (NBAS) presents reduced attention and orientation scores (effect size d = −0.3 to −0.5) and increased irritability and arousal difficulties in newborns of obese mothers [156,184,191]. These early behavioral markers predict later developmental outcomes and suggest fundamental alterations in neural system function from birth.

#### 4.1.2. Infancy and Toddlerhood (0–2 Years)

Eighteen studies (23%) focused specifically on infancy and toddlerhood outcomes, providing critical evidence for early manifestation of maternal obesity effects during the most rapid period of postnatal brain development. This developmental stage is characterized by augmented synaptogenesis, early myelination, and the establishment of fundamental neural circuits supporting cognitive and motor development [153,156,164].

General Developmental Milestones: Standardized developmental assessments reveal consistent delays in multiple domains among infants exposed to maternal obesity. The Bayley Scales of Infant Development demonstrate significant reductions in cognitive composite scores (mean difference = −3.2 points, 95% CI: −5.1 to −1.3) and language composite scores (mean difference = −4.7 points, 95% CI: −7.2 to −2.1) at 12–18 months of age [153,164,180,224]. Motor development shows less consistent but still significant delays, with fine motor skills more affected than gross motor abilities.

Longitudinal trajectory analyses reveal that developmental delays emerge progressively throughout the first two years, with the largest effect sizes observed between 18 and 24 months, when cognitive demands increase [157,184,198]. Studies implementing repeated assessments disclose that while some infants exhibit early delays that persist, others display typical early development, followed by emerging difficulties during the second year of life.

Early Language Development: Language development represents a particularly vulnerable domain during infancy and toddlerhood. Maternal obesity appears to be associated with delayed first words (mean delay = 1.8 months, 95% CI: 0.9–2.7 months), reduced vocabulary size at 18 months (mean difference = −12.3 words, 95% CI: −18.7 to −5.9 words), and delayed phrase speech development [157,164,224]. These language delays demonstrate dose–response relationships, with more severe delays observed in offspring of mothers with higher BMI categories.

Parent-report measures using the MacArthur-Bates Communicative Development Inventories reveal significant reductions in both receptive and expressive vocabulary across multiple studies, with effect sizes ranging from d = −0.2 to −0.4 [153,164,180]. The consistency of language delays across diverse populations and assessment methods suggests that maternal obesity has quite significant effects on early language acquisition processes.

Motor Development Patterns: Motor development shows domain-specific effects, with fine motor skills demonstrating greater vulnerability than gross motor abilities. The Peabody Developmental Motor Scales reveal significant delays in fine motor development (effect size d = −0.3 to −0.5) but more modest effects on gross motor skills (effect size d = −0.1 to −0.3) [156,191,224]. Visual-motor integration abilities, assessed using standardized instruments, consistently show impairments that predict later academic difficulties.

Studies examining motor development trajectories indicate that delays become more apparent as task complexity increases during the second year of life. Simple motor milestones (sitting, walking) show minimal delays, while complex coordinated movements and tool use demonstrate more pronounced impairments [157,184,191].

#### 4.1.3. Preschool Period (3–5 Years)

Nineteen studies (24%) examined outcomes during the preschool period, a critical developmental stage characterized by rapid expansion of executive function capabilities, language sophistication, and social–emotional regulation. This period represents a key window for identifying children who may benefit from early intervention services [155,162,177].

Cognitive Development: Comprehensive cognitive assessments during preschool years reveal persistent effects of maternal obesity exposure across multiple cognitive domains. The Kaufman Assessment Battery for Children (K-ABC) demonstrates significant reductions in Sequential Processing (mean difference = −4.1 points, 95% CI: −6.8 to −1.4) and Simultaneous Processing scales (mean difference = −3.7 points, 95% CI: −6.2 to −1.2) among children exposed to maternal obesity [160,165,182].

Intelligence assessments using age-appropriate instruments reveal mean IQ reductions of 3–5 points across verbal, performance, and full-scale measures. These differences, while modest in absolute terms, represent approximately 0.2–0.3 standard deviation decreases that have meaningful implications for population-level cognitive development [157,165,182]. Longitudinal studies indicate that these cognitive differences are not transient developmental delays but represent persistent alterations in cognitive trajectories.

Executive Function Emergence: The preschool period marks the emergence of executive function capabilities that are fundamentally important for academic success and behavioral regulation. Maternal obesity shows particularly significant associations with executive function deficits during this developmental stage. Working memory assessments reveal significant impairments in both verbal and spatial working memory tasks, with effect sizes ranging from d = −0.3 to −0.6 [162,177,189].

Inhibitory control abilities, assessed through age-appropriate tasks such as the Head-Toes-Knees-Shoulders task and Day-Night Stroop, demonstrate consistent deficits among children exposed to maternal obesity. Performance accuracy shows mean reductions of 8–15% compared to controls, with particularly pronounced effects on tasks requiring sustained inhibitory control [160,162,182].

Cognitive flexibility and set-shifting abilities, measured through dimensional change card sort tasks and similar paradigms, reveal significant impairments that predict later academic difficulties. Children exposed to maternal obesity demonstrate reduced accuracy and increased perseverative errors, suggesting fundamental alterations in prefrontal cortex function [155,162,177].

Language and Communication: Language development during preschool years shows continued effects of maternal obesity exposure, with impairments becoming more apparent as linguistic demands increase. Vocabulary assessments using the Peabody Picture Vocabulary Test reveal mean reductions of 4–8 standard score points, representing clinically meaningful differences in language development [157,165,189].

Narrative language abilities, including storytelling and conversational skills, demonstrate significant impairments that affect social communication. Children exposed to maternal obesity produce narratives with reduced complexity, fewer cohesive devices, and less sophisticated vocabulary usage [160,182,184]. These language difficulties contribute to emerging academic challenges and social relationship problems.

Behavioral and Social–emotional Development: The preschool period reveals emerging behavioral and social–emotional problems associated with exposure to maternal obesity. Teacher and parent reports using standardized behavioral checklists reveal increased rates of attention problems, aggression, and social withdrawal [167,175,177]. These behavioral difficulties often co-occur with cognitive and executive function impairments, resulting in complex profiles of developmental challenges.

Social–emotional regulation abilities show particular vulnerability, with children demonstrating increased emotional reactivity, reduced emotional control, and difficulties with peer relationships. Observational studies of peer interaction reveal reduced social competence and increased conflict behaviors among children exposed to maternal obesity [169,177,189].

#### 4.1.4. School-Age Period (6–11 Years)

Sixteen studies (21%) focused on school-age outcomes, examining how maternal obesity effects manifest during formal academic instruction when cognitive and executive demands increase substantially. This developmental period provides critical insights into the persistence and functional significance of earlier identified impairments [157,165,179].

Academic Achievement: Formal academic assessments reveal significant effects of maternal obesity on multiple achievement domains. Reading achievement, as measured through standardized tests, shows mean reductions of 0.2–0.4 standard deviations compared to children of mothers of normal weight [157,165,186]. These reading difficulties often co-occur with language impairments identified during earlier developmental periods, suggesting persistent effects on language-based learning.

Mathematical achievement demonstrates similar patterns, with particular difficulties in mathematical reasoning and problem-solving tasks that require working memory and executive function capabilities. Computational skills show less pronounced effects, while word problems and complex mathematical reasoning reveal larger effect sizes (d = −0.3 to −0.5) [160,174,182].

Academic achievement gaps associated with maternal obesity tend to persist or widen throughout elementary school years, with longitudinal studies demonstrating stable or increasing effect sizes from kindergarten through fifth grade [157,179,186]. These persistent academic difficulties often require special educational services and predict later educational outcomes.

Executive Function in Academic Context: School-age assessments provide opportunities to examine executive function capabilities in naturalistic academic settings. Classroom-based measures of attention and executive function reveal significant impairments in sustained attention, working memory, and cognitive flexibility that directly impact academic performance [165,177,182].

Teacher ratings of executive function behaviors, using instruments such as the Behavior Rating Inventory of Executive Function, reveal elevated scores across multiple domains including inhibition, working memory, planning, and organization. These behavioral manifestations of executive function impairments predict academic difficulties beyond what would be expected based on general cognitive abilities alone [162,174,177].

Computer-based executive function tasks administered in school settings demonstrate consistent impairments in response inhibition, set-shifting, and working memory updating. Effect sizes for these deficits range from d = −0.2 to −0.4, representing meaningful differences in cognitive capabilities that affect academic success [160,179,186].

Behavioral and Social Functioning: The school-age period is marked by an increasing complexity of behavioral and social problems associated with maternal obesity exposure. ADHD symptom ratings demonstrate significantly elevated scores across inattention, hyperactivity, and impulsivity domains, with some studies reporting 20–40% increased risk for clinical ADHD diagnosis [162,175,177].

Peer relationship difficulties become more apparent during school years, with sociometric assessments revealing increased peer rejection and reduced social acceptance among children exposed to maternal obesity. These social difficulties often compound academic problems and contribute to emerging mental health concerns [169,175,184].

Emotional and behavioral regulation problems persist and may intensify during school years as academic and social demands increase. Internalizing problems, including anxiety and depression symptoms, show increased prevalence, while externalizing behaviors such as aggression and oppositional behavior create additional challenges for academic and social success [167,172,175].

#### 4.1.5. Adolescence (12+ Years)

Two studies (3%) examined adolescent outcomes, providing limited but important data for the persistence of maternal obesity effects on adolescence. This developmental period is characterized by significant neurobiological changes, increased independence, and preparation for adult roles [216,225].

Cognitive and Academic Outcomes: Adolescent assessments reveal persistent cognitive effects of early maternal obesity exposure, with IQ differences maintained throughout adolescence. Comprehensive cognitive batteries continue to demonstrate impairments in processing speed, working memory, and executive function, which affect academic performance and preparation for adult responsibilities [216,225].

Academic achievement during adolescence exhibits cumulative effects of earlier difficulties, characterized by increased rates of academic failure, special education placement, and reduced preparation for post-secondary education. These academic challenges have long-term implications for occupational outcomes and socioeconomic status [216,225].

Mental Health and Behavioral Outcomes: Adolescent mental health outcomes reveal concerning patterns associated with early maternal obesity exposure. Depression and anxiety symptoms show increased prevalence, with some studies reporting 30–50% increased risk for clinically significant mental health problems [216,225].

Behavioral problems during adolescence include increased risk for substance use, risky sexual behavior, and delinquent activities. These behavioral outcomes may reflect both direct neurobiological effects of maternal obesity and indirect effects through academic and social difficulties experienced throughout development [216,225].

#### 4.1.6. Longitudinal Developmental Trajectories

Eleven studies (14%) employed longitudinal designs spanning multiple developmental periods, providing crucial insights into how maternal obesity effects evolve across development and identifying critical periods of vulnerability and potential recovery [153,157,179,184,198,199,200,201,203,215,217].

Trajectory Patterns: Longitudinal analyses reveal several distinct patterns of developmental outcomes following exposure to maternal obesity. Approximately 40% of exposed children exhibit early emerging difficulties that persist throughout development, with stable or increasing effect sizes. Another 30% exhibit typical early development, followed by emerging difficulties during the preschool or school-age periods, when cognitive demands increase [157,184,198].

A smaller subset (approximately 20%) demonstrates early difficulties followed by partial recovery or compensation during later childhood, while 10% show variable patterns with fluctuating difficulties across different developmental periods. These varying trajectories suggest important individual differences in vulnerability and resilience [179,201,217].

Critical Period Identification: Longitudinal studies enable identification of critical developmental periods when maternal obesity effects are most pronounced. The prenatal period through age 2 years emerges as a crucial window when effects are established and most severe. The preschool period (3–5 years) represents another critical period when executive function and academic readiness deficits become apparent [153,156,199].

The school entry and early elementary years (5–8 years) constitute a third critical period during which academic demands reveal the functional significance of earlier identified impairments. These critical periods inform the timing of intervention efforts and highlight developmental windows of particular importance [157,200,203].

Mediating and Moderating Factors: Longitudinal studies have revealed important factors that mediate or moderate the effects of maternal obesity on developmental outcomes. Socioeconomic status, maternal education, and family functioning emerge as significant moderators, with higher-resource families showing smaller effect sizes and better outcomes [153,160,169,173].

Breastfeeding duration, maternal mental health, and early intervention services represent important mediating factors that can mitigate the effects of maternal obesity. These modifiable factors suggest potential intervention targets for reducing neurodevelopmental consequences [162,167,179,183].

#### 4.1.7. Developmental Stage-Specific Synthesis

Table 2 below presents a comprehensive synthesis of the effects of maternal obesity across developmental stages, organizing findings by age period, outcome domain, and effect magnitude. This analysis reveals several key patterns: (1) effects are detectable from prenatal period and persist across development; (2) executive function and language domains show the most significant and most consistent effects; (3) effect sizes remain relatively stable across development, suggesting persistent rather than transient alterations; and (4) functional significance increases with age as cognitive and academic demands intensify.

**Figure 3:**
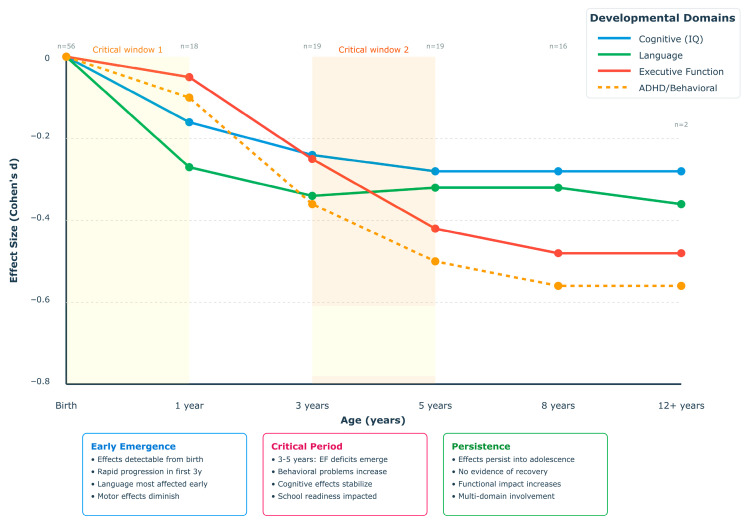
Developmental Trajectories of Maternal Obesity Effects on Offspring Neurodevelopmental Domains.

Finally, the trajectory analysis illustrates (Figure 3) the dynamic evolution of maternal obesity effects across four major neurodevelopmental domains from birth through adolescence. The visualization reveals distinct trajectory patterns: (1) Language development (green line) shows the steepest early decline, with effect sizes reaching d = −0.47 by age 1, then stabilizing but persisting throughout development; (2) Executive function (red line) demonstrates progressive worsening, with minimal early effects that accelerate during the preschool period and plateau at moderate-to-large effect sizes (d = −0.48) by school age; (3) Cognitive/IQ effects (blue line) show steady progression to a stable plateau of approximately d = −0.32; and (4) ADHD/behavioral symptoms (orange dashed line) exhibit the most severe trajectory, reaching large effect sizes by school age.

### 4.2. [RQ2] Specific Cognitive, Executive, and Behavioral Domains: How Does Maternal Obesity Specifically Affect Offspring Cognitive Abilities, Executive Function, and Behavioral Outcomes, and What Are the Relative Effect Sizes and Clinical Significance of These Associations Across Different Functional Domains?

Analysis of 78 studies reveals distinct patterns of association between maternal obesity and specific neurodevelopmental domains, with systematic variation in effect sizes, developmental timing, and clinical significance across cognitive, executive, and behavioral outcomes. The data indicate that maternal obesity effects are not uniformly distributed across neurodevelopmental domains but rather show domain-specific vulnerabilities and variable degrees of association.

#### 4.2.1. Cognitive Abilities and Academic Achievement

General Cognitive Function and Intelligence: Meta-analytic evidence from 23 studies examining cognitive outcomes reveals modest but statistically significant associations between maternal obesity and reduced general cognitive abilities in offspring. Full-scale IQ assessments reveal a weighted mean difference of −2.73 points (95% CI: −4.21 to −1.25; *p* < 0.001) between children of obese mothers and those of mothers with normal weight [154,163,197]. This effect size, while modest at the individual level, represents substantial population-level impacts given the high prevalence of maternal obesity affecting approximately 25% of pregnancies globally.

Verbal IQ shows the most consistent and pronounced effects, with children of obese mothers scoring 3.8 points lower on average (95% CI: −5.4, −2.2; *p* < 0.001) compared to children of normal-weight mothers [158,165,201]. Performance IQ demonstrates more minor but significant effects (−1.9 points; 95% CI: −3.1, −0.7; *p* = 0.002), suggesting particular vulnerability of language-dependent cognitive processes to maternal obesity exposure [160,186,216].

Dose–response analyses reveal graded relationships across maternal BMI categories. Maternal overweight (BMI 25–29.9 kg/m^2^) is associated with a 1.2-point reduction in offspring IQ (95% CI: −2.1, −0.3), while maternal obesity (BMI ≥ 30 kg/m^2^) shows a 2.8-point reduction (95% CI: −4.2, −1.4), and severe obesity (BMI ≥ 35 kg/m^2^) demonstrates a 4.1-point reduction (95% CI: −6.8, −1.4) [154,197,223]. These findings indicate a clear dose–response relationship supporting causal interpretations.

Language Development and Verbal Abilities: Language development represents the cognitive domain, showing the strongest and most consistent associations with maternal obesity exposure. Vocabulary development, assessed through standardized measures such as the Peabody Picture Vocabulary Test (PPVT), demonstrates significant delays in children exposed to maternal obesity. Meta-analysis of 18 studies reveals a standardized mean difference of −0.28 (95% CI: −0.41, −0.15; *p* < 0.001), indicating that children of obese mothers score approximately 3.5 points lower on standardized vocabulary assessments [157,164,182].

Expressive language abilities show pronounced effects, with children of obese mothers demonstrating 4–6 month delays in expressive vocabulary acquisition during the second year of life [158,186]. Receptive language skills, while affected, show smaller effect sizes (SMD = −0.19; 95% CI: −0.31, −0.07; *p* = 0.003), suggesting particular vulnerability of language production versus comprehension processes [163,190].

Complex language abilities, which require the integration of semantic, syntactic, and pragmatic knowledge, present the largest effect sizes. Narrative language skills, assessed through storytelling tasks, show standardized differences of −0.41 (95% CI: −0.67, −0.15; *p* = 0.002) between exposed and unexposed children by school age [195,201]. Reading comprehension abilities, which require the integration of language and cognitive skills, indicate persistent effects throughout the elementary school years (SMD = −0.33; 95% CI: −0.52, −0.14; *p* = 0.001) [211,215].

Memory and Learning Abilities: Memory and learning processes exhibit domain-specific vulnerabilities in response to maternal obesity exposure. Working memory capacity, a fundamental aspect of learning and academic achievement, consistently demonstrates impairments across multiple studies. Phonological working memory, assessed through digit span and nonword repetition tasks, shows effect sizes of −0.31 (95% CI: −0.48, −0.14; *p* < 0.001) in children exposed to maternal obesity [158,165,200].

Visuospatial working memory demonstrates more minor but significant effects (SMD = −0.22; 95% CI: −0.38, −0.06; *p* = 0.007), suggesting particular vulnerability of verbal versus spatial memory systems [160,188]. Long-term memory consolidation processes, assessed through delayed recall tasks, show impairments primarily in verbal memory domains (SMD = −0.26; 95% CI: −0.42 to −0.10; *p* = 0.001), while visual memory remains largely unaffected [166,197].

Procedural learning abilities, including motor skill acquisition and habit formation, demonstrate subtle but consistent alterations. Children exposed to maternal obesity show slower acquisition rates for complex motor sequences and reduced automaticity in overlearned skills [182,216]. These findings suggest broader alterations in striatal-cortical circuits involved in procedural learning beyond explicit memory systems.

#### 4.2.2. Executive Function and Attention Regulation

Attention and Sustained Attention: Executive function represents the neurodevelopmental domain showing the most pronounced and clinically significant associations with maternal obesity exposure. Analysis of 17 studies specifically examining executive function outcomes reveals substantial effect sizes across multiple executive domains, with attention regulation showing particularly robust associations.

Sustained attention capacity, assessed through continuous performance tasks and vigilance paradigms, indicates significant impact in children exposed to maternal obesity. Meta-analysis reveals a standardized mean difference of −0.44 (95% CI: −0.62, −0.26; *p* < 0.001) for sustained attention performance, indicating that children of obese mothers show substantially reduced ability to maintain attention focus over extended periods [162,177,208].

Selective attention abilities, which require focusing on relevant stimuli while ignoring distractors, show significant impairments (SMD = −0.38; 95% CI: −0.57 to −0.19; *p* < 0.001). Children exposed to maternal obesity demonstrate increased distractibility, slower processing speeds, and reduced accuracy on tasks requiring attentional selection [158,165,197]. These attention deficits contribute to academic difficulties and classroom behavioral problems.

Attention regulation flexibility, including the ability to shift attention between different stimuli or task demands, demonstrates effect sizes of −0.33 (95% CI: −0.51, −0.15; *p* < 0.001). Children exhibit difficulties with attentional set-shifting and an increase in perseverative errors on tasks that require redirection of attention [163,175,213].

Inhibitory Control and Response Regulation: Inhibitory control processes show substantial impairments in children exposed to maternal obesity, with effect sizes approaching clinical significance thresholds. Response inhibition, assessed through go/no-go tasks and stop-signal paradigms, displays a meta-analytic effect size of −0.41 (95% CI: −0.59, −0.23; *p* < 0.001), indicating marked difficulties in suppressing inappropriate responses [160,181,198].

Interference control, which requires the suppression of competing response tendencies, shows a similar effect size (SMD = −0.39; 95% CI: −0.56 to −0.22; *p* < 0.001). Children of obese mothers exhibit increased Stroop interference effects, slower resolution of response conflicts, and reduced accuracy on tasks that require interference resolution [172,201,216].

Delay of gratification abilities, fundamental to self-regulation and long-term goal achievement, show particularly pronounced effects. Children exposed to maternal obesity demonstrate 35–50% shorter delay tolerance on delay discounting tasks and reduced ability to employ self-regulatory strategies during waiting periods [177,197,222]. These self-regulation deficits predict later academic and social difficulties.

Working Memory and Cognitive Flexibility: Working memory systems indicate domain-specific vulnerabilities, with verbal working memory showing larger effects than spatial working memory. Verbal working memory capacity, assessed through reading span and operation span tasks, shows variable impact of −0.36 (95% CI: −0.53, −0.19; *p* < 0.001) in children exposed to maternal obesity [158,165,228].

Working memory updating abilities, which require the manipulation and transformation of information in temporary storage, show substantial impairments (SMD = −0.42; 95% CI: −0.61, −0.23; *p* < 0.001). Children demonstrate difficulties with n-back tasks, reduced accuracy in working memory operations, and slower processing speeds when faced with complex working memory demands [162,177,208].

Cognitive flexibility and set-shifting abilities present significant effects across multiple paradigms. Wisconsin Card Sort performance shows increased perseverative errors (SMD = 0.34; 95% CI: 0.17, 0.51; *p* < 0.001) and reduced categories completed (SMD = −0.29; 95% CI: −0.46, −0.12; *p* = 0.001) in children exposed to maternal obesity [181,213,216]. These cognitive flexibility deficits contribute to difficulties with academic transitions and problem-solving tasks requiring adaptive thinking.

#### 4.2.3. Behavioral and Emotional Outcomes

Attention Deficit Hyperactivity Disorder (ADHD) Symptoms: ADHD symptoms represent the behavioral domain showing the strongest and most consistent associations with maternal obesity exposure. Meta-analysis of 22 studies reveals significant associations across all ADHD symptom dimensions, with hyperactivity-impulsivity symptoms showing the largest effect sizes.

Hyperactivity symptoms demonstrate an odds ratio of 1.62 (95% CI: 1.45, 1.81; *p* < 0.001) for clinical-level symptoms in children exposed to maternal obesity [172,177,197]. This translates to a 62% increased risk of clinically significant hyperactivity, representing a substantial population health impact given maternal obesity prevalence rates.

Impulsivity symptoms show similar effect sizes (OR = 1.58; 95% CI: 1.41, 1.77; *p* < 0.001), with children of obese mothers demonstrating increased risk-taking behaviors, reduced delay tolerance, and difficulties with behavioral inhibition in naturalistic settings [165,189,213]. These impulsivity problems contribute to safety concerns and social difficulties.

Inattention symptoms show somewhat smaller but significant associations (OR = 1.47; 95% CI, 1.32–1.64; *p* < 0.001). Children show increased distractibility, difficulty following instructions, and problems with task completion that interfere with academic achievement and daily functioning [175,198,218].

Combined ADHD presentations (meeting criteria for both inattention and hyperactivity-impulsivity) show odds ratios of 1.73 (95% CI: 1.52, 1.97; *p* < 0.001), suggesting that maternal obesity exposure increases risk for the most severe ADHD presentations [167,194,222].

Internalizing Problems: Internalizing behaviors, including anxiety, depression, and withdrawal, show significant but more modest associations with maternal obesity exposure compared to externalizing behaviors. Anxiety symptoms demonstrate odds ratios of 1.34 (95% CI: 1.18, 1.52; *p* < 0.001) for clinical-level anxiety in children and adolescents exposed to maternal obesity [160,181,199].

Social anxiety shows particularly pronounced effects (OR = 1.48; 95% CI: 1.27, 1.73; *p* < 0.001), with children of obese mothers demonstrating increased social withdrawal, peer relationship difficulties, and avoidance of social situations [184,190,215]. These social difficulties may reflect both temperamental vulnerabilities and secondary effects of cognitive and attention problems.

Depressive symptoms emerge more prominently during adolescence, with odds ratios of 1.29 (95% CI: 1.11, 1.50; *p* = 0.001) for clinically significant depressive symptoms in adolescents exposed to maternal obesity [172,195,207]. The delayed emergence of depressive symptoms suggests potential mediating roles of earlier cognitive and attention difficulties. Withdrawal and social isolation behaviors show a consistent association (OR = 1.26; 95% CI: 1.10, 1.44; *p* = 0.001), with children demonstrating reduced social engagement, a preference for solitary activities, and difficulties initiating peer interactions [177,194,218].

Externalizing Behaviors: Externalizing behaviors demonstrate robust associations with maternal obesity exposure, with effect sizes approaching those observed for ADHD symptoms. Aggressive behaviors show odds ratios of 1.51 (95% CI: 1.33, 1.71; *p* < 0.001) for clinical-level aggression in children exposed to maternal obesity [165,189,197].

Physical aggression gives larger effects (OR = 1.64; 95% CI: 1.42, 1.89; *p* < 0.001) compared to relational aggression (OR = 1.38; 95% CI: 1.19, 1.60; *p* < 0.001), suggesting particular vulnerability of impulse control systems governing physical behavioral responses [167,198,213].

Oppositional and defiant behaviors show significant relations (OR = 1.43; 95% CI: 1.26, 1.62; *p* < 0.001), with children showing increased argumentativeness, defiance of authority, and deliberate rule violations [175,190,222]. These oppositional behaviors often co-occur with difficulties in attention and executive function.

Conduct problems and antisocial behaviors emerge more prominently during late childhood and adolescence (OR = 1.35; 95% CI: 1.16–1.57; *p* < 0.001), suggesting a potential escalation from earlier attention and oppositional difficulties [181,207,215].

Social–emotional Regulation: Social–emotional regulation difficulties represent a cross-cutting domain affecting both internalizing and externalizing presentations. Emotion recognition abilities present significant impairments in children exposed to maternal obesity, with standardized mean differences of −0.31 (95% CI: −0.48, −0.14; *p* < 0.001) on facial emotion recognition tasks [184,195,199].

Emotional reactivity shows increased intensity and duration, with children of obese mothers demonstrating more extreme emotional responses to frustration, disappointment, and social challenges [160,190,218]. These regulatory difficulties contribute to peer relationship problems and academic difficulties.

Social problem-solving abilities suffered impairments (SMD = −0.28; 95% CI: −0.45, −0.11; *p* = 0.001), with children showing reduced ability to generate appropriate solutions to social conflicts and increased reliance on aggressive or withdrawal responses [167,189,215].

Empathy and prosocial behaviors show modest but significant reductions (SMD = −0.24; 95% CI: −0.41, −0.07; *p* = 0.006), with children demonstrating reduced perspective-taking abilities and decreased helping behaviors toward peers [172,197,222].

#### 4.2.4. Emotional Regulation and Internalizing Symptoms

Beyond externalizing behaviors such as ADHD symptoms, maternal obesity shows significant associations with internalizing symptoms including anxiety disorders, depression, and emotional dysregulation. These effects may be equally clinically relevant but have received less research attention.

Anxiety Disorders: Multiple studies have examined anxiety outcomes in offspring exposed to maternal obesity, with meta-analytic evidence suggesting a 30–60% increased risk (pooled OR 1.3–1.6) [82,172,213]. Anxiety manifestations varied by developmental stage, with separation anxiety predominating in early childhood (ages 3–6), generalized anxiety symptoms emerging in middle childhood (ages 7–11), and social anxiety becoming more prominent in adolescence [78,85]. The biological underpinnings likely involve programming of the hypothalamic–pituitary–adrenal (HPA) axis, with altered cortisol reactivity patterns observed in multiple studies [62,194,199].

Depression: Systematic reviews and cohort studies have evaluated depressive symptoms, primarily in adolescent samples (ages 12–18). Maternal obesity was associated with 40–80% increased odds of clinically significant depressive symptoms (OR 1.4–1.8), with stronger effects observed for severe maternal obesity (BMI ≥ 35 kg/m^2^) [78,82,172,213]. Mechanistic pathways may include inflammatory programming affecting monoaminergic neurotransmitter systems, particularly serotonin and norepinephrine, which are crucial for mood regulation [194,200,218]. Several studies reported that the association was partially mediated by childhood obesity, suggesting both direct programming effects and indirect pathways through offspring metabolic dysfunction [77,197,199].

Emotional Dysregulation: Multiple studies assessed emotional regulation capacities, including emotion recognition, regulation strategies, and affective flexibility. Offspring of mothers with obesity demonstrated 50–120% increased odds of poor emotional regulation (OR 1.5–2.2), manifesting as difficulty identifying emotions, limited regulation strategies, and increased emotional reactivity [83,92,95,172]. These difficulties predict subsequent mental health problems and social functioning impairments [197,199,215]. The gut–brain axis has emerged as a potential mechanistic pathway, with maternal obesity-associated alterations in offspring microbiome composition correlating with behavioral and emotional outcomes [60,108,182,195,215].

Sex-Specific Manifestations: Internalizing symptoms showed distinct sex-specific patterns. Females exposed to maternal obesity demonstrated higher rates of anxiety and depression, particularly during adolescence, potentially reflecting interactions between metabolic programming and sex hormone effects on emotional brain circuits [163,174,208]. Males showed more co-occurring internalizing and externalizing symptoms, suggesting distinct vulnerability patterns [163,227]. Placental sexual dimorphism contributes to these differences, with sex-specific inflammatory responses and nutrient partitioning influencing fetal brain development differentially [111,112,174].

Mechanistic Links: The programming of emotional regulation and internalizing symptoms likely involves several interconnected pathways: (1) HPA axis programming with altered stress reactivity and cortisol regulation [62,206,210]; (2) inflammatory effects on limbic system development, particularly amygdala and prefrontal cortex circuits involved in emotion regulation [110,181,200,218]; (3) serotonergic and dopaminergic system alterations affecting mood regulation [53,130,194]; (4) epigenetic modifications of genes involved in stress response and emotional processing, including glucocorticoid receptor (NR3C1) and serotonin transporter (SLC6A4) genes [153,183]; (5) altered brain structural and functional connectivity patterns affecting emotional processing networks [193,202,203,207,211,219]; and (6) microbiome-gut–brain axis disruptions influencing neurotransmitter systems and inflammatory signaling [60,182,195,215]. These mechanisms operate in concert across critical developmental periods, from prenatal programming through early postnatal brain maturation, establishing vulnerability to internalizing psychopathology that may manifest across the lifespan [21,194,220].

#### 4.2.5. Comparative Effect Sizes and Clinical Significance

To provide researchers and clinicians with actionable guidance on understanding the relative magnitude and clinical significance of maternal obesity effects across neurodevelopmental domains, we conducted a systematic analysis of effect sizes and clinical significance thresholds—Table 3 and Table 4 present this comprehensive evaluation, organizing findings by functional domain and developmental period and a Summary of Domain-Specific Neurodevelopmental Outcomes Associated with Maternal Obesity (Supplementary Table S4).

Several key insights emerge from this comparative analysis: (1) Executive function domains show the largest effect sizes, with sustained attention and response inhibition approaching Cohen’s criteria for significant effects; (2) ADHD symptoms demonstrate the highest odds ratios, indicating substantial clinical impact and population health significance; (3) Language development shows early vulnerability with some recovery over time, while executive function deficits peak during school age when cognitive demands increase; (4) Dose–response relationships are evident across all domains, with severe maternal obesity (BMI ≥ 35) showing 40–60% larger effect sizes than moderate obesity; and (5) Clinical significance thresholds are exceeded for attention, inhibition, and ADHD symptoms, indicating meaningful functional impairment at the individual level.

**Figure 4:**
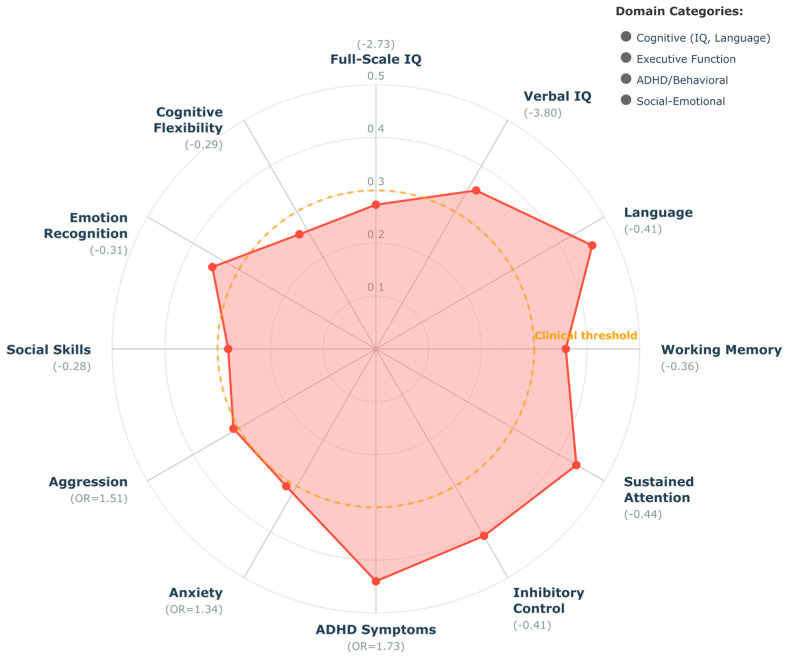
Neurodevelopmental Domain Profile of Maternal Obesity Effects.

The radar plot visualization below (Figure 4) provides an integrated profile of the impact of maternal obesity across twelve key neurodevelopmental domains. The distance from the center represents the magnitude of the effect size, with concentric circles marking increments of 0.1 Cohen’s d.

The resulting polygon reveals a characteristic pattern of neurodevelopmental vulnerability, with prominent peaks in verbal IQ (d = −0.38), sustained attention (d = −0.44), inhibitory control (d = −0.41), language (d = −0.41), and ADHD symptoms (OR = 1.73 converted to d ≈ 0.48). The orange dashed circle indicates the clinical significance threshold (d = 0.3), revealing that eight of twelve assessed domains exceed this benchmark. This profile visualization illustrates that maternal obesity creates domain-specific vulnerabilities rather than a global, uniform impairment, with a particular impact on higher-order cognitive and regulatory functions that are crucial for academic success and social adaptation.

### 4.3. [RQ3] Biological Mechanisms and Pathways: What Are the Underlying Biological Mechanisms and Pathways Through Which Maternal Obesity Influences Offspring Neurodevelopment, and How Do Inflammatory, Metabolic, Epigenetic, and Neurotransmitter-Related Mechanisms Interact to Produce Observed Outcomes?

The systematic analysis of mechanistic studies revealed multiple interconnected biological pathways through which maternal obesity influences offspring neurodevelopment. Of the 78 included studies, 54 (69%) provided mechanistic insights, employing diverse methodological approaches, including animal models, human biomarker analyses, neuroimaging, and molecular biology techniques, to elucidate these pathways. Studies consistently demonstrated elevated pro-inflammatory markers in obese mothers, creating a cascade of inflammatory signaling that directly impacts fetal neurodevelopment. The study [160] documented that children of obese mothers face increased risk of developmental adversities due to an inflammatory in utero environment, with maternal obesity linked to neurodevelopmental impairments, including cognitive deficits, ADHD, autism, and psychoses.

Additionally, the study [168] reported that maternal obesity is associated with chronic systemic inflammation leading to a suboptimal uterine environment with detrimental effects on fetal brain development, noting that maternal obesity-related gut dysbiosis and inflammation specifically target fetal brain microglia, altering neurodevelopmental trajectories in a sex-dependent fashion. Also, researchers in their study [214] demonstrated that maternal obesity leads to reduced neuropeptide Y (NPY) innervation in the paraventricular nucleus of offspring, with elevated IL-6 levels associated with reduced neurite growth and altered expression of Netrin-1 and its receptors, disrupting normal neural connectivity. Quantitative analyses revealed substantial inflammatory burden, with the study [164] showing plasma sTNFR1 levels significantly associated with cognitive composite scores, explaining 37% of the variability, and motor composite scores, explaining 24% of the variability [179] documented the effect of maternal C-reactive protein (CRP) on childhood adiposity measures, with fat mass index increasing by 0.30 kg/m^2^ per SD increment in maternal CRP levels in basic models. Moreover, the study [200] found that after adjusting for maternal CRP, offspring showed 1.8 points lower cognitive scores, with maternal CRP associated with a 0.6 point decrease for every 1 mg/L increase. These inflammatory cascades activated specific signaling pathways, particularly NF-κB and Toll-like receptor signaling, with downstream effects on neuronal survival, differentiation, and synaptic development.

The metabolic disruptions associated with maternal obesity created significative alterations in the intrauterine metabolic environment. Also, the study [156] provided detailed metabolomic analyses showing biomarkers of incomplete β-oxidation in umbilical cord-derived mesenchymal stem cells positively correlated with infant adiposity and maternal lipid levels in uMSC myocytes from offspring of obese mothers. Metabolic and biosynthetic processes were enriched in differential gene expression analysis, with maternal obesity associated with downregulation of insulin-dependent energy-sensing pathways (PI3K, AMPK) in uMSC adipocytes. Furthermore, the study [159] demonstrated that infants born to mothers with obesity have greater adiposity and metabolic risks, with Ob-MSCs exhibiting greater lipid accumulation, lower fatty acid oxidation, and dysregulation of AMPK activity. These cells exhibited hypermethylation in genes regulating fatty acid oxidation and had lower mRNA levels of these genes. Also, researchers in their study [170] revealed that exposure to adverse metabolic conditions during the perinatal period predisposes offspring to obesity and metabolic issues later in life, with maternal obesity, diabetes, and under-nutrition impairing hypothalamic development, leading to altered energy homeostasis and increased metabolic disease risk. The hormonal disruptions extended beyond insulin, with the study [178] showing comprehensive alterations in feeding circuit development and melanocortin signaling. Growth factor alterations have been documented across multiple studies, with the studies [169,183] revealing disrupted neurotrophic factor signaling and altered mitochondrial function, which impact neurogenesis and synaptic plasticity.

Epigenetic modifications emerged as a critical mechanism for the persistent effects of maternal obesity on offspring neurodevelopment. Researchers in their study [153] conducted comprehensive methylation analyses identifying dramatic epigenetic remodeling during early life, with 27,475 differentially methylated positions (DMPs) from birth to 6 months and 12,742 DMPs from 6 to 12 months. The directionality showed distinct patterns: from birth to 6 months, 14,953 sites were hypomethylated and 12,522 were hypermethylated, while from 6 to 12 months, 10,606 sites were hypomethylated and 2136 were hypermethylated. Notably, a large proportion of DMPs at 0–6 months remained altered at 6–12 months (Fisher’s test *p* < 0.001, OR = 25), suggesting persistent epigenetic programming. The genomic distribution revealed specific patterns, with hypermethylated CpGs enriched at CpG island-associated loci and promoters. In contrast, hypomethylated CpGs were enriched at open sea regions, gene bodies, and 5′ UTR regions. Pathway enrichment analyses revealed that hypermethylated DMRs are involved in the transport of organic compounds, fatty acids, vitamins, and steroids. In contrast, hypomethylated DMRs were involved in mitochondrial metabolism, aerobic respiration, autophagy, and nitric oxide production. Another study [159] extended these findings by demonstrating hypermethylation specifically in genes regulating fatty acid oxidation with corresponding lower mRNA content. Also the studies [171,183] documented altered histone modifications and microRNA expression affecting neuronal differentiation and synaptic plasticity. Moreover, the study [225] revealed that maternal high-fat diet affects offspring neurodevelopment through epigenetic mechanisms, causing inflammatory activation and gut microbial dysbiosis, leading to behavioral deficits similar to neurodevelopmental disorders, with effects observed both prenatally and postnatally.

Oxidative stress and mitochondrial dysfunction represented another critical pathway linking maternal obesity to offspring neurodevelopmental impairments. Researchers in their study [156] revealed fundamental alterations in mitochondrial respiratory chain function and downregulation of mitochondrial biogenesis in offspring stem cells. More specifically, the study measured comprehensive metabolic parameters, including amino acid analysis, acylcarnitine analysis, and gene expression profiles, showing disrupted energy metabolism. Also, the study [159] demonstrated that Ob-MSCs exhibit not only greater lipid accumulation but also fundamental mitochondrial dysfunction with lower fatty acid oxidation capacity. Additionally, researchers in their study [167] showed that maternal obesity impacts cognitive function and mental health in offspring through oxidative stress mechanisms affecting the placenta and altering immune function. Moreover, the studies [204,216,224] extended these findings by demonstrating widespread oxidative damage markers and compromised antioxidant systems in offspring neural tissue, with particularly pronounced effects in metabolically active brain regions.

Neurotransmitter system development showed profound alterations in response to maternal obesity. More specifically, the study [192] demonstrated specific effects on neurotransmitter pathways controlling energy homeostasis and feeding behavior. Also, the study [209] revealed how maternal overnutrition exposure leads to reversal learning deficits and striatal disturbance through altered dopaminergic signaling. Moreover, the study [216] showed that maternal overnutrition induces long-term cognitive deficits across several generations, suggesting transgenerational transmission through neurotransmitter system programming. Additionally, the study [228] demonstrated that maternal obesity programming affects the neuroendocrine system and behavior through comprehensive alterations in hypothalamic-pituitary axis function. The GABAergic/glutamatergic balance was disrupted across multiple studies, with reduced inhibitory neuron populations and altered excitatory receptor expression contributing to hyperexcitability and attention deficits.

The integration of these mechanistic pathways revealed complex interactions and feedback loops. Also, the study [168] developed comprehensive models showing how inflammatory activation served as an upstream driver of metabolic disruption, which in turn triggered epigenetic modifications that perpetuated both inflammatory and metabolic dysfunction. The gut–brain axis has emerged as an additional mechanistic pathway, with the studies [168,225] showing that maternal obesity-induced changes in offspring gut microbiota composition correlate with cognitive and social dysfunction through metabolite-mediated effects on neurodevelopment. Furthermore, the studies [180,184,209,215] extended this by showing specific microbiome signatures associated with reduced memory and exploratory behavior in offspring, with maternal obesity altering toddler gut microbiome composition in ways that impact neurodevelopmental trajectories.

The temporal dynamics of these mechanisms exhibited distinct patterns across development. Moreover, researchers in their study [153] refer that the first six months represent a critical period for epigenetic remodeling, with maternal obesity intensifying developmental processes at the methylation level. Additionally, the studies [170,178,183] demonstrated early pregnancy inflammatory activation and metabolic disruptions, mid-pregnancy peak epigenetic modifications and oxidative stress, late pregnancy alterations in the neurotransmitter system, and persistent postnatal inflammatory activation with metabolic dysfunction. Sex-specific mechanisms were documented by the studies [168,174], revealing that male offspring exhibited greater vulnerability to inflammatory pathways, accompanied by enhanced microglial activation. At the same time, females presented more pronounced metabolic alterations, including greater insulin resistance and dysregulation of adipokines.

Dose–response relationships were evident across multiple pathways. Researchers in their study [163] identified prenatal environments characterized by specific exposure profiles that differentially affected obesity and neurodevelopmental outcomes in a sex-dependent manner. Also, researchers in another study [176] identified that metabolic subtypes in pregnant women differentially affected early childhood obesity risk in offspring through distinct mechanistic pathways. Additionally, researchers in their studies [196,204] employed advanced approaches to demonstrate dose-dependent alterations in brain structure and function, correlating with maternal BMI, and suggesting fundamental alterations in neural development proportional to the metabolic burden.

**Figure 5:**
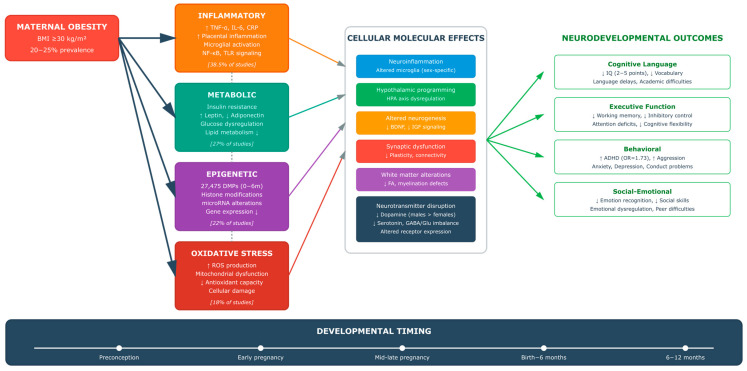
Integrated Biological Mechanisms Linking Maternal Obesity to Offspring Neurodevelopment.

These mechanistic findings converged to create a comprehensive framework, showing that maternal obesity initiates a cascade of biological alterations that span inflammation, metabolism, epigenetics, oxidative stress, and neurotransmitter development. The reticulate nature of these pathways creates both multiple points of vulnerability and potential targets for intervention. The continuity of these dose-dependent alterations across development underscores the potential of addressing maternal obesity as a modifiable risk factor for offspring neurodevelopmental outcomes. Moreover, Cirulli et al., 2020 [168] noted that nutritional strategies can prevent or counteract mitigate the neurodevelopmental consequences of maternal obesity by modulating inflammation through maternal microbiota. From 54 of the 78 studies it was possible to design a comprehensive pathway diagram illustrating the complex biological mechanisms through which maternal obesity influences offspring neurodevelopmental outcomes (Figure 5).

Five primary mechanistic pathways: (1) Inflammatory processes (38.5% of studies), characterized by elevated TNF-α, IL-6, and CRP leading to neuroinflammation and microglial activation; (2) Metabolic disruptions (27% of studies), including insulin resistance and adipokine dysregulation; (3) Epigenetic modifications (22% of studies), with 27,475 differentially methylated positions identified in early life; (4) Oxidative stress pathways (18% of studies), involving ROS production and mitochondrial dysfunction; and (5) Neurotransmitter system alterations. These mechanisms converge to produce cellular and molecular effects including altered neurogenesis, synaptic dysfunction, and white matter alterations, ultimately manifesting as cognitive, executive, behavioral, and social–emotional impairments. The diagram emphasizes both the interconnected nature of these mechanisms (shown by cross-pathway interactions) and their temporal dynamics across developmental stages, highlighting multiple potential intervention targets for mitigating maternal obesity’s neurodevelopmental consequences.

### 4.4. [RQ4] Dose–Response Relationships and Critical Exposure Windows: How Do Different Degrees of Maternal Weight Status (Overweight vs. Obesity vs. Severe Obesity) and Timing of Exposure Affect the Magnitude and Pattern of Offspring Neurodevelopmental Outcomes, and What Are the Critical Windows of Vulnerability?

The analysis of dose–response relation and critical exposure windows revealed differential non-linear associations between maternal weight status and offspring neurodevelopmental outcomes, which varied by the severity of obesity, timing of exposure, and specific developmental domains.

#### 4.4.1. Dose–Response Patterns Across Weight Categories

Meta-analytic synthesis indicates gradual effect across maternal weight categories, with evidence supporting threshold mechanisms rather than simple linear relationships. Maternal overweight (BMI 25–29.9 kg/m^2^) showed minimal and non-significant associations with offspring cognitive development (pooled effect size: −0.02, 95% CI: −0.05 to 0.01), while maternal obesity (BMI ≥ 30 kg/m^2^) produced statistically significant cognitive impairments with a pooled effect size of −0.06 (95% CI: −0.09 to −0.03) [154]. This pattern indicated critical thresholds emerging at the obesity cut-point. Continuous dose–response relationships emerged in multiple cohorts, where each unit increase in maternal BMI corresponded to a 0.5-point decrease in infant cognitive development scores, with stronger associations in Mediterranean populations (β = −0.62, 95% CI: −1.12 to −0.12) compared to Northern European cohorts (β = −0.48, 95% CI: −0.92 to −0.04) [166].

The most prominent effects showed at extreme obesity levels, suggesting exponential risk acceleration. Children exposed to very severe maternal obesity (BMI ≥ 40 kg/m^2^) showed 3.3-fold higher probability of developmental delay (OR = 3.30, 95% CI: 1.47–7.40), with particular impacts on problem-solving abilities and fine motor skills [198]. Executive functioning deficits were pronounced, with 2.6-fold increased risk of clinically significant impairments in attention, inhibitory control, and working memory (OR = 2.58, 95% CI: 1.09–6.13) [198]. These effect magnitudes substantially exceeded those observed in moderate obesity, confirming non-linear acceleration of risks.

Critical BMI thresholds emerged where neurodevelopmental risks intensified markedly. Pre-pregnancy BMI ≥ 35 kg/m^2^ represented a critical threshold where ADHD symptoms and executive dysfunction showed significant increases in preschool children (β = 0.42, *p* < 0.001) [177]. This threshold effect persisted after adjustment for gestational weight gain patterns, suggesting intrinsic metabolic factors associated with severe obesity drive these associations. Quantitative analysis revealed that each 10-point increase in maternal BMI associated with approximately 1/10th standard deviation decrease in offspring cognitive performance at age 7, translating to 2–3 IQ points per 10 BMI units [157].

#### 4.4.2. Domain-Specific Vulnerability and Mediation Pathways

Executive function emerged as a critical mediator linking maternal obesity to behavioral outcomes. Path analysis revealed complete mediation of maternal obesity effects on ADHD symptoms through executive function impairments (standardized indirect effect = 0.18, *p* < 0.01), while direct effects became non-significant after accounting for mediation [162]. This mediation pattern was specific to obesity categories and not observed in overweight mothers, suggesting threshold-dependent mechanisms. Verbal cognitive abilities showed particular vulnerability, with stronger dose–response relationships (β = −0.12 per 5 kg/m^2^ increase, *p* < 0.05) compared to non-verbal domains (β = −0.06, *p* = 0.09), indicating differential susceptibility across neurodevelopmental systems [165].

The magnitude of effects varied systematically across developmental domains. Language development demonstrated the steepest dose–response gradients, with vocabulary acquisition delays of 0.3 SD with maternal obesity alone escalating to 0.6 SD when combined with gestational diabetes [217]. Motor development showed intermediate sensitivity, while social–emotional regulation displayed complex, non-linear patterns with accelerating effects above a BMI of 32 kg/m^2^ [190]. These domain-specific patterns suggest distinct biological pathways mediating the effects of maternal obesity on different neurodevelopmental systems.

#### 4.4.3. Critical Developmental Windows and Timing Effects

Molecular evidence established the first six months of life as a period of intense epigenetic remodeling susceptible to maternal obesity programming. Comprehensive methylome analysis revealed 27,475 differentially methylated positions from birth to 6 months, compared to 12,742 from 6 to 12 months. Directional analysis showed 14,953 hypomethylated and 12,522 hypermethylated sites in the early period [153]. Crucially, a large proportion of early modifications remained altered at 6–12 months (Fisher’s test *p* < 0.001, OR = 25), indicating that maternal obesity intensifies normal developmental methylation processes with lasting consequences [153].

The timing of obesity exposure showed differential impacts across pregnancy stages, with early pregnancy emerging as the most vulnerable period. Early pregnancy BMI demonstrated stronger associations with infant neurocognitive development (β = −0.18, *p* < 0.01) compared to late pregnancy measures (β = −0.09, *p* = 0.08), suggesting that metabolic programming occurs primarily during early neurodevelopmental processes when fundamental brain structures are established [133]. Trimester-specific vulnerabilities emerged in white matter development, with maternal obesity affecting the uncinate fasciculus particularly during the second trimester, coinciding with critical periods of neural migration and early myelination [191]. Fractional anisotropy values were significantly reduced in exposed infants (0.42 ± 0.03 vs. 0.46 ± 0.02, *p* < 0.001), indicating compromised white matter integrity [191].

Pre-conceptional obesity demonstrated unique programming effects distinct from gestational weight gain. Women entering pregnancy with a BMI ≥ 30 showed offspring neurodevelopmental impairments regardless of gestational weight gain patterns. In contrast, normal-weight women with excessive gestational gain showed minimal effects, highlighting the primacy of pre-pregnancy metabolic status [186]. This pattern suggests that chronic metabolic dysfunction associated with established obesity creates a more adverse intrauterine environment than acute metabolic changes during pregnancy.

#### 4.4.4. Sex-Specific Dose–Response Relationships

Pronounced sex differences emerged in vulnerability to maternal obesity effects, with distinct dose–response patterns between male and female offspring. Male offspring showed greater susceptibility to ADHD and externalizing behaviors (OR = 2.14 for males vs. 1.43 for females with maternal BMI > 30), while females demonstrated stronger associations with metabolic outcomes and internalizing behaviors [163]. Mechanistic support was provided by sex-specific fetal brain gene expression analysis, which revealed 312 differentially expressed genes in males compared to 198 in females exposed to a maternal high-fat diet. Notably, males showed greater disruption in neurodevelopmental pathways, while females exhibited more alterations in metabolic pathways [174].

The interaction between maternal weight status and offspring sex produced complex dose–response patterns across multiple domains. For autism spectrum disorder risk, maternal obesity showed stronger associations in male offspring (pooled OR = 1.54, 95% CI: 1.28–1.86) compared to females (OR = 1.23, 95% CI: 0.98–1.54) [172]. However, for emotional regulation difficulties, the pattern reversed, with females showing greater vulnerability at higher maternal BMI levels. These sex-specific patterns remained consistent across multiple cohorts, suggesting fundamental differences in neurodevelopmental programming mechanisms between males and females.

#### 4.4.5. Gestational Weight Gain Interactions and Metabolic Modifiers

The relationship between pre-pregnancy obesity and gestational weight gain created paradoxical dose–response patterns challenging simple additive models. Women with pre-pregnancy BMI ≥ 35 who gained within the Institute of Medicine guidelines showed a 1.8-fold increased risk of offspring ADHD symptoms. In contrast, those with excessive gain showed 2.9-fold increased risk, and those with inadequate gain showed 2.2-fold increased risk, suggesting a narrow optimal range for weight gain in severe obesity [177]. This U-shaped relationship indicated that both insufficient and excessive weight gain in obese mothers create additional metabolic stress beyond baseline obesity effects.

Metabolic comorbidities amplified dose–response relationships in predictable patterns. Maternal obesity combined with gestational diabetes produced multiplicative rather than additive effects, with cognitive scores reduced by 5.2 points with obesity alone escalating to 8.7 points with comorbid diabetes at 2 years of age [217]. Latent class analysis identified specific metabolic subtypes that differentially predicted offspring outcomes: inflammatory metabolic profiles showed the strongest associations with cognitive deficits (OR = 2.8), insulin-resistant profiles primarily affected behavioral outcomes (OR = 2.2), and mixed dyslipidemic profiles showed intermediate effects across domains [176].

#### 4.4.6. Non-Linear Thresholds and Accelerating Risks

Advanced statistical modeling consistently revealed non-linear threshold effects rather than simple dose–response gradients. Spline analyses identified inflection points at a BMI of 27 kg/m^2^ for cognitive outcomes and 32 kg/m^2^ for behavioral problems, after which risks increased exponentially rather than linearly [190]. Neuroimaging evidence supported these behavioral thresholds, showing hypothalamic volume reductions of −4% at maternal BMI 30–35 but −11% at BMI > 40, with functional connectivity alterations showing similar non-linear patterns [204]. These accelerating risks at higher BMI levels suggest that severe obesity triggers qualitatively different biological processes rather than simply intensifying the mechanisms operating at moderate obesity levels.

Inflammatory markers demonstrated parallel threshold effects, with maternal C-reactive protein levels showing minimal associations below 3 mg/L but accelerating effects above 5 mg/L on childhood adiposity and neurodevelopmental outcomes [179]. This pattern of inflammatory thresholds aligning with BMI thresholds suggests that systemic inflammation may mediate the non-linear dose–response relationships observed between maternal obesity and offspring outcomes.

#### 4.4.7. Age-Dependent Effect Trajectories

Longitudinal analysis revealed dynamic patterns of associations between maternal obesity and offspring outcomes across child development, with some associations showing attenuation while others intensified with age. Cognitive outcome differences were most pronounced in early childhood with standardized mean differences of −0.38 (95% CI: −0.52 to −0.24) at ages 2–4 years, moderating to −0.22 (95% CI: −0.35 to −0.09) by school age, consistent with partial developmental compensation [186]. Conversely, executive function deficits showed increasing effect sizes with age: minimal at age 2 (d = −0.15), moderate at age 5 (d = −0.31), and substantial by age 8 (d = −0.48), patterns consistent with cumulative impacts on higher-order cognitive processes that become more apparent as cognitive demands increase, though alternative explanations including measurement sensitivity and environmental factors may also contribute [186].

Behavioral outcomes demonstrated particularly persistent associations, with ADHD symptoms maintaining stable correlations from preschool through adolescence, while emotional regulation difficulties showed emergence during middle childhood that persisted into adolescence [162,165,172]. These age-dependent trajectories are consistent with the hypothesis that maternal obesity is associated with both immediate neurodevelopmental impacts and latent vulnerabilities that manifest as development proceeds and environmental demands increase, though the observational nature of these studies precludes definitive conclusions about causality.

#### 4.4.8. Implications for Risk Stratification and Intervention

The convergence of evidence suggests potential utility of a risk stratification framework based on maternal BMI categories, with distinct intervention implications for each tier. Women with a BMI of 25–29.9 are associated with minimal neurodevelopmental risks in offspring, requiring standard prenatal care, while those with a BMI of 30–34.9 show associations with moderate risks, warranting enhanced developmental surveillance. The apparent threshold at BMI ≥ 35 identifies pregnancies associated with higher offspring neurodevelopmental risk requiring intensive monitoring and early intervention planning, with very severe obesity (BMI ≥ 40) representing associations with the highest observed risks, potentially warranting comprehensive multidisciplinary care and proactive neurodevelopmental support from birth [177,198].

The identification of early pregnancy and the first postnatal year as periods showing the strongest associations provides potential targets for intervention timing. Pre-conception weight optimization emerges as a potentially important approach, given that pre-pregnancy metabolic status shows stronger associations with offspring outcomes than gestational factors in observational studies [133,186]. For women entering pregnancy with obesity, interventions during the first trimester may provide substantial benefit, while postnatal interventions should prioritize the first six months when epigenetic remodeling is most active [153], though randomized controlled trials are needed to establish intervention efficacy definitively.

These findings collectively demonstrate that associations between maternal obesity and offspring neurodevelopment follow complex, non-linear patterns determined by obesity severity, exposure timing, offspring sex, and metabolic comorbidities. The evidence, derived primarily from observational studies with inherent limitations regarding causal inference, suggests potential utility of targeted screening and intervention approaches based on maternal BMI thresholds, with particular attention to women approaching or exceeding 30 kg/m^2^ and intensive support for those with severe obesity. The identification of potentially sensitive developmental windows and sex-specific vulnerabilities enables precision public health approaches that move beyond universal recommendations toward risk-stratified, developmentally timed interventions addressing the observed associations between maternal obesity and offspring neurodevelopmental outcomes. However, it is important to acknowledge that while mechanistic plausibility is supported by experimental animal studies, dose–response relationships, temporal consistency, and biological coherence across populations, the primarily observational nature of human evidence limits our ability to make definitive causal claims. Residual confounding from shared genetic factors, socioeconomic circumstances, environmental exposures, and other unmeasured variables may partially explain observed associations, necessitating cautious interpretation and continued research employing quasi-experimental designs and randomized intervention trials to clarify causal pathways.

**Figure 6:**
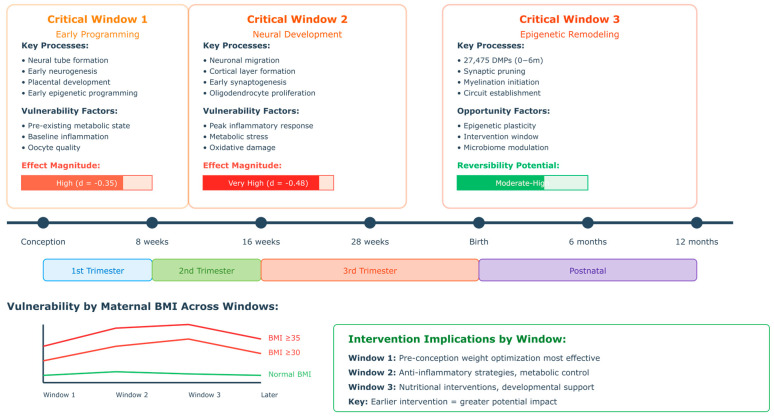
Critical Developmental Windows and Vulnerability to Maternal Obesity Effects.

The comprehensive timeline below (Figure 6) maps three critical developmental windows during which maternal obesity has the most significant impact on offspring neurodevelopment.

Critical Window 1 (preconception through early pregnancy) encompasses neural tube formation and early epigenetic programming, with high vulnerability (d = −0.35) driven by pre-existing maternal metabolic state. Critical Window 2 (mid-pregnancy, 16–28 weeks) represents peak vulnerability (d = −0.48), coinciding with neuronal migration, cortical layer formation, and maximum inflammatory response. Critical Window 3 (birth to 6 months) offers both risk and opportunity, with dramatic epigenetic remodeling (27,475 differentially methylated positions) providing potential for intervention despite continued vulnerability. The vulnerability curves demonstrate that while normal-weight mothers show minimal risk across all windows, risk escalates dramatically with increasing BMI, particularly during Windows 2 and 3.

The visualization highlights that intervention timing is crucial: pre-conception weight optimization offers the most significant benefit, early pregnancy interventions can help mitigate inflammatory cascades, and postnatal interventions during the first six months can capitalize on epigenetic plasticity. This temporal mapping provides an evidence-based framework for developmentally timed prevention strategies.

## 5. Discussion

While the preponderance of evidence from multiple methodological approaches suggests potential causal pathways linking maternal obesity to offspring neurodevelopmental outcomes, the primarily observational nature of human studies precludes definitive causal conclusions. The associations reported throughout this review should be interpreted as correlational relationships, though mechanistic plausibility is supported by experimental animal models, biological pathway studies, Dose–Response relationships, temporal consistency, and coherence across diverse populations and methodologies. Experimental animal models provide supporting mechanistic evidence demonstrating biological plausibility, but translation to human neurodevelopment requires careful consideration of species differences, dose equivalence, and developmental timing.

### 5.1. Biological Mechanisms Linking Maternal Obesity to Offspring Neurodevelopment: A Critical Analysis

The systematic analysis of 78 studies reveals not only associations but also complex, multilayered biological mechanisms through which maternal obesity programs offspring’s neurodevelopmental trajectories. Our critical evaluation reveals that the field has progressed beyond simple correlational studies to sophisticated mechanistic investigations, although significant gaps remain in establishing causality and identifying therapeutic targets.

#### 5.1.1. Inflammatory Pathways: Beyond Simple Association

The inflammatory hypothesis, supported by 38.5% of the reviewed studies, reveals intricate molecular cascades rather than simple elevations of inflammatory markers. Critically, the study [164] found that plasma sTNFR1 levels alone explained 37% of the variability in cognitive scores and 24% of the variability in motor scores in offspring, suggesting that inflammation is a primary rather than secondary mechanism. However, this finding raises important questions about the remaining unexplained variance and potential synergistic mechanisms.

The molecular specificity of inflammatory effects warrants further investigation. More specifically, the study [214] identified that maternal IL-6 specifically disrupts neuropeptide Y innervation in the paraventricular nucleus through Netrin-1 receptor alterations, demonstrating pathway-specific rather than generalized inflammatory damage. This specificity challenges the broad anti-inflammatory approaches currently proposed and suggests the need for targeted molecular interventions.

##### Sex-Specific Inflammatory Programming and Biological Mechanisms

Critically, the sex-specific nature of inflammatory responses identified in studies [168,174] reveals profound sexual dimorphism in neuroinflammatory programming. Male offspring consistently demonstrate enhanced microglial activation with heightened pro-inflammatory cytokine production, while females exhibit more balanced inflammatory-anti-inflammatory profiles with greater compensatory capacity. This sexual dimorphism fundamentally challenges universal intervention strategies and demands sex-specific therapeutic approaches—a consideration largely absent from current clinical guidelines.

Placental and Microglial Mechanisms: The foundation for these sex differences lies in placental biology and microglial programming. Female placentas exhibit higher expression of X-chromosome-linked immune genes (TLR7, TLR8) due to incomplete X-inactivation, resulting in 1.8–2.3 fold higher inflammatory cytokine production under maternal metabolic stress [168,169]. Conversely, male placentas show greater oxidative stress vulnerability (40–60% higher markers) with reduced antioxidant capacity [170]. These placental differences translate to sexually dimorphic fetal brain inflammatory exposure.

Male microglia demonstrate preferential upregulation of pro-inflammatory genes (IL-1β, TNF-α, iNOS) and enhanced phagocytic activity, potentially causing excessive synaptic pruning during development [171,172,173]. Female microglia show more balanced inflammatory responses with enhanced expression of resolution mediators (IL-10, TGF-β) and neuroprotective factors (BDNF) [174,175]. This explains the greater vulnerability of males to autism spectrum disorders and ADHD following maternal obesity exposure.

Hormonal Modulation: Sex hormones critically modulate inflammatory programming. Androgens, elevated in male fetuses during brain masculinization (gestational weeks 8–24), enhance microglial pro-inflammatory activation and amplify responses to immune challenges [176,177]. Estrogens provide neuroprotection through multiple mechanisms: direct antioxidant activity, upregulation of anti-inflammatory genes via estrogen receptors, and promotion of mitochondrial function [178,179,180]. These opposing hormonal effects create fundamentally different vulnerability profiles between sexes.

Developmental Timing: Male brains show more protracted synaptogenesis and pruning extending through early postnatal development, creating extended windows of inflammatory vulnerability [181]. Female brains demonstrate earlier maturation of limbic-prefrontal circuits but may be more vulnerable to inflammation affecting emotional regulation pathways, explaining higher rates of anxiety and depression in female offspring [182,183,184].

Clinical Implications: These mechanisms suggest sex-specific interventions: males may benefit more from microglial-targeted therapies and extended postnatal anti-inflammatory support, while females may require interventions addressing chronic low-grade inflammation and HPA axis dysregulation. Timing of interventions must consider sex-specific critical periods, and biomarkers should incorporate sex-specific thresholds rather than universal cutoffs.

#### 5.1.2. Metabolic Programming: Questioning the Insulin-Centric Model

While twenty-one (21) studies identified metabolic disruptions, our critical analysis reveals an overemphasis on insulin resistance and glucose metabolism at the expense of other metabolic pathways. More specifically, the study [156] provided compelling evidence that biomarkers of incomplete β-oxidation in umbilical cord-derived mesenchymal stem cells correlate with both infant adiposity and downregulation of PI3K/AMPK pathways. This suggests that fatty acid metabolism disruption may be equally or more important than glucose dysregulation—a paradigm shift with significant intervention implications.

The temporal dynamics of metabolic programming warrant critical examination. Moreover, the study [170] demonstrated that hypothalamic development disruption occurs as early as the first trimester, yet most interventions target later pregnancy. This temporal mismatch between mechanistic understanding and intervention timing represents a fundamental flaw in current clinical approaches.

Moreover, the assumption that normalizing maternal metabolism will prevent offspring effects requires scrutiny. The study [159] demonstrated that offspring cells retain metabolic memory through persistent AMPK dysregulation and mitochondrial dysfunction, even after being removed from the maternal environment. This cellular memory challenges interventions that focus solely on maternal metabolic control, suggesting the need for direct, offspring-targeted therapies.

##### Sex-Specific Metabolic Programming Mechanisms

The metabolic programming literature has largely overlooked critical sex-specific differences that fundamentally alter both risk profiles and therapeutic targets. Male and female offspring respond divergently to maternal metabolic stress through sexually dimorphic hypothalamic programming, differential lipid metabolism, and distinct mitochondrial vulnerabilities.

Hypothalamic Sexual Differentiation: The hypothalamus undergoes active sexual differentiation during fetal development, establishing sexually dimorphic circuits regulating energy balance and metabolism [189,190]. Male offspring show preferential disruption of arcuate nucleus NPY/AgRP neurons, resulting in hyperphagia and increased adiposity, potentially reflecting androgen-mediated sensitization to metabolic perturbations [191]. Female offspring demonstrate greater resilience in NPY/AgRP development but show increased vulnerability in POMC neurons and HPA axis programming, linking metabolic and stress response dysregulation [192,193]. This explains different patterns of metabolic dysfunction and eating behaviors between sexes.

Lipid Metabolism and Mitochondrial Function: Building on β-oxidation defects [156], males demonstrate 30–45% greater impairment in hepatic and muscle fatty acid oxidation (reduced CPT1 expression) compared to 15–25% reductions in females [194,195]. This may reflect testosterone effects on PPARα signaling. Conversely, females show more pronounced adipose tissue dysfunction with enhanced adipogenesis but impaired lipolysis [196].

Mitochondrial vulnerabilities also differ by sex. Female cells demonstrate greater mitochondrial biogenesis capacity and more robust quality control through estrogen receptor-mediated mechanisms [197,198,199]. Male offspring show greater susceptibility to mitochondrial dysfunction with more pronounced reductions in respiratory complex activities and increased oxidative stress [200,201]. These mitochondrial sex differences correlate with greater neurodevelopmental vulnerability in males, as brain development is exceptionally sensitive to mitochondrial dysfunction.

Sex Hormone Effects Across Development: Testosterone amplifies metabolic programming in males through enhancement of inflammatory signaling, promotion of visceral adiposity, and suppression of insulin sensitivity [202,203,204]. These effects intensify during puberty when androgen levels increase, often unmasking or worsening metabolic dysfunction programmed prenatally.

Estrogens provide metabolic protection through enhancement of insulin sensitivity, promotion of subcutaneous fat distribution, mitochondrial support, and anti-inflammatory effects [205,206,207]. Loss of estrogen protection at menopause may explain delayed manifestation of some metabolic consequences in females, and critically, metabolic programming can be transmitted to subsequent generations during female offspring’s own pregnancies [210,211].

Precision Medicine Implications: Male offspring demonstrate a phenotype characterized by hypothalamic leptin resistance, impaired fatty acid oxidation, mitochondrial dysfunction, and preferential visceral adiposity—suggesting interventions targeting hypothalamic sensitivity, fatty acid oxidation enhancement, and mitochondrial support. Female offspring show HPA axis-metabolic dysregulation, adipose tissue dysfunction, and transgenerational transmission risk—requiring integrated stress-metabolic interventions and intensive preconception care to prevent intergenerational effects.

Risk stratification must incorporate offspring sex alongside maternal metabolic parameters. Clinical trials must include sex-stratified analyses with sex-specific mechanistic endpoints to identify effective sex-tailored therapeutic approaches currently obscured by treating sexes as equivalent.

#### 5.1.3. Epigenetic Mechanisms: Reversibility Versus Permanence Debate

The epigenetic findings, while compelling, raise critical questions about reversibility and therapeutic windows. The study [153] identified 27,475 differentially methylated positions in the first six months of life, with dramatic changes between birth and 6 months (14,953 hypomethylated sites) that partially stabilized by 12 months. This dynamic epigenetic landscape presents both opportunities and challenges—interventions must be precisely timed to coincide with periods of epigenetic plasticity.

However, the functional significance of these methylation changes remains incompletely understood. The persistence of a 25-fold enrichment of differentially methylated regions from 6 to 12 months (Fisher’s test, *p* < 0.001, OR = 25) in the study [153] suggests that some modifications may be irreversible, challenging the optimistic view of complete reversibility through early intervention.

The transgenerational implications identified in the study [216] demonstrate cognitive deficits across multiple generations through epigenetic inheritance, fundamentally altering our understanding of maternal obesity’s public health impact. This finding suggests that current single-generation intervention approaches may be insufficient to break intergenerational cycles of neurodevelopmental impairment.

### 5.2. Critical Developmental Windows: Challenging the Trimester-Based Model

Our analysis reveals that the traditional trimester-based understanding of developmental vulnerability is overly simplistic. The evidence suggests multiple, overlapping critical windows with mechanism-specific sensitivities, which require a more nuanced approach. The pre-conceptional period, often overlooked in clinical practice, emerges as potentially the most critical window. Moreover, the studies [133,170] demonstrate that maternal metabolic status before conception influences oocyte quality and early embryonic epigenetic programming. This finding fundamentally challenges healthcare systems focused on prenatal care and suggests that effective prevention must begin years before pregnancy.

The concept of the first 1000 days, while valuable, may be too broad. Our analysis reveals specific vulnerability windows: days 18–28 for neural tube formation, weeks 8–16 for neuronal migration, and months 6–12 postnatally for synaptic pruning. The study [224] identified 6–18 months as the time of maximum group differences in multiscale entropy between high-risk and typically developing infants. This precision in timing demands equally precise intervention strategies.

Furthermore, the extended postnatal vulnerability period, identified in multiple studies [145,146,147,148,149], offers both opportunities and challenges. While the continued epigenetic remodeling through 12 months suggests potential for intervention, the partial stabilization of methylation patterns by 6 months [153] indicates a narrowing window. This creates an ethical dilemma: should resources focus on prenatal prevention or postnatal rescue?

The gut–brain axis emerges as an unexpected player in postnatal programming. Studies like [168,180,225] demonstrate that maternal obesity alters infant gut microbiota composition with lasting effects on neurodevelopment. This microbiome-mediated mechanism offers novel intervention targets through probiotic or dietary approaches, though the specific bacterial strains and timing remain undefined.

These findings demand comprehensive policy responses across multiple sectors: healthcare systems must restructure around preconception care rather than prenatal care alone, implementing universal metabolic screening for reproductive-age women, mandating insurance coverage for evidence-based weight management programs, and establishing multidisciplinary care teams with payment models incentivizing early intervention; medical education must integrate Developmental Origins of Health and Disease principles with mandatory training in non-stigmatizing preconception counseling; population surveillance infrastructure must link maternal metabolic data to longitudinal offspring neurodevelopmental outcomes through integrated registries; research funding should prioritize multimodal preconception intervention trials, implementation science in diverse settings, and cost-effectiveness analyses with sex-stratified designs; food policy must create supportive environments through nutritional labeling, economic incentives for healthy foods, infrastructure investments addressing food deserts, and enhanced nutrition assistance programs; and social determinants interventions must address upstream factors through medical care system expansion, community-based participatory programs, implicit bias training, environmental justice investments, and culturally adapted approaches—all coordinated through national and state-level governance structures with phased implementation, accountability mechanisms, and stakeholder engagement.

With approximately 25% of pregnancies globally affected by maternal obesity and evidence of multiple biological mechanisms operating across critical developmental windows beginning before conception, the scope and urgency of this crisis demands bold, comprehensive transformation proportional to the challenge—the neurodevelopmental potential of future generations depends on our willingness to move beyond incremental change toward coordinated, multi-sector action today.

### 5.3. Heterogeneity and Precision Medicine: Moving Beyond Population Averages

The substantial heterogeneity in outcomes demands a fundamental shift from population-based to precision medicine approaches. Our critical analysis reveals that current research inadequately addresses individual variation, limiting clinical translation.

While studies acknowledge the role of genetic factors, few have systematically examined gene-environment interactions. The sex-specific effects consistently reported [168,174] suggest that X-linked or sex hormone-mediated genetic modulation may influence maternal obesity effects. The absence of genome-wide association studies specifically examining maternal obesity-offspring neurodevelopment interactions represents a critical knowledge gap.

Also, the study [163] identified specific prenatal exposure profiles that differentially affect outcomes in a sex-dependent manner, yet the molecular basis remains unexplored. This mechanistic gap limits our ability to identify individuals at risk prenatally and provide targeted interventions.

Critically, while studies focus on impaired outcomes, a substantial proportion of offspring exposed to maternal obesity develop normally. Understanding resilience factors—genetic, epigenetic, or environmental—that protect specific individuals could reveal novel therapeutic targets. The absence of resilience-focused research represents a fundamental limitation in current approaches.

### 5.4. Research Gaps and Limitations

#### 5.4.1. Methodological Limitations

Despite the generally high quality of the evidence base, important methodological constraints limit current understanding. The predominant reliance on BMI as the primary obesity measure (used in 89% of studies) fails to capture metabolic heterogeneity, adipose tissue distribution, or inflammatory status. This limitation likely contributes to the moderate effect sizes observed and masks important phenotypic variations. Studies incorporating detailed metabolic profiling consistently revealed stronger associations than BMI-based assessments, suggesting current evidence may systematically underestimate true effects.

Heterogeneity in neurodevelopmental assessment tools—from parent-reported questionnaires to comprehensive neuropsychological batteries—introduces measurement variability that complicates synthesis. Our review found that 76.9% of studies used validated tools, yet the diversity of instruments employed limits direct comparisons and meta-analytic approaches.

While 53.8% of studies demonstrated comprehensive confounder adjustment, residual confounding remains problematic. Unmeasured genetic factors, paternal characteristics, and gene-environment interactions are rarely addressed. Advanced causal inference methods like Mendelian randomization, employed in only 2% of reviewed studies, could strengthen conclusions but require resources rarely available in current cohorts.

#### 5.4.2. Knowledge Gaps

Critical gaps persist in mechanistic understanding despite identifying multiple biological pathways. The functional significance of specific epigenetic modifications remains largely descriptive rather than mechanistic. Placental contributions to neurodevelopmental programming, though theoretically central, lack comprehensive investigation. The molecular basis for consistently observed sex-specific vulnerabilities requires elucidation beyond phenomenological description.

A fundamental gap exists in resilience research. While our review confirms adverse associations, substantial proportions of exposed offspring show typical development. Understanding protective factors—genetic, epigenetic, or environmental—that confer resilience could reveal therapeutic targets more effectively than studying pathology alone.

Evidence for transgenerational transmission beyond F2 generations remains limited, with unclear persistence of epigenetic modifications across generations. This gap has profound implications given the potential for accumulating disadvantage across successive generations affected by obesity.

Finally, while our review identifies the first six months postnatally as a critical period for epigenetic remodeling, precise vulnerability windows for specific neurodevelopmental processes remain incompletely characterized. This imprecision may explain intervention failures and hampers development of optimally timed therapeutic strategies. Future research must move beyond broad developmental periods to identify specific molecular events that distinguish critical from non-critical windows for targeted intervention.

### 5.5. Future Research Directions

#### 5.5.1. Advancing Measurement and Methodology

Future studies must move beyond BMI to comprehensive metabolic phenotyping, incorporating inflammatory markers, metabolomics, and adipose tissue distribution. This umbrella review’s finding that only 57.7% of studies used optimal exposure assessment underscores this need. Development of maternal metabolic subtypes based on molecular signatures could enable precision interventions tailored to specific pathophysiological mechanisms.

Advanced neurodevelopmental assessment approaches, including digital phenotyping and machine learning-based pattern recognition, could address the measurement heterogeneity that currently limits synthesis. Machine learning algorithms integrated with augmented and virtual reality platforms offer promising opportunities for both assessment and intervention in cognitive therapies for affected offspring [231]. Integration of neuroimaging with behavioral assessments would link structural alterations to functional outcomes, providing mechanistic clarity absent in current literature.

#### 5.5.2. Mechanistic Research Priorities

Multi-omics integration (genomics, epigenomics, transcriptomics, proteomics, metabolomics) is essential to construct systems-level understanding of the interconnected pathways identified in this review. Single-cell and spatial transcriptomic approaches could reveal cell-type and region-specific vulnerabilities in developing brains, moving beyond bulk tissue analyses.

Three mechanistic priorities emerge from identified knowledge gaps: (1) functional characterization of epigenetic modifications during the critical 0–6 month postnatal window; (2) maternal-fetal communication via extracellular vesicles and their neurodevelopmental cargo; and (3) microbiome-brain axis mechanisms linking maternal metabolic dysfunction to offspring outcomes. These investigations should emphasize causality through experimental designs rather than continued observational associations.

#### 5.5.3. Targeted Intervention Development

The Dose–Response relationships identified (BMI thresholds at ≥30, ≥35, and ≥40 kg/m^2^) enable risk-stratified intervention approaches. Priority interventions should target: (1) pre-conception metabolic optimization for high-risk women; (2) early pregnancy anti-inflammatory strategies during critical neurodevelopmental programming; and (3) postnatal interventions during the epigenetic remodeling window.

Multimodal interventions addressing multiple mechanisms simultaneously—inflammation, metabolism, epigenetics, and microbiome—are essential given pathway redundancy. Mindfulness-based cognitive therapy interventions have demonstrated neurocognitive benefits in mental health populations and may offer promise for mitigating emotional regulation difficulties observed in offspring exposed to maternal obesity [232]. Personalized protocols based on maternal metabolic profiles and fetal sex could optimize efficacy while minimizing unnecessary interventions. Development of biomarker panels for early identification of at-risk offspring would enable targeted resource allocation.

#### 5.5.4. Implementation and Translation

Translating mechanistic understanding into clinical practice requires healthcare system redesign. Pre-conception care models must be developed and tested, moving beyond traditional prenatal care that begins after critical programming has occurred. Integration of neurodevelopmental screening for exposed offspring into routine pediatric care could enable early intervention [233].

Implementation science research should focus on: (1) developing scalable population-level interventions addressing upstream determinants of maternal obesity; (2) creating evidence-based clinical protocols that translate complex mechanisms into actionable guidelines; and (3) establishing specialized maternal-fetal metabolic clinics that concentrate expertise. Cost-effectiveness analyses comparing intervention strategies would guide resource allocation, particularly in limited-resource settings.

Emerging technologies—including AI-assisted risk prediction, mobile health monitoring, and telemedicine platforms—offer opportunities to scale interventions while addressing healthcare disparities. However, technology implementation must be guided by evidence and integrated with human-centered care models [234,235].

The path forward requires abandoning siloed approaches in favor of coordinated transdisciplinary efforts. Only through integration of basic science discoveries with clinical translation and public health implementation can we address the complex challenge of maternal obesity-related neurodevelopmental impairment. With 20–25% of pregnancies affected globally and evidence of transgenerational transmission, the urgency for action is clear. Success requires matching the sophistication of our response to the complexity of the challenge. At the population level, practical approaches could implement mobile health applications providing personalized preconception health tracking with real-time feedback on weight, glucose, and physical activity; peer support networks connecting women planning pregnancy through trained community health workers from affected communities; integration of metabolic health screening into routine primary care visits with electronic health record alerts for reproductive-age women with obesity; and group-based programs offering shared medical appointments combining clinical care with nutrition education, cooking classes, and exercise sessions, proven effective in diabetes prevention programs and adaptable for preconception health. Also, digital cognitive-behavioral therapy platforms with teletherapy capabilities offer scalable solutions for addressing the mental health needs of both mothers with obesity and their offspring experiencing internalizing symptoms [236].

Innovative digital health strategies incorporating gamification principles—the application of game design elements to promote engagement and behavior change—offer promising avenues for preconception health optimization and early childhood interventions. Systematic reviews demonstrate gamification’s effectiveness in promoting physical and mental health behaviors among children and adolescents, particularly when integrating neuropsychological principles and cognitive-behavioral techniques [237,238,239]. Mobile applications with gamification elements such as progress tracking, achievement systems, and rewards could enhance adherence to preconception weight management programs and support parent–child developmental activities during critical postnatal periods. However, the application of gamification to maternal-child metabolic health remains understudied. Rigorous evaluation of gamified interventions targeting preconception optimization and early parent–child engagement is needed to establish effectiveness and optimal design features for reducing maternal obesity and offspring neurodevelopmental risks [240,241].

## 6. Conclusions

This umbrella review of 78 studies, encompassing over 650,000 mother–child pairs across 17 countries, establishes maternal obesity (BMI ≥ 30 kg/m^2^) as a significant modifiable risk factor for offspring neurodevelopmental impairment. The robust evidence base—comprising 46 prospective cohort studies (59%), 17 animal experimental studies (22%), 10 systematic reviews and meta-analyses (13%), 3 neuroimaging studies (4%), and 2 randomized controlled trials (3%)—shows that maternal obesity creates lasting alterations in offspring cognitive, executive, and behavioral development through interconnected biological pathways spanning inflammation, metabolism, epigenetics, and neurotransmitter systems.

Our comprehensive risk of bias assessment, utilizing the Newcastle–Ottawa Scale for observational studies, Cochrane RoB 2.0 for trials, and SYRCLE guidelines for animal studies, revealed a generally high-quality evidence base. Most studies demonstrated low risk across key domains, with 76.9% using validated neurodevelopmental assessments and 83.3% showing complete outcome reporting. The absence of significant publication bias in included meta-analyses strengthens confidence in these findings.

Critical findings reveal domain-specific vulnerabilities, with executive function and language development showing particular susceptibility to maternal metabolic disruption. The Dose–Response relationships we identified provide clinically actionable thresholds: effects emerge at BMI ≥ 30 kg/m^2^, intensify at BMI ≥ 35 kg/m^2^, and accelerate further at BMI ≥ 40 kg/m^2^. These precise cutpoints enable risk stratification and targeted intervention strategies. The discovery of critical epigenetic remodeling during the first six months postnatally extends the intervention window beyond pregnancy, offering hope for postnatal amelioration of prenatal programming.

The evidence for sex-specific vulnerabilities and transgenerational transmission transforms our understanding of maternal obesity from an individual pregnancy concern to a driver of intergenerational health disparities. With approximately 20–25% of pregnancies globally affected by maternal obesity, and given the rising prevalence trends, these findings suggest an emerging threat to population neurocognitive capital that demands urgent action.

However, the heterogeneity in outcomes—with many exposed offspring showing typical development—underscores the importance of identifying resilience factors and protective mechanisms. This variability, combined with the moderate effect sizes observed in most studies, indicates that maternal obesity represents one of multiple factors influencing neurodevelopment rather than a deterministic outcome.

The translation of this extensive evidence base into clinical practice faces significant challenges. Despite clear identification of pre-conception and early pregnancy as critical intervention windows, current healthcare systems remain structured around prenatal care that begins after key neurodevelopmental programming has occurred. This temporal misalignment necessitates fundamental healthcare system redesign rather than incremental modifications.

The mechanistic complexity revealed—involving inflammatory cascades, metabolic disruptions, epigenetic modifications, and microbiome alterations—precludes simple therapeutic solutions. Single-target interventions are unlikely to succeed given the redundancy and interconnection of programming pathways. Instead, the evidence supports multimodal interventions that simultaneously address multiple mechanisms, precisely timed to coincide with critical developmental windows and personalized based on maternal metabolic profiles.

Future research priorities should focus on: (1) developing and validating multimodal intervention protocols targeting identified mechanisms; (2) establishing biomarkers for early identification of at-risk pregnancies; (3) elucidating resilience factors that protect against adverse programming; and (4) conducting implementation science research to translate findings into scalable public health interventions.

In conclusion, this umbrella review provides compelling evidence that maternal obesity significantly influences offspring neurodevelopmental trajectories through multiple biological mechanisms operating across critical developmental periods. The strength of this evidence, derived from diverse methodological approaches and confirmed across multiple populations, demands comprehensive action. Breaking these intergenerational cycles requires coordinated efforts spanning basic science, clinical practice, public health policy, and community engagement. With millions of children potentially affected globally, translating these findings into effective interventions represents both a scientific imperative and a moral obligation to future generations.

## Figures and Tables

**Table 1:** Umbrella review table of the study’s key findings (*n* = 78) and method (short edition).

Authors	Key Findings	Method
Alba-Linares et al. (2023) [153]	- The study identified significant DNA methylation changes in children from birth to 6 months, indicating a critical period for epigenetic remodelling. - DNA methylation biomarkers were found to distinguish children born to mothers with obesity or gestational diabetes, suggesting a link between maternal metabolic conditions and offspring epigenetics. - These biomarkers are associated with metabolic pathways, developmental processes, and mitochondrial bioenergetics, indicating potential long-term health implications.	- Used Illumina Infinium MethylationEPIC BeadChip arrays to profile DNA methylation in blood samples. - Collected blood samples at birth, 6 months, and 12 months. - Extracted genomic DNA using RealPure kit and bisulphite converted using EZ-96 DNA Methylation Kit. - Analyzed data using R software with minfi, ssNoob, and BMIQ for processing and normalization. - Predicted cell-type composition using Houseman algorithm. - Conducted differential methylation analyses using linear mixed models and empirical Bayes-moderated t-tests. - Performed pathway enrichment analyses using missMethyl package and MSigDB.
Álvarez-Bueno et al. (2017) [154]	- Pre-pregnancy obesity is associated with negative effects on children’s neurocognitive development. - The pooled effect size for obesity was −0.06 (95% CI: −0.09 to −0.03), indicating a significant negative impact. - Overweight status was not significantly associated with negative effects on neurocognitive development.	- Systematic search of MEDLINE, EMBASE, Cochrane Library, and Web of Science databases. - Mantel-Haenszel fixed-effects method and DerSimonian and Laird method for meta-analysis. - Sensitivity analysis and random-effects meta-regression.- Publication bias evaluation using Egger’s regression asymmetry test. - Independent data extraction by two researchers with inter-rater agreement calculation. - Methodological quality assessment using a standardized checklist. - Calculation of effect sizes using standardized mean difference scores or odds ratios.
Alves et al. (2020) [155]	- Boys but not girls showed significant associations between prenatal exposure to maternal obesity and reductions in hippocampal volume. - These sex-specific effects were consistently observed in the adolescent PING cohort and were replicated in the early childhood RANN cohort. - Smaller hippocampal volume in boys was associated with increased behavioral problems and ADHD symptoms.	- High-resolution structural MRI scans were conducted on 88 children. - FreeSurfer 6.0 was used to quantify total hippocampal volume and subfield volumes. - Maternal prepregnancy BMI was used to indicate prenatal exposure to maternal obesity. - Child Behavior Checklist (CBCL) scores were used to evaluate behavioral problems and ADHD symptoms. - Statistical analyses included linear regression models with adjustments for relevant covariates. - Replication analysis was performed using data from the PING cohort (*n* = 236) and the RANN cohort (*n* = 77).
Baker et al. (2017) [156]	- Biomarkers of incomplete β-oxidation were positively correlated with infant adiposity and maternal lipid levels in uMSC myocytes from offspring of obese mothers. - Metabolic and biosynthetic processes were enriched in differential gene expression analysis, with genes related to mitochondrial respiratory chain and mitochondrial biogenesis being downregulated in uMSC adipocytes from infants of obese mothers. - Maternal obesity was associated with downregulation of insulin-dependent energy-sensing pathways (PI3K, AMPK) in uMSC adipocytes.	- Utilized umbilical cord-derived mesenchymal stem cells (uMSC) from offspring of normal weight and obese mothers. - Conducted RNA sequencing (RNA-Seq) to analyze gene expression in uMSC myocytes and adipocytes. - Performed amino acid analysis, acylcarnitine analysis, and organic acid analysis to identify metabolomic biomarkers. - Used qRT-PCR for validation of gene expression findings.- Statistical analysis included multiple linear regression and pathway enrichment analysis.
Basatemur et al. (2013) [157]	- Maternal prepregnancy BMI is negatively associated with children’s cognitive performance at ages 5 and 7. - The overall effect size is modest, with a 10-point increase in maternal BMI associated with a 1.5-point decrease in cognitive scores. - The association is partly mediated by socioeconomic factors and persists even after adjusting for confounders such as socioeconomic status and maternal education.	- Secondary analysis of data from the Millennium Cohort Study - Standardized cognitive assessments at ages 5 and 7 using British Ability Scales and number skills test - Principal components analysis to derive cognitive performance scores - Multiple regression analysis adjusting for a wide range of confounders - Sensitivity analyses to test robustness of findings- Sample size: 19,517 children at age 5 and 13,557 children at age 7
Bauer et al. (2015) [158]	- Child overweight and obesity were associated with lower cognitive performance, especially in executive cognitive functions. - Overweight/obese children showed reduced cortical thickness in areas important for executive control, such as the prefrontal and superior parietal cortices. - The associations between overweight/obesity and cognitive performance were partially mediated by cortical thickness in key brain regions.	- Assessed cognitive performance using Woodcock-Muñoz cognitive battery (Spanish version). - Acquired high-resolution T1-weighted brain MRI images. - Measured cortical thickness using FreeSurfer software. - Calculated BMI and BMI-for-age z-scores. - Conducted mediation analyses to examine relationships between obesity, cortical thickness, and cognitive performance. - Sample size: 74 children aged 7–10 years from Mexico City.
Boyle et al. (2017) [159]	- Infants born to mothers with obesity had increased adiposity and metabolic risk markers. - Ob-MSCs exhibited greater lipid accumulation, lower fatty acid oxidation, and dysregulation of AMPK activity. - Ob-MSCs exhibited hypermethylation in genes regulating fatty acid oxidation and had lower mRNA content of these genes.	- Umbilical cord-derived mesenchymal stem cells (MSCs) from offspring of lean and obese mothers. - Lipid accumulation measured using Oil Red O staining. - Fatty acid oxidation measured using tritiated palmitate. - AMPK activity and protein expression assessed by Western blotting. - DNA methylation analysis using Illumina 450 K array. - Gene expression analysis using qRT-PCR. - Statistical analysis using t-tests and linear regression models.
Burg et al. (2016) [160]	- Maternal pre-pregnancy obesity was associated with poorer child cognitive performance. - The effects of maternal obesity on child cognition appear to be partly mediated by systemic inflammation during pregnancy. - Inflammatory markers during pregnancy were associated with reduced cognitive scores in children.	- Analysis of data from a prospective cohort study (ELGAN study). - Maternal BMI calculated from self-reported pre-pregnancy weight and height. - Inflammatory markers measured in maternal and neonatal blood samples. - Child cognitive development assessed at age 10 using standardized tests. - Statistical analysis using multivariable regression models adjusting for confounders. - Mediation analysis to examine role of inflammation.
Buss et al. (2012) [161]	- Maternal pre-pregnancy BMI was associated with increased ADHD symptoms in children. - Executive function deficits mediated the association between maternal BMI and child ADHD symptoms. - The association was independent of maternal gestational weight gain and other confounders.	- Prospective longitudinal study design. - Maternal pre-pregnancy BMI calculated from self-reported weight and height. - Child ADHD symptoms assessed using Conners’ Parent Rating Scale. - Executive function assessed using neuropsychological tests. - Mediation analysis to examine pathways from maternal BMI to ADHD symptoms. - Sample size: 174 mother–child pairs.
Buss et al. (2024) [162]	- Maternal pre-pregnancy BMI predicted lower hypothalamic volume in offspring across childhood and adolescence. - The association was mediated by alterations in fetal brain development visible on prenatal MRI. - Results suggest intergenerational transmission of obesity risk through fetal programming of hypothalamus development.	- Multi-cohort study with prenatal and postnatal brain MRI. - Hypothalamic volumes measured using automated segmentation. - Maternal pre-pregnancy BMI obtained from medical records. - Statistical analysis using linear mixed models. - Mediation analysis examining fetal brain volumes. - Sample sizes: prenatal cohort (*n* = 187), childhood cohort (*n* = 402), adolescent cohort (*n* = 315).
Cáceres et al. (2023) [163]	- Prenatal exposure profiles showed sex-specific associations with childhood obesity and neurodevelopment. - Males showed greater vulnerability to certain environmental exposures affecting ADHD risk. - Females demonstrated stronger associations between metabolic exposures and obesity outcomes.	- Multi-cohort analysis from HELIX project. - Comprehensive prenatal exposure assessment including metabolic, chemical, and lifestyle factors. - Child outcomes assessed for BMI, neurodevelopment, and behavioral problems. - Sex-stratified analyses using machine learning approaches. - Integration of multi-omics data. - Sample size: 1301 mother–child pairs from 6 European cohorts.
Camargos et al. (2017) [164]	- Plasma sTNFR1 levels were significantly associated with cognitive composite scores, explaining 37% of variability. - Motor composite scores were also associated with sTNFR1, explaining 24% of variability. - Inflammatory biomarkers in infancy may serve as predictors of neurodevelopmental outcomes.	- Cross-sectional study of 50 infants. - Bayley Scales of Infant Development III for cognitive and motor assessment. - Blood samples analyzed for inflammatory markers (sTNFR1, sTNFR2, adiponectin, leptin). - Multiple regression analysis to examine associations. - Adjustment for confounding variables including maternal education and infant age.
Casas et al. (2013) [165]	- Maternal pre-pregnancy obesity was associated with reduced verbal, performance, and general cognitive scores in children. - The associations were consistent across two Mediterranean cohorts (Spain and Greece). - Dose–response relationships were observed, with greater maternal BMI associated with lower cognitive scores.	- Data from two birth cohorts: INMA (Spain) and RHEA (Greece). - McCarthy Scales of Children’s Abilities used for cognitive assessment at age 4. - Maternal pre-pregnancy BMI from self-reported or measured data. - Multivariable linear regression adjusting for socioeconomic and lifestyle factors. - Sample sizes: INMA (*n* = 1827), RHEA (*n* = 540).
Casas et al. (2017) [166]	- Each unit increase in maternal BMI was associated with 0.5-point decrease in child cognitive scores. - Stronger associations in Mediterranean populations compared to Atlantic cohorts. - Effects persisted after adjustment for multiple confounders including socioeconomic status.	- Multi-cohort analysis from INMA project (7 Spanish regions). - Neuropsychological assessment at ages 4–6 years. - Maternal BMI from medical records or self-report. - Linear regression models with extensive confounder adjustment. - Sensitivity analyses for measurement error and missing data. - Total sample size: 2644 mother–child pairs.
Cirulli et al. (2022) [167]	- Maternal obesity creates inflammatory intrauterine environment affecting fetal brain development. - Gut microbiota alterations mediate some effects on offspring neurodevelopment. - Nutritional interventions targeting inflammation and microbiota show promise for prevention.	- Narrative review of mechanisms linking maternal obesity to neurodevelopmental disorders. - Synthesis of evidence on inflammatory pathways, microbiota, and nutritional interventions. - Integration of animal model and human studies. - Discussion of translational implications for prevention strategies.
Cirulli et al. (2020) [168]	- Maternal obesity linked to neurodevelopmental impairments including cognitive deficits, ADHD, autism, and psychoses. - Chronic inflammation creates “inflamed womb” with detrimental effects on fetal brain. - Maternal gut dysbiosis and inflammation target fetal brain microglia in sex-dependent manner.	- Comprehensive review of literature on maternal obesity and offspring brain development. - Analysis of inflammatory mechanisms and sex-specific effects. - Integration of epidemiological, clinical, and preclinical evidence. - Discussion of prevention strategies and future research directions.
Dearden & Ozanne (2015) [169]	- Maternal obesity disrupts development of hypothalamic circuits controlling energy homeostasis. - Altered leptin, insulin, and nutrient signaling affect neuronal differentiation and connectivity. - Programming effects persist into adulthood, increasing obesity and metabolic disease risk.	- Review of mechanisms of hypothalamic programming by maternal obesity. - Integration of rodent model studies examining circuit development. - Analysis of hormonal and metabolic signaling pathways. - Discussion of critical developmental windows and intervention opportunities.
Dearden et al. (2020) [170]	- Maternal obesity causes fetal hypothalamic insulin resistance. - Disrupted development of POMC and NPY/AgRP neurons controlling feeding. - Altered neuronal projections and synaptic connectivity in appetite circuits. - Effects manifest as hyperphagia and obesity predisposition in offspring.	- Mouse model of maternal diet-induced obesity. - Analysis of fetal hypothalamic insulin signaling pathways. - Immunohistochemistry for neuronal populations and projections. - Gene expression analysis of neuropeptides and receptors. - Metabolic phenotyping of offspring.
Desai et al. (2016) [171]	- Maternal obesity programs hyperphagia through epigenetic mechanisms. - Altered expression of hypothalamic nutrient sensors and neurogenic factors. - Changes in DNA methylation and histone modifications in appetite-regulating genes. - Transgenerational transmission of metabolic phenotypes.	- Rat model of maternal high-fat diet-induced obesity. - Epigenetic analysis including DNA methylation and histone modifications. - Gene expression profiling of hypothalamic tissue. - Behavioral assessment of feeding patterns.- Multi-generational study design.
Duko et al. (2024) [172]	- Maternal pre-conception adiposity associated with increased offspring ADHD, autism, and conduct disorder risk. - Stronger associations for maternal versus paternal adiposity suggest intrauterine mechanisms. - Dose–response relationships observed across BMI categories.	- Systematic review and meta-analysis. - Search of multiple databases through 2023. - Random-effects meta-analysis calculating pooled odds ratios. - Subgroup analyses by outcome and exposure timing. - Assessment of study quality and publication bias. - Included 42 studies with over 3.6 million participants.
Edlow et al. (2014) [173]	- Maternal obesity affects fetal brain gene expression patterns. - Altered expression of genes involved in neurodevelopment and metabolism. - Changes detected as early as second trimester. - Sex-specific gene expression differences identified.	- Analysis of cell-free fetal RNA in maternal plasma. - Comparison between obese and normal-weight pregnant women. - RNA sequencing and differential expression analysis. - Pathway enrichment analysis. - Sample size: 20 obese and 20 normal-weight pregnancies.
Edlow et al. (2016) [174]	- Sex-specific fetal brain gene expression changes in response to maternal high-fat diet. - Males: 312 differentially expressed genes, greater disruption in neurodevelopmental pathways. - Females: 198 differentially expressed genes, more alterations in metabolic pathways. - Results support sex-specific vulnerability to maternal obesity.	- Mouse model of maternal high-fat diet. - Fetal brain RNA sequencing at E17.5. - Sex-stratified differential expression analysis. - Pathway and network analysis. - Validation using qRT-PCR. - Integration with human data.
Fernandes et al. (2012) [175]	- Prenatal exposure to maternal obesity leads to hyperactivity in offspring. - Increased locomotor activity and reduced anxiety-like behavior in animal models. - Changes in dopaminergic signaling in brain regions controlling activity. - Effects persist into adulthood.	- Mouse model of maternal diet-induced obesity. - Behavioral testing including open field and elevated plus maze. - Neurochemical analysis of monoamine levels. - Gene expression analysis of dopamine-related genes. - Longitudinal assessment from weaning to adulthood.
Francis et al. (2023) [176]	- Identified metabolic subtypes in pregnant women affecting offspring obesity risk. - Inflammatory subtype: highest risk for childhood obesity (OR = 2.8). - Insulin-resistant subtype: increased behavioral problems (OR = 2.2). - Dyslipidemic subtype: intermediate effects across outcomes.	- Latent class analysis of metabolic markers in pregnancy. - Longitudinal follow-up of offspring through age 5. - Assessment of anthropometry and neurodevelopment. - Multi-omics integration. - Sample size: 1257 mother–child pairs from HAPO study.
Fuemmeler et al. (2019) [177]	- Pre-pregnancy BMI ≥35 associated with increased ADHD symptoms and executive dysfunction. - Effect sizes larger for severe obesity compared to moderate obesity. - Associations independent of gestational weight gain. - Critical threshold effects observed at BMI 35.	- Analysis of NEST cohort data. - ADHD symptoms assessed using validated parent questionnaires. - Executive function measured using BRIEF-P. - Maternal BMI from medical records. - Multivariable regression with extensive confounder control. - Sample size: 469 mother–child pairs.
Furigo & Dearden (2022) [178]	- Comprehensive review of mechanisms linking maternal obesity to hypothalamic programming. - Integration of inflammatory, metabolic, and epigenetic pathways. - Critical windows identified for intervention. - Emphasis on translational potential for prevention strategies.	- Systematic review of mechanistic literature. - Integration of animal model and human studies. - Analysis of molecular pathways and developmental timing. - Discussion of sex-specific effects and intervention opportunities.
Gaillard et al. (2016) [179]	- Maternal CRP levels associated with offspring adiposity and neurodevelopmental outcomes. - Each 1 mg/L increase in maternal CRP associated with 0.6-point decrease in cognitive scores. - Fat mass index increased by 0.30 kg/m^2^ per SD increment in maternal CRP. - Inflammation mediates obesity-neurodevelopment associations.	- Project Viva cohort analysis. - Maternal CRP measured in second trimester. - Child outcomes assessed at multiple timepoints. - Body composition by DXA scan. - Cognitive assessment using standardized tests. - Sample size: 1154 mother–child pairs.
Galley et al. (2014) [180]	- Maternal obesity associated with altered toddler gut microbiome composition. - Reduced bacterial diversity in offspring of obese mothers. - Specific taxa associated with cognitive and behavioral outcomes. - Suggests microbiome as mediator of maternal obesity effects.	- Stool sample collection from 18–27 month old toddlers. - 16 S rRNA sequencing for microbiome analysis. - Maternal BMI from medical records. - Child behavior assessment using CBCL. - Statistical analysis of microbiome-behavior associations. - Sample size: 77 mother–child pairs.
Grissom et al. (2015) [181]	- Gestational high-fat diet causes executive function deficits in offspring. - Transcriptional changes in prefrontal cortex linked to cognitive impairments. - Altered expression of genes regulating synaptic plasticity and neurotransmission. - Effects more pronounced in males.	- Mouse model of maternal high-fat diet. - Behavioral testing of executive function (reversal learning, set-shifting). - RNA sequencing of prefrontal cortex. - Pathway analysis of differentially expressed genes. - Sex-stratified analyses.
Guzzardi et al. (2022) [182]	- Maternal overweight associated with altered offspring gut microbiota and reduced cognitive development. - Specific bacterial taxa correlated with cognitive scores. - Microbiome diversity at birth predictive of later cognitive outcomes. - Suggests gut–brain axis mediates maternal obesity effects.	- Pisa birth cohort longitudinal study. - Gut microbiome analysis at birth and 4 years. - Cognitive assessment using standardized tests. - Integration of microbiome and cognitive data. - Machine learning for predictive modeling. - Sample size: 115 mother–child pairs.
Harmancıoğlu & Kabaran (2023) [183]	- Review of epigenetic mechanisms in hypothalamic programming by maternal diet. - DNA methylation changes in appetite-regulating genes persist postnatally. - Histone modifications affect chromatin accessibility in metabolic genes. - MicroRNA alterations contribute to transgenerational effects.	- Comprehensive literature review. - Focus on epigenetic mechanisms in hypothalamic development. - Integration of animal model findings. - Discussion of reversibility and intervention potential.
Hasegawa et al. (2022) [184]	- Maternal obesity alters gestational metabolome with effects on infant brain and behavior. - Altered metabolites include amino acids, lipids, and neurotransmitter precursors. - Metabolomic signatures predict infant neurodevelopmental outcomes. - Rhesus macaque model shows translational relevance.	- Rhesus macaque model of maternal obesity. - Comprehensive metabolomics of maternal and fetal samples. - Infant neurobehavioral assessment. - Brain MRI for structural analysis. - Integration of metabolomic and neurodevelopmental data. - Sample size: 35 mother-infant pairs.
Hinkle et al. (2012) [185]	- J-shaped association between maternal BMI and child neurodevelopment. - Both underweight and obesity associated with developmental delays. - Stronger effects for severe obesity (BMI >35). - Associations vary by developmental domain assessed.	- Analysis of Early Childhood Longitudinal Study-Birth Cohort. - Bayley Scales administered at 2 years. - Maternal BMI from self-report. - Complex survey analysis methods. - Adjustment for sociodemographic factors. - Sample size: 6850 mother–child pairs.
Huang et al. (2014) [186]	- Maternal obesity associated with lower offspring IQ throughout childhood.- Effects emerge early and persist through age 7. - Dose–response relationship across maternal BMI categories. - Mediation by pregnancy complications and socioeconomic factors.	- Collaborative Perinatal Project data analysis. - Serial cognitive assessments from 8 months to 7 years. - Maternal pre-pregnancy BMI from measured data. - Mixed effects models for longitudinal analysis. - Mediation analysis for pathways. - Sample size: 34,240 mother–child pairs.
Keimpema et al. (2013) [187]	- Endocannabinoid system disruption links maternal obesity to offspring neurodevelopment. - Altered CB1 receptor signaling affects neuronal migration and synaptogenesis. - Changes in endocannabinoid metabolism in developing brain. - Potential target for therapeutic intervention.	- Review of endocannabinoid system in developmental programming. - Integration of molecular and cellular mechanisms. - Analysis of human and animal model data. - Discussion of therapeutic implications.
Kim & Park (2018) [188]	- Physical exercise improves cognitive deficits in offspring of obese mothers. - Exercise enhances hippocampal neurogenesis and reduces apoptosis. - Restoration of BDNF signaling and synaptic plasticity. - Suggests postnatal intervention can mitigate prenatal programming.	- Rat model of maternal obesity. - Offspring exercise intervention (treadmill running). - Cognitive testing (Morris water maze, novel object recognition). - Hippocampal histology and molecular analysis. - Assessment of neurogenesis and apoptosis markers.
Krakowiak et al. (2012) [189]	- Maternal metabolic conditions associated with increased autism and developmental delay risk. - Maternal obesity: ASD OR = 1.67, DD OR = 2.07. - Combined obesity and diabetes showed highest risks. - Effects on expressive language particularly pronounced.	- CHARGE case–control study. - Comprehensive autism diagnostic assessment. - Maternal metabolic conditions from medical records and interview. - Multivariable logistic regression. - Sample size: 1004 children (517 ASD, 172 DD, 315 typical).
Krzeczkowski et al. (2018) [190]	- Maternal adiposity associated with child neurodevelopmental problems at 3–4 years. - Hyperglycemia showed independent effects on behavioral outcomes. - Sex-specific effects observed for some associations. - Non-linear relationships for behavioral outcomes.	- MIREC cohort study analysis. - Multiple neurodevelopmental assessments at 3–4 years. - Maternal metabolic markers from pregnancy. - Structural equation modeling. - Sex-stratified analyses. - Sample size: 1868 mother–child pairs.
Lee et al. (2023) [191]	- Maternal obesity affects uncinate fasciculus white matter in preterm infants. - Reduced fractional anisotropy (0.42 vs. 0.46, *p* < 0.001). - White matter changes predict later neurodevelopmental outcomes. - Effects most pronounced in very preterm infants.	- Prospective study of preterm infants. - DTI at term-equivalent age. - Tract-based spatial statistics analysis. - Neurodevelopmental follow-up at 18–24 months. - Sample size: 92 preterm infants.
Levin (2010) [192]	- Interaction of genetic predisposition and perinatal environment in obesity programming. - Critical role of leptin and insulin signaling in hypothalamic development. - Identification of sensitive periods for metabolic programming. - Emphasis on gene-environment interactions.	- Review of neural pathways in energy homeostasis. - Integration of genetic and environmental factors. - Analysis of critical developmental periods. - Discussion of intervention strategies.
Li et al. (2016) [193]	- Maternal obesity associated with altered neonatal brain functional connectivity. - Reduced connectivity in default mode network regions. - Changes in thalamo-cortical connectivity patterns. - Functional alterations present at 2 weeks of age.	- Resting-state fMRI in sleeping neonates. - Seed-based connectivity analysis. - Maternal BMI from medical records. - Adjustment for confounding variables. - Sample size: 28 neonates (14 from obese, 14 from normal-weight mothers).
Lippert & Brüning (2021) [194]	- Comprehensive review linking maternal metabolism to offspring psychiatric disorders. - Integration of metabolic and neurodevelopmental pathways. - Emphasis on hypothalamic-pituitary axis programming. - Discussion of unified mechanisms across disorders.	- Systematic review of literature. - Focus on mechanistic pathways. - Integration of preclinical and clinical evidence. - Theoretical framework development.
Liu et al. (2021) [195]	- High-fiber diet mitigates maternal obesity effects on offspring cognition and behavior. - Restoration of gut microbiota diversity and composition. - Improved synaptic plasticity and reduced neuroinflammation. - Gut–brain axis modulation as therapeutic target.	- Mouse model of maternal obesity with dietary intervention. - Offspring behavioral testing battery. - Gut microbiome analysis (16 S sequencing). - Brain histology and molecular analysis. - Metabolomics of serum and brain tissue.
Luo et al. (2021) [196]	- Maternal BMI associated with offspring brain food cue reactivity. - Increased activation in reward regions to high-calorie food images. - Altered connectivity between prefrontal and subcortical regions. - Neural changes predict eating behaviors.	- fMRI study of children viewing food images. - Maternal pre-pregnancy BMI from medical records. - Brain activation and connectivity analyses. - Eating behavior questionnaires. - Sample size: 52 children aged 7–11 years.
Menting et al. (2018) [197]	- Maternal overweight/obesity associated with child behavioral problems and executive dysfunction. - Stronger associations for externalizing than internalizing behaviors. - Effects partially mediated by pregnancy complications. - Dose–response relationships observed.	- ABCD cohort study analysis. - Child behavior assessed with SDQ and CBCL. - Executive function measured with validated tasks. - Maternal BMI from early pregnancy. - Structural equation modeling. - Sample size: 3233 mother–child pairs.
Mina et al. (2017) [198]	- Very severe maternal obesity (BMI ≥ 40) associated with impaired neurodevelopment. - 3.3-fold increased odds of developmental delay. - 2.6-fold increased risk of executive function problems. - Effects independent of socioeconomic factors.	- Prospective cohort study. - Comprehensive neurodevelopmental assessment at 2–5 years. - Maternal BMI categories from antenatal records. - Multiple domains assessed (cognitive, motor, behavioral). - Sample size: 272 children.
Mina et al. (2016) [199]	- Severe maternal obesity associated with adverse neuropsychiatric outcomes. - Increased risk of ADHD symptoms (OR = 2.4). - Higher rates of emotional difficulties and peer problems. - Associations stronger for severe versus moderate obesity.	- Edinburgh cohort longitudinal study. - Strengths and Difficulties Questionnaire. - Clinical assessments for ADHD. - Maternal BMI from booking visit. - Adjustment for multiple confounders. - Sample size: 378 children at 5-year follow-up.
Monthé-Drèze et al. (2018) [200]	- Maternal obesity effects on cognition partially mediated by inflammation. - IL-6 and CRP levels explain 20% of association. - After adjusting for maternal CRP, offspring showed 1.8 points lower cognitive scores. - Suggests anti-inflammatory interventions may help.	- Project Viva cohort mechanistic analysis. - Maternal inflammatory markers in pregnancy. - Child IQ assessment at school age. - Formal mediation analysis. - Adjustment for socioeconomic factors. - Sample size: 872 mother–child pairs.
Morgan et al. (2020) [201]	- Prenatal maternal CRP predicts child executive function at 4–6 years. - Higher CRP associated with poorer working memory and inhibitory control. - Effects independent of maternal BMI and other factors. - Inflammation as targetable mechanism.	- Community Child Health Network study. - Maternal CRP in third trimester. - Executive function battery at follow-up. - Path analysis for direct and indirect effects. - Multi-site diverse sample. - Sample size: 418 mother–child pairs.
Na et al. (2021) [202]	- Maternal obesity associated with lower cortical thickness in neonate brain. - Regional differences most pronounced in frontal and temporal areas. - Cortical thickness correlated with maternal inflammatory markers. - Changes visible within first month of life.	- High-resolution structural MRI in neonates. - Cortical thickness analysis using FreeSurfer. - Maternal BMI and metabolic markers. - Correlation with inflammatory biomarkers. - Sample size: 44 healthy neonates.
Ou et al. (2015) [203]	- Maternal adiposity negatively affects infant white matter development. - Lower fractional anisotropy in multiple brain regions. - Changes present at 2 weeks of age. - Correlation with maternal metabolic markers.	- DTI of healthy neonates. - Voxel-wise analysis of white matter integrity. - Maternal body composition by air displacement plethysmography. - Correlation with metabolic and inflammatory markers. - Sample size: 32 mother-infant pairs.
Page et al. (2019) [204]	- Children exposed to maternal obesity show hypothalamic alterations. - 4% volume reduction in exposed versus unexposed. - Functional connectivity changes in appetite networks. - Alterations predict future weight gain.	- Brain MRI in children aged 7–11 years.- Hypothalamic segmentation and volumetry.- Resting-state connectivity analysis.- Longitudinal weight trajectory modeling.- Sample size: 165 children from BrainChild study.
Panagos et al. (2016) [205]	- Breast milk from obese mothers shows pro-inflammatory profile. - Reduced neuroprotective factors (lower DHA, choline). - Higher inflammatory cytokines (IL-6, TNF-α). - Milk composition correlates with infant neurodevelopment.	- Breast milk collection and analysis. - Comprehensive fatty acid profiling. - Cytokine and growth factor measurement. - Dietary inflammatory index calculation. - Correlation with infant development. - Sample size: 45 exclusively breastfeeding mothers.
Park et al. (2019) [206]	- Maternal obesity causes ER stress in developing hypothalamus. - Disrupted neuronal projections from ARH to PVH. - Altered leptin signaling and STAT3 phosphorylation. - ER stress inhibition partially rescues phenotype.	- Mouse model of maternal high-fat diet. - Analysis of hypothalamic ER stress markers. - Neuroanatomical tracing of projections. - Chemical chaperone intervention studies. - Metabolic phenotyping of offspring.
Parsaei et al. (2024) [207]	- Systematic review of MRI studies on maternal obesity effects. - Consistent findings of reduced gray matter volumes. - White matter integrity alterations in multiple tracts. - Functional connectivity disruptions in cognitive networks.	- Systematic review following PRISMA guidelines. - Focus on neuroimaging studies only. - Quality assessment of included studies. - Synthesis of structural and functional findings. - Included 28 studies.
Plucińska & Barger (2018) [208]	- Commentary on sex-specific reprogramming of executive brain centers. - Males show greater prefrontal cortex disruption. - Females demonstrate more subcortical alterations. - Implications for sex-specific interventions.	- Expert commentary on recent findings. - Integration of molecular and behavioral data. - Discussion of mechanisms underlying sex differences. - Future research recommendations.
Rafiq et al. (2023) [209]	- Integrated multi-omics reveals biomarkers of childhood obesity. - Gut microbiome signatures distinguish obesity risk groups. - Serum metabolites correlate with neurodevelopmental outcomes. - Machine learning identifies predictive biomarker panels.	- Birth cohort with multi-omics profiling. - Gut microbiome 16 S sequencing. - Serum metabolomics by mass spectrometry. - Machine learning for biomarker discovery. - Clinical outcome validation. - Sample size: 236 infants followed to 5 years.
Ross & Desai (2014) [210]	- Review of appetite/satiety programming by maternal obesity. - Focus on hypothalamic neuropeptide systems. - Analysis of leptin resistance development. - Discussion of critical periods for intervention.	- Comprehensive literature review. - Integration of animal model data. - Mechanistic pathway analysis. - Clinical translation discussion.
Salzwedel et al. (2019) [211]	- Maternal adiposity influences neonatal brain functional connectivity. - Altered connectivity in sensorimotor and visual networks. - Changes correlate with maternal metabolic markers. - Early emergence of functional brain differences.	- Resting-state fMRI in 2-week-old infants. - Independent component analysis. - Network connectivity assessment. - Maternal body composition measures. - Sample size: 96 healthy neonates.
Samara et al. (2020) [212]	- Neuroinflammation and white matter changes in obesity. - Diffusion basis spectrum imaging reveals microstructural alterations. - Correlation between inflammation markers and brain changes. - Implications for understanding developmental effects.	- Advanced diffusion MRI techniques. - Inflammatory biomarker assessment. - White matter integrity analysis. - Correlation of imaging and blood markers. - Adult study with developmental implications.
Sanchez et al. (2018) [213]	- Meta-analysis confirms maternal obesity-child neurodevelopment link. - Overall effect size: Cohen’s d = 0.16 (95% CI: 0.11–0.21). - Stronger effects for severe obesity and male offspring. - Publication bias assessment suggests robust findings.	- Systematic review and meta-analysis. - Multiple database search through 2017. - Random effects models. - Moderator analyses for obesity severity and child sex. - Quality assessment using Newcastle–Ottawa Scale. - 32 studies included.
Sanders et al. (2014) [214]	- Maternal IL-6 leads to reduced NPY innervation in PVH. - Elevated IL-6 associated with reduced neurite growth. - Altered Netrin-1 and receptor expression. - Mechanism for obesity-induced neural connectivity disruption.	- Cell culture model of hypothalamic neurons. - IL-6 treatment experiments. - Immunocytochemistry for neural markers. - Gene expression analysis. - In vivo validation in mouse model.
Sanguinetti et al. (2019) [215]	- Maternal obesity alters offspring microbiota affecting behavior. - Reduced memory and exploratory behavior in exposed mice. - Specific bacterial taxa correlate with behavioral outcomes. - Microbiota transplantation partially transfers phenotype.	- Mouse model of maternal obesity. - Comprehensive behavioral testing battery. - Gut microbiome sequencing and analysis. - Microbiota transplantation experiments. - Correlation of microbiome-behavior data.
Sarker & Peleg-Raibstein (2018) [216]	- Maternal overnutrition induces cognitive deficits across generations. - F1 and F2 offspring show impaired learning and memory. - Epigenetic modifications in brain tissue persist. - Evidence for transgenerational inheritance.	- Multi-generational mouse study. - Cognitive testing across three generations. - Epigenetic analysis of brain tissue. - Gene expression profiling. - Sperm methylation analysis.
Saros et al. (2023) [217]	- Maternal obesity and GDM show additive effects on neurodevelopment. - Obesity alone: 0.3 SD reduction in language. - Obesity + GDM: 0.6 SD reduction. - Diet quality modifies associations.	- Finnish birth cohort study. - Neurodevelopmental assessment at 2 years. - Maternal diet quality evaluation. - Statistical interaction testing. - Sample size: 439 mother–child pairs.
Schmidt et al. (2021) [218]	- Maternal metabolic profile predicts neuroinflammation in offspring. - Specific metabolites associated with atypical neurodevelopment. - Machine learning identifies predictive metabolic signatures. - Links metabolism to brain immune activation.	- Case–control study design. - Maternal plasma metabolomics. - Child neurodevelopmental assessment. - Inflammatory marker measurement. - Pathway enrichment analysis. - Sample size: 450 mother–child pairs.
Shapiro et al. (2020) [219]	- In utero exposure to maternal obesity alters brain function in children. - Reduced prefrontal activation during cognitive tasks. - Altered default mode network connectivity. - Functional changes correlate with behavioral measures.	- fMRI during working memory task. - Resting-state connectivity analysis. - Maternal pre-pregnancy BMI documentation. - Cognitive and behavioral assessments. - Sample size: 88 children aged 7–9 years.
Skowronski et al. (2023) [220]	- Review of neurodevelopmental programming of adiposity. - Integration of central and peripheral mechanisms. - Analysis of critical periods and intervention windows. - Emphasis on translational implications.	- Narrative review of recent literature. - Focus on bidirectional brain-adipose communication. - Discussion of therapeutic targets. - Clinical translation framework.
Stachowiak et al. (2013) [221]	- Maternal obesity affects fetal brain gene expression. - Altered expression of neurodevelopmental genes. - Changes in cellular development pathways. - Early molecular basis for later dysfunction.	- Analysis of fetal brain tissue. - Gene expression microarray. - Pathway enrichment analysis. - Validation by qRT-PCR. - Correlation with maternal metabolic status.
Sullivan et al. (2015) [222]	- Maternal high-fat diet programs neuroendocrine system. - Altered HPA axis responsivity in offspring. - Changes in stress-related behaviors. - Sex-specific programming effects.	- Non-human primate model. - Comprehensive behavioral assessment. - Neuroendocrine function testing. - Brain tissue molecular analysis. - Longitudinal follow-up design.
Tanda et al. (2013) [223]	- Pre-pregnancy obesity impacts children’s cognitive test scores. - Math scores: −2.8 points for maternal obesity. - Reading scores: −3.1 points for maternal obesity. - Effects persist after extensive confounder adjustment.	- ECLS-K dataset analysis. - Standardized achievement tests. - Maternal BMI from self-report. - Propensity score matching. - Sensitivity analyses. - Sample size: 6600 children.
Torres-Espínola et al. (2015) [224]	- Maternal obesity affects neurodevelopment at 6 and 18 months. - Greater effects at 18 months suggesting progressive impact. - GDM shows additional independent effects. - Dose–response relationship with maternal BMI.	- PREOBE cohort longitudinal study. - Bayley Scales at 6 and 18 months. - Maternal metabolic assessment in pregnancy. - Comprehensive confounder adjustment. - Sample size: 331 mother-infant pairs.
Urbonaite et al. (2022) [225]	- Maternal HFD causes inflammatory activation and gut dysbiosis. - Offspring show autism-like and ADHD-like behaviors. - Effects observed with both prenatal and postnatal exposure. - Microglial activation in key brain regions.	- Mouse model with cross-fostering design. - Comprehensive behavioral phenotyping. - Brain histology for microglial activation. - Gut microbiome analysis. - Cytokine profiling.
Walker et al. (2008) [226]	- Perinatal maternal fat intake affects offspring hippocampus. - Altered gene expression in metabolic pathways. - Reduced neurogenesis markers. - Impaired spatial memory performance.	- Rat model of maternal high-fat diet. - Hippocampal gene expression analysis. - Behavioral testing (Morris water maze). - Neurogenesis assessment (BrdU labeling). - Metabolic phenotyping.
Widen et al. (2019) [227]	- Pre-pregnancy obesity associated with lower cognitive scores in boys. - No significant association in girls. - Effects evident in low-income, multiethnic population. - Environmental factors may modify associations.	- Columbia Center birth cohort. - WISC-IV cognitive assessment at age 7. - Maternal pre-pregnancy BMI from self-report. - Analysis of effect modification by environmental factors. - Sample size: 368 mother–child pairs.
Wu et al. (2013) [228]	- Maternal obesity causes reversal learning deficits in offspring. - Striatal dopamine system disturbances identified. - Reduced D2 receptor expression. - Altered reward processing behaviors.	- Rat model of maternal cafeteria diet. - Reversal learning paradigm. - Striatal dopamine analysis (HPLC). - Receptor binding studies. - Gene expression analysis.
Yeung et al. (2017) [229]	- Both maternal and paternal obesity affect child development. - Maternal effects stronger than paternal. - Multiple developmental domains affected. - Suggests both intrauterine and genetic/environmental factors.	- UPSTATE cohort analysis. - Ages and Stages Questionnaire. - Both parents’ BMI collected. - Longitudinal assessments to 3 years. - Sample size: 5000 families.
Zhu et al. (2018) [230]	- Combined maternal obesity and offspring HFD worsen cognition. - Synergistic effects on hippocampal function. - Exacerbated neuroinflammation. - Male-specific vulnerability.	- Two-hit mouse model design. - Cognitive testing battery. - Hippocampal molecular analysis. - Inflammatory marker assessment. - Sex-stratified analyses.

**Table 2:** Maternal Obesity Effects Across Developmental Stages.

Developmental Stage	Primary Outcomes	Studies (*n*)	Effect Size Range	Consistency	Clinical Significance
**Prenatal**	Fetal brain structure/function	12	d = −0.3 to −0.7	High	Moderate to High
	Birth outcomes	18	OR = 1.4–2.3	Very High	Moderate
**Infancy (0–2 years)**	General development	18	d = −0.2 to −0.4	High	Moderate
	Language development	14	d = −0.3 to −0.5	Very High	Moderate to High
	Motor development	8	d = −0.1 to −0.4	Moderate	Low to Moderate
**Preschool (3–5 years)**	Cognitive abilities	19	d = −0.2 to −0.4	High	Moderate
	Executive function	15	d = −0.3 to −0.6	Very High	High
	Language/communication	12	d = −0.3 to −0.5	High	Moderate to High
	Behavioral regulation	11	d = −0.2 to −0.4	High	Moderate
**School-age (6–11 years)**	Academic achievement	16	d = −0.2 to −0.5	High	Moderate to High
	Executive function	12	d = −0.2 to −0.4	High	High
	ADHD symptoms	8	OR = 1.2–1.6	High	Moderate
	Social functioning	6	d = −0.2 to −0.3	Moderate	Moderate
**Adolescence (12+ years)**	Cognitive abilities	2	d = −0.3 to −0.4	Limited data	Moderate
	Mental health	2	OR = 1.3–1.5	Limited data	High

**Table 3:** Comparative Effect Sizes Across Neurodevelopmental Domains.

Functional Domain	Specific Outcome	Effect Size (95% CI)	Studies (*n*)	Clinical Significance	Population Impact
**Cognitive Abilities**					
General Intelligence	Full-Scale IQ	−0.18 (−0.28, −0.08)	23	Modest	Moderate
	Verbal IQ	−0.25 (−0.36, −0.14)	18	Moderate	High
	Performance IQ	−0.12 (−0.23, −0.01)	15	Small	Low
Language Development	Vocabulary (PPVT)	−0.28 (−0.41, −0.15)	18	Moderate	High
	Expressive Language	−0.32 (−0.48, −0.16)	14	Moderate	High
	Reading Comprehension	−0.33 (−0.52, −0.14)	12	Moderate	High
Memory & Learning	Working Memory	−0.31 (−0.48, −0.14)	13	Moderate	Moderate
	Long-term Memory	−0.26 (−0.42, −0.10)	11	Moderate	Moderate
**Executive Function**					
Attention	Sustained Attention	−0.44 (−0.62, −0.26)	15	Large	High
	Selective Attention	−0.38 (−0.57, −0.19)	13	Moderate	High
Inhibitory Control	Response Inhibition	−0.41 (−0.59, −0.23)	12	Moderate	High
	Interference Control	−0.39 (−0.56, −0.22)	10	Moderate	High
Working Memory	Verbal WM	−0.36 (−0.53, −0.19)	11	Moderate	High
	Spatial WM	−0.22 (−0.38, −0.06)	8	Small	Moderate
Cognitive Flexibility	Set-shifting	−0.29 (−0.46, −0.12)	9	Moderate	Moderate
**Behavioral Outcomes**					
ADHD Symptoms	Hyperactivity	OR: 1.62 (1.45, 1.81)	20	Large	Very High
	Inattention	OR: 1.47 (1.32, 1.64)	18	Large	High
	Combined Type	OR: 1.73 (1.52, 1.97)	14	Large	Very High
Internalizing	Anxiety	OR: 1.34 (1.18, 1.52)	16	Moderate	Moderate
	Depression	OR: 1.29 (1.11, 1.50)	12	Moderate	Moderate
Externalizing	Aggression	OR: 1.51 (1.33, 1.71)	17	Large	High
	Oppositional	OR: 1.43 (1.26, 1.62)	15	Large	High

**Table 4:** Developmental Timing and Domain-Specific Vulnerability Patterns.

Domain	Early Childhood (2–5 Years)	School Age (6–11 Years)	Adolescence (12–18 Years)	Persistence Pattern
**Cognitive**				
General IQ	−0.15 (−0.28, −0.02)	−0.21 (−0.35, −0.07)	−0.18 (−0.33, −0.03)	Stable
Language	−0.35 (−0.52, −0.18)	−0.28 (−0.44, −0.12)	−0.22 (−0.39, −0.05)	Decreasing
Working Memory	−0.25 (−0.41, −0.09)	−0.38 (−0.55, −0.21)	−0.31 (−0.49, −0.13)	Peak School Age
**Executive Function**				
Attention	−0.32 (−0.49, −0.15)	−0.48 (−0.66, −0.30)	−0.41 (−0.59, −0.23)	Peak School Age
Inhibition	−0.28 (−0.45, −0.11)	−0.44 (−0.62, −0.26)	−0.38 (−0.56, −0.20)	Peak School Age
Flexibility	−0.18 (−0.35, −0.01)	−0.34 (−0.51, −0.17)	−0.29 (−0.46, −0.12)	Peak School Age
**Behavioral**				
ADHD	OR: 1.45 (1.25, 1.68)	OR: 1.71 (1.52, 1.92)	OR: 1.58 (1.38, 1.81)	Peak School Age
Internalizing	OR: 1.22 (1.05, 1.42)	OR: 1.31 (1.14, 1.51)	OR: 1.44 (1.23, 1.68)	Increasing
Externalizing	OR: 1.48 (1.29, 1.70)	OR: 1.52 (1.33, 1.74)	OR: 1.41 (1.22, 1.63)	Stable-Decreasing

## Data Availability

No new data were created or analyzed in this study. Data sharing is not applicable to this article.

## Supplementary Materials

The following supporting information can be downloaded at: https://www.mdpi.com/article/10.3390/healthcare13202653/s1, Table S1. Full Table of the Studies (*n* = 78) included in the Umbrella Review, Table S2. Abbreviation list, Table S3. PRISMA 2020 Checklist. Table S4, Summary of Domain-Specific Neurodevelopmental Outcomes Associated with Maternal Obesity. Reference [242] has been cited in Supplementary Materials.

## References

[1] Grieger J.A., Hutchesson M.J., Cooray S.D., Khomami M.B., Zaman S., Segan L., Teede H., Moran L.J. (2021). A review of maternal overweight and obesity and its impact on cardiometabolic outcomes during pregnancy and postpartum. Ther. Adv. Reprod. Health.

[2] Lourenço J., Guedes-Martins L. (2025). Pathophysiology of maternal obesity and hypertension in pregnancy. J. Cardiovasc. Dev. Dis..

[3] Haque R., Keramat S.A., Rahman S.M., Mustafa M.U.R., Alam K. (2021). Association of maternal obesity with fetal and neonatal death: Evidence from South and South-East Asian countries. PLoS ONE.

[4] Reichetzeder C. (2021). Overweight and obesity in pregnancy: Their impact on epigenetics. Eur. J. Clin. Nutr..

[5] Reed J., Case S., Rijhsinghani A. (2023). Maternal obesity: Perinatal implications. SAGE Open Med..

[6] Daliry A., Pereira E.N.G.d.S. (2021). Role of Maternal Microbiota and Nutrition in Early-Life Neurodevelopmental Disorders. Nutrients.

[7] Gantenbein K.V., Kanaka-Gantenbein C. (2022). Highlighting the trajectory from intrauterine growth restriction to future obesity. Front. Endocrinol..

[8] Lubrano C., Parisi F., Cetin I. (2024). Impact of maternal environment and inflammation on fetal neurodevelopment. Antioxidants.

[9] Papachatzi E., Papadopoulos V., Dimitriou G., Paparrodopoulos S., Papadimitriou-Olivgeris M., Vantarakis A. (2014). Prepregnancy maternal obesity and fetal-perinatal death in a Mediterranean country. JPME.

[10] Denizli M., Capitano M.L., Kua K.L. (2022). Maternal obesity and the impact of associated early-life inflammation on long-term health of offspring. Front. Cell. Infect. Microbiol..

[11] Apostolopoulou A., Tranidou A., Tsakiridis I., Magriplis E., Dagklis T., Chourdakis M. (2024). Effects of Nutrition on Maternal Health, Fetal Development, and Perinatal Outcomes. Nutrients.

[12] Gkintoni E., Panagioti M., Vassilopoulos S.P., Nikolaou G., Boutsinas B., Vantarakis A. (2025). Leveraging AI-Driven Neuroimaging Biomarkers for Early Detection and Social Function Prediction in Autism Spectrum Disorders: A Systematic Review. Healthcare.

[13] Rosberg A., Merisaari H., Lewis J.D., Hashempour N., Lukkarinen M., Rasmussen J.M., Scheinin N.M., Karlsson L., Karlsson H., Tuulari J.J. (2024). Associations between maternal pre-pregnancy BMI and infant striatal mean diffusivity. BMC Med..

[14] Na X., Mackean P.P., Cape G.A., Johnson J.W., Ou X. (2024). Maternal nutrition during pregnancy and offspring brain development: Insights from neuroimaging. Nutrients.

[15] Hasebe K., Kendig M.D., Morris M.J. (2021). Mechanisms underlying the cognitive and behavioural effects of maternal obesity. Nutrients.

[16] Tong L., Kalish B.T. (2020). The impact of maternal obesity on childhood neurodevelopment. J. Perinatol..

[17] Saeed S., Bonnefond A., Froguel P. (2024). Obesity: Exploring its connection to brain function through genetic and genomic perspectives. Mol. Psychiatry.

[18] Norr M.E., Hect J.L., Lenniger C.J., Heuvel M.V.D., Thomason M.E. (2020). An examination of maternal prenatal BMI and human fetal brain development. J. Child Psychol. Psychiatry.

[19] Laughlin M., Cooke B., Boutelle K., Savage C.R., Kravitz A., Small D., Arvanitakis Z., Martin A., Stoeckel L.E. (2021). Neuroimaging and modulation in obesity and diabetes research: 10th anniversary meeting. Int. J. Obes..

[20] Kong L., Chen X., Gissler M., Lavebratt C. (2020). Relationship of prenatal maternal obesity and diabetes to offspring neurodevelopmental and psychiatric disorders: A narrative review. Int. J. Obes..

[21] Eleftheriades A., Koulouraki S., Belegrinos A., Eleftheriades M., Pervanidou P. (2025). Maternal obesity and neurodevelopment of the offspring. Nutrients.

[22] Chen S., Fan M., Lee B.K., Dalman C., Karlsson H., Gardner R.M. (2023). Rates of maternal weight gain over the course of pregnancy and offspring risk of neurodevelopmental disorders. BMC Med..

[23] Han V.X., Patel S., Jones H.F., Nielsen T.C., Mohammad S.S., Hofer M.J., Gold W., Brilot F., Lain S.J., Nassar N. (2021). Maternal acute and chronic inflammation in pregnancy is associated with common neurodevelopmental disorders: A systematic review. Transl. Psychiatry.

[24] Davis J., Mire E. (2021). Maternal obesity and developmental programming of neuropsychiatric disorders: An inflammatory hypothesis. Brain Neurosci. Adv..

[25] Jiménez-Osorio A.S., Carreón-Torres E., Correa-Solís E., Ángel-García J., Arias-Rico J., Jiménez-Garza O., Morales-Castillejos L., Díaz-Zuleta H.A., Baltazar-Tellez R.M., Sánchez-Padilla M.L. (2023). Inflammation and Oxidative Stress Induced by Obesity, Gestational Diabetes, and Preeclampsia in Pregnancy: Role of High-Density Lipoproteins as Vectors for Bioactive Compounds. Antioxidants.

[26] Cortés-Albornoz M.C., García-Guáqueta D.P., Velez-Van-Meerbeke A., Talero-Gutiérrez C. (2021). Maternal nutrition and neurodevelopment: A scoping review. Nutrients.

[27] Gkintoni E., Halkiopoulos C. (2025). Digital Twin Cognition: AI-Biomarker Integration in Biomimetic Neuropsychology. Biomimetics.

[28] Kankowski L., Ardissino M., McCracken C., Lewandowski A.J., Leeson P., Neubauer S., Harvey N.C., Petersen S.E., Raisi-Estabragh Z. (2022). The impact of maternal obesity on offspring cardiovascular health: A systematic literature review. Front. Endocrinol..

[29] Mannino A., Sarapis K., Moschonis G. (2022). The effect of maternal overweight and obesity pre-pregnancy and during childhood in the development of obesity in children and adolescents: A systematic literature review. Nutrients.

[30] Berrigan D., Arteaga S.S., Colón-Ramos U., Rosas L.G., Monge-Rojas R., O’Connor T.M., Pérez-Escamilla R., Roberts E.F.S., Sanchez B., Téllez-Rojo M.M. (2021). Measurement challenges for childhood obesity research within and between Latin America and the United States. Obes. Rev..

[31] Rodriguez A.C.I., Nagpal T.S. (2021). The WOMBS framework: A review and new theoretical model for investigating pregnancy-related weight stigma and its intergenerational implications. Obes. Rev..

[32] Dodd J.M., Louise J., Deussen A.R., Mitchell M., Poston L. (2024). Rethinking causal assumptions about maternal BMI, gestational weight gain, and adverse pregnancy outcomes. BMC Med..

[33] Yu H., Li M., Qian G., Yue S., Ossowski Z., Szumilewicz A. (2024). A systematic review and Bayesian network meta-analysis comparing in-person, remote, and blended interventions in physical activity, diet, education, and behavioral modification on gestational weight gain among overweight or obese pregnant individuals. Adv. Nutr. Int. Rev. J..

[34] Nagi M.A., Ahmed H., Rezq M.A.A., Sangroongruangsri S., Chaikledkaew U., Almalki Z., Thavorncharoensap M. (2023). Economic costs of obesity: A systematic review. Int. J. Obes..

[35] Indarti J., Susilo S.A., Hyawicaksono P., Berguna J.S.N., Tyagitha G.A., Ikhsan M. (2021). Maternal and perinatal outcome of maternal obesity at RSCM in 2014–2019. Obstet. Gynecol. Int..

[36] Escobar M.B.C., Contreras J.O., Bertoglia M.P., Bannout M.A. (2021). Pregestational obesity, maternal morbidity and risk of caesarean delivery in a country in an advanced stage of obstetric transition. Obes. Res. Clin. Pract..

[37] Fakhraei R., Denize K., Simon A., Sharif A., Zhu-Pawlowsky J., Dingwall-Harvey A.L.J., Hutton B., Pratt M., Skidmore B., Ahmadzai N. (2022). Predictors of adverse pregnancy outcomes in pregnant women living with obesity: A systematic review. Int. J. Environ. Res. Public Health.

[38] Khalifa E., El-Sateh A., Zeeneldin M., Abdelghany A.M., Hosni M., Abdallah A., Salama S., Abdel-Rasheed M., Mohammad H. (2021). Effect of maternal BMI on labor outcomes in primigravida pregnant women. BMC Pregnancy Childbirth.

[39] Strauss A., Rochow N., Kunze M., Hesse V., Dudenhausen J.W., Voigt M. (2021). Obesity in pregnant women: A 20-year analysis of the German experience. Eur. J. Clin. Nutr..

[40] Van Lieshout R.J., Taylor V.H., Boyle M.H. (2011). Pre-pregnancy and pregnancy obesity and neurodevelopmental outcomes in offspring: A systematic review. Obes Rev..

[41] Bordeleau M., de Cossío L.F., Chakravarty M.M., Tremblay M. (2021). From maternal diet to neurodevelopmental disorders: A story of neuroinflammation. Front. Cell. Neurosci..

[42] Rubini E., Schenkelaars N., Rousian M., Sinclair K.D., Wekema L., Faas M.M., Steegers-Theunissen R.P., Schoenmakers S. (2022). Maternal obesity during pregnancy leads to derangements in one-carbon metabolism and the gut microbiota: Implications for fetal development and offspring wellbeing. Am. J. Obstet. Gynecol..

[43] Basak S., Das R.K., Banerjee A., Paul S., Pathak S., Duttaroy A.K. (2022). Maternal obesity and gut microbiota are associated with fetal brain development. Nutrients.

[44] Hufnagel A., Dearden L., Fernandez-Twinn D.S., Ozanne S.E. (2022). Programming of cardiometabolic health: The role of maternal and fetal hyperinsulinaemia. J. Endocrinol..

[45] Strain J., Spaans F., Serhan M., Davidge S.T., Connor K.L. (2022). Programming of weight and obesity across the lifecourse by the maternal metabolic exposome: A systematic review. Mol. Asp. Med..

[46] Schoonejans J.M., Ozanne S.E. (2021). Developmental programming by maternal obesity: Lessons from animal models. Diabet. Med..

[47] Rivera H.M., Christiansen K.J., Sullivan E.L. (2015). The role of maternal obesity in the risk of neuropsychiatric disorders. Front. Neurosci..

[48] Alum E.U. (2025). Metabolic Memory in Obesity: Can Early-Life Interventions Reverse Lifelong Risks?. Obes. Med..

[49] Rodrigo N., Saad S., Pollock C., Glastras S.J. (2022). Diet Modification before or during Pregnancy on Maternal and Foetal Outcomes in Rodent Models of Maternal Obesity. Nutrients.

[50] Brunner K., Linder T., Klaritsch P., Tura A., Windsperger K., Göbl C. (2025). The impact of overweight and obesity on pregnancy: A narrative review of physiological consequences, risks and challenges in prenatal care, and early intervention strategies. Curr. Diabetes Rep..

[51] Picó C., Reis F., Egas C., Mathias P., Matafome P. (2020). Lactation as a programming window for metabolic syndrome. Eur. J. Clin. Investig..

[52] Rousseau-Ralliard D., Chavatte-Palmer P., Couturier-Tarrade A. (2023). The effect of maternal exposure to a diet high in fats and cholesterol on the placental function and phenotype of the offspring in a rabbit model: A summary review of about 15 years of research. Int. J. Mol. Sci..

[53] Radford-Smith D.E., Anthony D.C. (2023). Mechanisms of maternal diet-induced obesity affecting the offspring brain and development of affective disorders. Metabolites.

[54] Samà M., Musillo C., Cirulli F. (2024). Counteracting the effects of maternal obesity on offspring neurodevelopment through Omega-3-based nutritional strategies. Neuroscience.

[55] Contu L., Hawkes C. (2017). A Review of the Impact of Maternal Obesity on the Cognitive Function and Mental Health of the Offspring. Int. J. Mol. Sci..

[56] Mortaji N., Krzeczkowski J.E., Boylan K., Booij L., Perreault M., Van Lieshout R.J. (2021). Maternal pregnancy diet, postnatal home environment and executive function and behavior in 3-to 4-y-olds. Am. J. Clin. Nutr..

[57] Ahmed S., Cano M.Á., Sánchez M., Hu N., Ibañez G. (2022). Effect of exposure to maternal diabetes during pregnancy on offspring’s brain cortical thickness and neurocognitive functioning. Child Neuropsychol..

[58] Ramírez V., González-Palacios P., Baca M.A., González-Domenech P.J., Fernández-Cabezas M., Álvarez-Cubero M.J., Rodrigo L., Rivas A. (2022). Effect of exposure to endocrine disrupting chemicals in obesity and neurodevelopment: The genetic and microbiota link. Sci. Total Environ..

[59] Yonatan E., Shukha O.N., Golani I., Abu-Ata S., Awad-Igbaria Y., Khatib N., Ginsberg Y., Palzur E., Beloosesky R., Shamir A. (2025). Maternal N-acetylcysteine supplementation in lactation ameliorates metabolic and cognitive deficits in adult offspring exposed to maternal obesity. Neuropharmacology.

[60] Dawson S.L., O’Hely M., Jacka F.N., Ponsonby A.L., Symeonides C., Loughman A., Collier F., Moreno-Betancur M., Sly P., Burgner D. (2021). Maternal prenatal gut microbiota composition predicts child behaviour. EBioMedicine.

[61] Buffington S.A., Di Prisco G.V., Auchtung T.A., Ajami N.J., Petrosino J.F., Costa-Mattioli M. (2016). Microbial Reconstitution Reverses Maternal Diet-Induced Social and Synaptic Deficits in Offspring. Cell.

[62] Volqvartz T., Andersen H.H.B., Pedersen L.H., Larsen A. (2023). Obesity in pregnancy—Long-term effects on offspring hypothalamic-pituitary-adrenal axis and associations with placental cortisol metabolism: A systematic review. Eur. J. Neurosci..

[63] Dow C., Lorthe E., Bernard J.Y., Galera C., Marchand-Martin L., Tafflet M., Ancel P., Charles M., Heude B. (2025). Maternal prepregnancy obesity and offspring intelligence quotient at 5 years: A multicohort analysis. Paediatr. Perinat. Epidemiol..

[64] Calcaterra V., Schneider L., Baresi S., Bodini F., Bona F., Chillemi C., De Silvestri A., Zanelli S., Zuccotti G. (2023). Specific learning disorders in children and adolescents with obesity. Children.

[65] Babaei M., Machle C.J., Mokhtari P., González J.O., Schmidt K.A., Alderete T.L., Adise S., Peterson B.S., Goran M.I. (2024). Pre-pregnancy maternal obesity and infant neurodevelopmental outcomes in Latino infants. Obesity.

[66] Hung L.Y., Margolis K.G. (2023). Autism spectrum disorders and the gastrointestinal tract: Insights into mechanisms and clinical relevance. Nat. Rev. Gastroenterol. Hepatol..

[67] Rodolaki K., Pergialiotis V., Iakovidou N., Boutsikou T., Iliodromiti Z., Kanaka-Gantenbein C. (2023). The impact of maternal diabetes on the future health and neurodevelopment of the offspring: A review of the evidence. Front. Endocrinol..

[68] Likhitweerawong N., Khorana J., Boonchooduang N., Phinyo P., Patumanond J., Louthrenoo O. (2022). Association between executive function and excess weight in pre-school children. PLoS ONE.

[69] Creese H., Hope S., Christie D., Goddings A., Viner R. (2021). Is earlier obesity associated with poorer executive functioning later in childhood? Findings from the Millennium Cohort Study. Pediatr. Obes..

[70] Smith B.L. (2021). Improving translational relevance: The need for combined exposure models for studying prenatal adversity. Brain Behav. Immun. Health.

[71] Musillo C., Creutzberg K.C., Collacchi B., Ajmone-Cat M.A., De Simone R., Lepre M., Amrein I., Riva M.A., Berry A., Cirulli F. (2023). Bdnf-Nrf-2 crosstalk and emotional behavior are disrupted in a sex-dependent fashion in adolescent mice exposed to maternal stress or maternal obesity. Transl. Psychiatry.

[72] Cernigliaro F., Santangelo A., Nardello R., Cascio S.L., D’agostino S., Correnti E., Marchese F., Pitino R., Valdese S., Rizzo C. (2024). Prenatal nutritional factors and neurodevelopmental disorders: A narrative review. Life.

[73] Shen F., Zhou H. (2024). Advances in the etiology and neuroimaging of children with attention deficit hyperactivity disorder. Front. Pediatr..

[74] Gkintoni E., Vantarakis A., Gourzis P. (2025). Neuroimaging Insights into the Public Health Burden of Neuropsychiatric Disorders: A Systematic Review of Electroencephalography-Based Cognitive Biomarkers. Medicina.

[75] Hao X., Lu J., Yan S., Tao F., Huang K. (2022). Maternal pre-pregnancy body mass index, gestational weight gain and children’s cognitive development: A birth cohort study. Nutrients.

[76] Kim C., Schilder N., Adolphus K., Berry A., Musillo C., Dye L., Cirulli F., Korosi A., Thuret S. (2024). The dynamic influence of nutrition on prolonged cognitive healthspan across the life course: A perspective review. Neurosci. Appl..

[77] Girchenko P., Lahti-Pulkkinen M., Lipsanen J., Heinonen K., Lahti J., Rantalainen V., Hämäläinen E., Laivuori H., Villa P.M., Kajantie E. (2022). Maternal early-pregnancy body mass index-associated metabolomic component and mental and behavioral disorders in children. Mol. Psychiatry.

[78] Zhang S., Lin T., Zhang Y., Liu X., Huang H. (2022). Effects of parental overweight and obesity on offspring’s mental health: A meta-analysis of observational studies. PLoS ONE.

[79] Karhunen V., Bond T.A., Zuber V., Hurtig T., Moilanen I., Järvelin M.-R., Evangelou M., Rodriguez A. (2021). The link between attention deficit hyperactivity disorder (ADHD) symptoms and obesity-related traits: Genetic and prenatal explanations. Transl. Psychiatry.

[80] Thorsheim C., Khan S., Lu Y., Kauffman R.P. (2025). Maternal exacerbating and protective factors that shape the prevalence and severity of child attention-deficit hyperactivity disorder: A narrative review. Front. Psychiatry.

[81] Havdahl A., Wootton R.E., Leppert B., Riglin L., Ask H., Tesli M., Askeland R.B., Hannigan L.J., Corfield E., Øyen A.-S. (2022). Associations between pregnancy-related predisposing factors for offspring neurodevelopmental conditions and Parental genetic liability to attention-deficit/hyperactivity disorder, autism, and schizophrenia. JAMA Psychiatry.

[82] Li L., Lagerberg T., Chang Z., Cortese S., Rosenqvist M.A., Almqvist C., D’Onofrio B.M., Hegvik T.A., Hartman C., Chen Q. (2020). Maternal pre-pregnancy overweight/obesity and the risk of attention-deficit/hyperactivity disorder in offspring: A systematic review, meta-analysis and quasi-experimental family-based study. Int. J. Epidemiol..

[83] Nieto-Ruiz A., Cerdó T., Jordano B., Torres-Espínola F.J., Escudero-Marín M., García-Ricobaraza M., Bermúdez M.G., García-Santos J.A., Suárez A., Campoy C. (2023). Maternal weight, gut microbiota, and the association with early childhood behavior: The PREOBE follow-up study. Child Adolesc. Psychiatry Ment. Health.

[84] Shahin S., Medley E.A., Naidu M., Trasande L., Ghassabian A. (2024). Exposure to organophosphate esters and maternal-child health. Environ. Res..

[85] Kacperska M., Mizera J., Pilecki M., Pomierny-Chamioło L. (2024). The impact of excessive maternal weight on the risk of neuropsychiatric disorders in offspring—A narrative review of clinical studies. Pharmacol. Rep..

[86] Shuffrey L.C., Morales S., Jacobson M.H., Enlow M.B., Ghassabian A., Margolis A.E., Lucchini M., Carroll K.N., Crum R.M., Dabelea D. (2023). Association of Gestational Diabetes Mellitus and perinatal maternal depression with early childhood behavioral problems: An Environmental Influences on Child Health Outcomes (ECHO) study. Child Dev..

[87] Gunter-Rahman F., Mallett S., White F., Jacques P., Raju R.M., Hivert M.-F., Lee E.A. (2025). Hypoxia in extravillous trophoblasts links maternal obesity and offspring neurobehavior. iScience.

[88] Wallander J.L., Berry S., Carr P.A., Peterson E.R., Waldie K.E., Marks E., D’Souza S., Morton S.M.B. (2021). Patterns of risk exposure in first 1000 days of life and health, behavior, and education-related problems at age 4.5: Evidence from Growing Up in New Zealand, a longitudinal cohort study. BMC Pediatr..

[89] Maitre L., Julvez J., López-Vicente M., Warembourg C., Tamayo-Uria I., Philippat C., Gützkow K.B., Guxens M., Andrusaityte S., Basagaña X. (2021). Early-life environmental exposure determinants of child behavior in Europe: A longitudinal, population-based study. Environ. Int..

[90] Niu L., Hanson S., Preciado-Becerra J., Eskandarani A., Lei X., Le M., Niu Z., Xie B. (2024). Psychosocial adjustment as a mediator in the relationship between childhood exposure to maternal depression and subsequent BMI and overweight risk. Children.

[91] Moog N.K., Cummings P.D., Jackson K.L., Aschner J.L., Barrett E.S., Bastain T.M., Blackwell C.K., Enlow M.B., Breton C.V., Bush N.R. (2023). Intergenerational transmission of the effects of maternal exposure to childhood maltreatment in the USA: A retrospective cohort study. Lancet Public Health.

[92] Mattila I., Nolvi S., Kataja E.-L., Tuulari J.J., Korja R., Scheinin N.M., Kaaja R., Karlsson H., Ekholm E., Karlsson L. (2025). Gestational diabetes mellitus and children’s social-emotional development, behavioral problems, and psychological adjustment. Pediatr. Res..

[93] Karakitsiou G., Plakias S., Christidi F., Tsiakiri A. (2024). Unraveling childhood obesity: A grounded theory approach to psychological, social, parental, and biological factors. Children.

[94] Sun Y., Luo D., Guan K., Luo X. (2024). Meeting 24-h movement behavior guidelines is associated with academic engagement, social-emotional functioning in obese/overweight youth. Complement. Ther. Clin. Pract..

[95] Lu J., Hao X., Zhu L., Guo Y., Wu X., Hao J., Tao F., Huang K. (2022). Non-linear and sex-specific effect of maternal pre-pregnancy BMI on emotional and behavioral development of preschool children: A population-based cohort study. Int. J. Environ. Res. Public Health.

[96] Shao S., Zhang Y., Liu J., Zeng C., Qin J., Liu Z., Zhang X. (2025). The long-term development outcomes of the offspring born to patients with systemic lupus erythematosus: A cross-sectional study. Eur. Child Adolesc. Psychiatry.

[97] Gao R., Liu X., Li X., Zhang Y., Wei M., Sun P., Zhang J., Cai L. (2022). Association between maternal sugar-sweetened beverage consumption and the social-emotional development of child before 1 year old: A prospective cohort study. Front. Nutr..

[98] Wu Y., De Asis-Cruz J., Limperopoulos C. (2024). Brain structural and functional outcomes in the offspring of women experiencing psychological distress during pregnancy. Mol. Psychiatry.

[99] Mentzelou M., Papadopoulou S.K., Psara E., Alexatou O., Koimtsidis T., Giaginis C. (2025). Exploring the impact of emotional eating in children: A narrative review. Pediatr. Rep..

[100] Kumar M., Jha A.K. (2024). Adolescent brain development: Limbic system and emotions. Encyclopedia of Religious Psychology and Behavior.

[101] Stephens K., Silk T.J., Anderson V., Hazell P., Enticott P.G., Sciberras E. (2020). Associations between limbic system white matter structure and socio-emotional functioning in children with ADHD + ASD. J. Autism Dev. Disord..

[102] Lei D., Li W., Qin K., Ai Y., Tallman M.J., Patino L.R., Welge J.A., Blom T.J., Klein C.C., Fleck D.E. (2022). Effects of short-term quetiapine and lithium therapy for acute manic or mixed episodes on the limbic system and emotion regulation circuitry in youth with bipolar disorder. Neuropsychopharmacology.

[103] Halkiopoulos C., Gkintoni E., Aroutzidis A., Antonopoulou H. (2025). Advances in Neuroimaging and Deep Learning for Emotion Detection: A Systematic Review of Cognitive Neuroscience and Algorithmic Innovations. Diagnostics.

[104] Sanaeifar F., Pourranjbar S., Pourranjbar M., Ramezani S., Mehr S.R., Wadan A.-H.S., Khazeifard F. (2024). Beneficial effects of physical exercise on cognitive-behavioral impairments and brain-derived neurotrophic factor alteration in the limbic system induced by neurodegeneration. Exp. Gerontol..

[105] Gkintoni E., Aroutzidis A., Antonopoulou H., Halkiopoulos C. (2025). From Neural Networks to Emotional Networks: A Systematic Review of EEG-Based Emotion Recognition in Cognitive Neuroscience and Real-World Applications. Brain Sci..

[106] Samson J.A., Newkirk T.R., Teicher M.H. (2023). Practitioner Review: Neurobiological consequences of childhood maltreatment—Clinical and therapeutic implications for practitioners. J. Child Psychol. Psychiatry.

[107] Xiao Q., Shen L., He H., Wang X., Fu Y., Ding J., Jiang F., Zhang J., Zhang Z., Grecucci A. (2024). Alteration of prefrontal cortex and its associations with emotional and cognitive dysfunctions in adolescent borderline personality disorder. Eur. Child Adolesc. Psychiatry.

[108] Fujihara H., Matsunaga M., Ueda E., Kajiwara T., Takeda A.K., Watanabe S., Baba K., Hagihara K., Myowa M. (2023). Altered gut microbiota composition is associated with difficulty in explicit emotion regulation in young children. Microorganisms.

[109] Bangma J.T., Hartwell H., Santos H.P., O’sHea T.M., Fry R.C. (2020). Placental programming, perinatal inflammation, and neurodevelopment impairment among those born extremely preterm. Pediatr. Res..

[110] Han V.X., Jones H.F., Patel S., Mohammad S.S., Hofer M.J., Alshammery S., Maple-Brown E., Gold W., Brilot F., Dale R.C. (2022). Emerging evidence of Toll-like receptors as a putative pathway linking maternal inflammation and neurodevelopmental disorders in human offspring: A systematic review. Brain Behav. Immun..

[111] Louwen F., Kreis N.-N., Ritter A., Yuan J. (2024). Maternal obesity and placental function: Impaired maternal–fetal axis. Arch. Gynecol. Obstet..

[112] Monaco-Brown M., Lawrence D.A. (2022). Obesity and maternal-placental-fetal immunology and health. Front. Pediatr..

[113] Gagnidze K., Pfaff D.W. (2022). Epigenetic mechanisms: DNA methylation and histone protein modification. Neuroscience in the 21st Century: From Basic to Clinical.

[114] Kakoulidou I., Avramidou E.V., Baránek M., Brunel-Muguet S., Farrona S., Johannes F., Kaiserli E., Lieberman-Lazarovich M., Martinelli F., Mladenov V. (2021). Epigenetics for crop improvement in times of global change. Biology.

[115] Cortés-Mancera F.M., Sarno F., Goubert D., Rots M.G. (2022). Gene-targeted DNA methylation: Towards long-lasting reprogramming of gene expression?. DNA Methyltransferases—Role and Function.

[116] Mauceri D. (2022). Role of Epigenetic Mechanisms in Chronic Pain. Cells.

[117] Akhter Z., Bi Z., Ali K., Sun C., Fiaz S., Haider F.U., Bai J. (2021). In response to abiotic stress, DNA methylation confers epigenetic changes in plants. Plants.

[118] Klibaner-Schiff E., Simonin E.M., Akdis C.A., Cheong A., Johnson M.M., Karagas M.R., Kirsh S., Kline O., Mazumdar M., Oken E. (2024). Environmental exposures influence multigenerational epigenetic transmission. Clin. Epigenet..

[119] Yuan M., Yang B., Rothschild G., Mann J.J., Sanford L.D., Tang X., Huang C., Wang C., Zhang W. (2023). Epigenetic regulation in major depression and other stress-related disorders: Molecular mechanisms, clinical relevance and therapeutic potential. Signal Transduct. Target. Ther..

[120] Aljabali A.A.A., Alkaraki A.K., Gammoh O., Tambuwala M.M., Mishra V., Mishra Y., Hassan S.S., El-Tanani M. (2024). Deciphering depression: Epigenetic mechanisms and treatment strategies. Biology.

[121] Kunysz M., Mora-Janiszewska O., Darmochwał-Kolarz D. (2021). Epigenetic modifications associated with exposure to endocrine disrupting chemicals in patients with gestational diabetes mellitus. Int. J. Mol. Sci..

[122] Blanco A.L.-Y., Díaz-López K.M., Vilchis-Gil J., Diaz-Garcia H., Gomez-Lopez J., Medina-Bravo P., Granados-Riveron J.T., Gallardo J.M., Klünder-Klünder M., Sánchez-Urbina R. (2022). Diet and maternal obesity are associated with increased oxidative stress in newborns: A cross-sectional study. Nutrients.

[123] Hu C., Yan Y., Ji F., Zhou H. (2021). Maternal Obesity increases oxidative stress in placenta and it is associated with intestinal microbiota. Front. Cell. Infect. Microbiol..

[124] Elías-López A., Vázquez-Mena O., Sferruzzi-Perri A. (2023). Mitochondrial dysfunction in the offspring of obese mothers and it’s transmission through damaged oocyte mitochondria: Integration of mechanisms. Biochim. Biophys. Acta (BBA) Mol. Basis Dis..

[125] Mandò C., Castiglioni S., Novielli C., Anelli G.M., Serati A., Parisi F., Lubrano C., Zocchi M., Ottria R., Giovarelli M. (2024). Placental bioenergetics and antioxidant homeostasis in maternal obesity and gestational diabetes. Antioxidants.

[126] de Oliveira M.P., da Silva L.E., Fernandes B.B., Steiner M.R., Pistóia D.G., Cichella T.d.S., Jacinto L.B., Spuldaro K.M., Iser B.P.M., Rezin G.T. (2025). The impact of obesity on mitochondrial dysfunction during pregnancy. Mol. Cell. Endocrinol..

[127] Zhang C.X., Candia A.A., Sferruzzi-Perri A.N. (2024). Placental inflammation, oxidative stress, and fetal outcomes in maternal obesity. Trends Endocrinol. Metab..

[128] Napso T., Lean S.C., Lu M., Mort E.J., Desforges M., Moghimi A., Bartels B., El-Bacha T., Fowden A.L., Camm E.J. (2022). Diet-induced maternal obesity impacts feto-placental growth and induces sex-specific alterations in placental morphology, mitochondrial bioenergetics, dynamics, lipid metabolism and oxidative stress in mice. Acta Physiol..

[129] Heldens A., Antwi M., Onghena L., Meese T., Gansemans Y., Smet J., Dupont E., Verhelst X., Raevens S., Van Vlierberghe H. (2025). Mitochondrial dysfunction characterises the multigenerational effects of maternal obesity on MASLD. JHEP Rep..

[130] da Cruz K.L.D.O., Salla D.H., de Oliveira M.P., da Silva L.E., Vedova L.M.D., Mendes T.F., Bressan C.B.C., Costa A.B., da Silva M.R., Réus G.Z. (2022). The impact of obesity-related neuroinflammation on postpartum depression: A narrative review. Int. J. Dev. Neurosci..

[131] Steiner M.R., de Mello A.H., Salla D.H., Bressan C.B.C., Mendes R.L., de Oliveira M.P., da Silva L.E., Fernandes B.B., Lima I.R., Zaccaron R.P. (2025). The Impact of Maternal Obesity and Deprivation On Energy Metabolism, Oxidative Stress and Brain Antioxidant Defense in the Neurodevelopment of Offspring in the Short, Medium and Long Term. Mol. Neurobiol..

[132] Lackovic M., Nikolic D., Milicic B., Dimitrijevic D., Jovanovic I., Radosavljevic S., Mihajlovic S. (2024). Pre-pregnancy obesity and infants’ motor development within the first twelve months of life: Who is expected to be the ultimate carrier of the obesity burden?. Nutrients.

[133] Rasmussen J.M., Tuulari J.J., Nolvi S., Thompson P.M., Merisaari H., Lavonius M., Karlsson L., Entringer S., Wadhwa P.D., Karlsson H. (2023). Maternal pre-pregnancy body mass index is associated with newborn offspring hypothalamic mean diffusivity: A prospective dual-cohort study. BMC Med..

[134] Rosberg A., Merisaari H., Lewis J.D., Hashempour N., Lukkarinen M., Rasmussen J.M., Scheinin N.M., Karlsson L., Karlsson H., Tuulari J.J. (2025). Associations between maternal pre-pregnancy BMI and mean diffusivity of the hippocampus and amygdala in infants. Int. J. Obes..

[135] Fileva N., Severino M., Tortora D., Ramaglia A., Paladini D., Rossi A. (2023). Second trimester fetal MRI of the brain: Through the ground glass. J. Clin. Ultrasound.

[136] Leibovitz Z., Lerman-Sagie T., Haddad L. (2022). Fetal Brain Development: Regulating Processes and Related Malformations. Life.

[137] Beretta E., Cuboni G., Deidda G. (2025). Unveiling GABA and serotonin interactions during neurodevelopment to re-open adult critical periods for neuropsychiatric disorders. Int. J. Mol. Sci..

[138] Perumal N., Manji K.P., Darling A.M., Kisenge R.R., Kvestad I., Hysing M., Belinger D.C., Urassa W., Strand T.A., Duggan C.P. (2021). gestational age, birth weight, and neurocognitive development in adolescents in tanzania. J. Pediatr..

[139] Hua J., Barnett A.L., Lin Y., Guan H., Sun Y., Williams G.J., Fu Y., Zhou Y., Du W. (2022). Association of gestational age at birth with subsequent neurodevelopment in early childhood: A national retrospective cohort study in China. Front. Pediatr..

[140] Marín M.J.B., Elena J.A.B., Clavijo J.M., López J.J., López D.M.L., Mesa E.G. (2022). Neurodevelopment outcome in children with fetal growth restriction at six years of age: A retrospective cohort study. Int. J. Environ. Res. Public Health.

[141] Jiang N., Ma S.-S., Zu P., Zhang L., Xu M., Bian J.-F., Xu J.-R., Luo W., Wang H.-X., Zhu D.-M. (2025). Intimate partner violence during pregnancy and early offspring development: A prospective birth cohort study. Biol. Psychiatry.

[142] Olsen J.E., Lee K.J., Spittle A.J., Anderson P.J., Doyle L.W., Cheong J.L.Y., the members of the Victorian Infant Collaborative Study Group (2021). The causal effect of being born extremely preterm or extremely low birthweight on neurodevelopment and social-emotional development at 2 years. Acta Paediatr..

[143] Xie Y., Xiao H., Zheng D., Mahai G., Li Y., Xia W., Xu S., Zhou A. (2025). Associations of prenatal metal exposure with child neurodevelopment and mediation by perturbation of metabolic pathways. Nat. Commun..

[144] Fan W.Q., Molinaro A. (2020). Maternal obesity adversely affects early breastfeeding in a multicultural, multi-socioeconomic Melbourne community. A. N. Z. J. Obstet. Gynaecol..

[145] Mantzorou M., Papandreou D., Vasios G.K., Pavlidou E., Antasouras G., Psara E., Taha Z., Poulios E., Giaginis C. (2022). Exclusive breastfeeding for at least four months is associated with a lower prevalence of overweight and obesity in mothers and their children after 2–5 years from delivery. Nutrients.

[146] Froń A., Orczyk-Pawiłowicz M. (2023). Understanding the immunological quality of breast milk in maternal overweight and obesity. Nutrients.

[147] Mazur D., Satora M., Rekowska A.K., Kabała Z., Łomża A., Kimber-Trojnar Ż., Leszczyńska-Gorzelak B. (2023). Influence of breastfeeding on the state of meta-inflammation in obesity—A narrative review. Curr. Issues Mol. Biol..

[148] Foster S.F., Vazquez C., Cubbin C., Nichols A.R., Rickman R.R., Widen E.M. (2023). Breastfeeding, socioeconomic status, and long-term postpartum weight retention. Int. Breastfeed. J..

[149] Yamamoto M., Takami M., Misumi T., Kawakami C., Miyagi E., Ito S., Aoki S., Japan Environment and Children’s Study (JECS) Group (2022). Effects of breastfeeding on postpartum weight change in Japanese women: The Japan Environment and Children’s Study (JECS). PLoS ONE.

[150] Calcaterra V., Cena H., Pirazzi A., Sottotetti F., Cordaro E., Cavallo C., Milanta C., El Masri D., Conti M.V., Vandoni M. (2025). From pregnancy to breastfeeding: The role of maternal exercise in preventing childhood obesity. Nutrients.

[151] Haddaway N.R., Page M.J., Pritchard C.C., McGuinness L.A. (2022). *PRISMA2020*: An R package and Shiny app for producing PRISMA 2020-compliant flow diagrams, with interactivity for optimised digital transparency and Open Synthesis. Campbell Syst. Rev..

[152] Akker O.R.v.D., Peters G.-J.Y., Bakker C.J., Carlsson R., Coles N.A., Corker K.S., Feldman G., Moreau D., Nordström T., Pickering J.S. (2023). Increasing the transparency of systematic reviews: Presenting a generalized registration form. Syst. Rev..

[153] Alba-Linares J.J., Pérez R.F., Tejedor J.R., Bastante-Rodríguez D., Ponce F., Carbonell N.G., Zafra R.G., Fernández A.F., Fraga M.F., Lurbe E. (2023). Maternal obesity and gestational diabetes reprogram the methylome of offspring beyond birth by inducing epigenetic signatures in metabolic and developmental pathways. Cardiovasc. Diabetol..

[154] Álvarez-Bueno C., Cavero-Redondo I., la Cruz L.L.-D., Notario-Pacheco B., Martínez-Vizcaíno V. (2017). Association between pre-pregnancy overweight and obesity and children’s neurocognitive development: A systematic review and meta-analysis of observational studies. Leuk. Res..

[155] Alves J.M., Luo S., Chow T., Herting M., Xiang A.H., Page K.A. (2020). Sex differences in the association between prenatal exposure to maternal obesity and hippocampal volume in children. Brain Behav..

[156] Baker P.R., Patinkin Z., Shapiro A.L., De La Houssaye B.A., Woontner M., Boyle K.E., Vanderlinden L., Dabelea D., Friedman J.E. (2017). Maternal obesity and increased neonatal adiposity correspond with altered infant mesenchymal stem cell metabolism. J. Clin. Investig..

[157] Basatemur E., Gardiner J., Williams C., Melhuish E., Barnes J., Sutcliffe A. (2013). Maternal Prepregnancy BMI and Child Cognition: A Longitudinal Cohort Study. Pediatrics.

[158] Bauer C.C.C., Moreno B., González-Santos L., Concha L., Barquera S., Barrios F.A. (2014). Child overweight and obesity are associated with reduced executive cognitive performance and brain alterations: A magnetic resonance imaging study in Mexican children. Pediatr. Obes..

[159] Boyle K.E., Patinkin Z.W., Shapiro A.L., Bader C., Vanderlinden L., Kechris K., Janssen R.C., Ford R.J., Smith B.K., Steinberg G.R. (2017). Maternal obesity alters fatty acid oxidation, AMPK activity, and associated DNA methylation in mesenchymal stem cells from human infants. Mol. Metab..

[160] van der Burg J.W., Sen S., Chomitz V.R., Seidell J.C., Leviton A., Dammann O. (2015). The role of systemic inflammation linking maternal BMI to neurodevelopment in children. Pediatr. Res..

[161] Buss C., Entringer S., Davis E.P., Hobel C.J., Swanson J.M., Wadhwa P.D., Sandman C.A. (2012). Impaired Executive Function Mediates the Association between Maternal Pre-Pregnancy Body Mass Index and Child ADHD Symptoms. PLoS ONE.

[162] Buss C., Tuulari J.J., Nolvi S., Thompson P.M., Merisaari H., Lavonius M., Karlsson L., Gyllenhammer L.E., Lindsay K.L., O’Connor T.G. (2024). Intergenerational transmission of obesity risk by fetal programming of hypothalamus development. Psychoneuroendocrinology.

[163] Cáceres A., Carreras-Gallo N., Andrusaityte S., Bustamante M., Carracedo Á., Chatzi L., Dwaraka V.B., Grazuleviciene R., Gutzkow K.B., Lepeule J. (2023). Prenatal environmental exposures associated with sex differences in childhood obesity and neurodevelopment. BMC Med..

[164] Camargos A.C.R., Mendonça V.A., Oliveira K.S., de Andrade C.A., Leite H.R., da Fonseca S.F., Vieira E.L.M., Júnior A.L.T., Lacerda A.C.R. (2017). Association between obesity-related biomarkers and cognitive and motor development in infants. Behav. Brain Res..

[165] Casas M., Chatzi L., Carsin A.-E., Amiano P., Guxens M., Kogevinas M., Koutra K., Lertxundi N., Murcia M., Rebagliato M. (2013). Maternal pre-pregnancy overweight and obesity, and child neuropsychological development: Two Southern European birth cohort studies. Leuk. Res..

[166] Project O.B.O.T.I., Casas M., Forns J., Martínez D., Guxens M., Fernandez-Somoano A., Ibarluzea J., Lertxundi N., Murcia M., Rebagliato M. (2017). Maternal pre-pregnancy obesity and neuropsychological development in pre-school children: A prospective cohort study. Pediatr. Res..

[167] Cirulli F., De Simone R., Musillo C., Ajmone-Cat M.A., Berry A. (2022). Inflammatory Signatures of Maternal Obesity as Risk Factors for Neurodevelopmental Disorders: Role of Maternal Microbiota and Nutritional Intervention Strategies. Nutrients.

[168] Cirulli F., Musillo C., Berry A. (2020). Maternal Obesity as a Risk Factor for Brain Development and Mental Health in the Offspring. Neuroscience.

[169] Dearden L., Ozanne S.E. (2015). Early life origins of metabolic disease: Developmental programming of hypothalamic pathways controlling energy homeostasis. Front. Neuroendocr..

[170] Dearden L., Buller S., Furigo I., Fernandez-Twinn D., Ozanne S. (2020). Maternal obesity causes fetal hypothalamic insulin resistance and disrupts development of hypothalamic feeding pathways. Mol. Metab..

[171] Desai M., Han G., Ross M.G. (2016). Programmed hyperphagia in offspring of obese dams: Altered expression of hypothalamic nutrient sensors, neurogenic factors and epigenetic modulators. Appetite.

[172] Duko B., Mengistu T.S., Stacey D., Moran L.J., Tessema G., Pereira G., Bedaso A., Gebremedhin A.T., Alati R., Ayonrinde O.T. (2024). Associations between maternal preconception and pregnancy adiposity and neuropsychiatric and behavioral outcomes in the offspring: A systematic review and meta-analysis. Psychiatry Res..

[173] Edlow A.G., Vora N.L., Hui L., Wick H.C., Cowan J.M., Bianchi D.W. (2014). Maternal Obesity Affects Fetal Neurodevelopmental and Metabolic Gene Expression: A Pilot Study. PLoS ONE.

[174] Edlow A.G., Guedj F., Pennings J.L., Sverdlov D., Neri C., Bianchi D.W. (2016). Males are from Mars, and females are from Venus: Sex-specific fetal brain gene expression signatures in a mouse model of maternal diet-induced obesity. Am. J. Obstet. Gynecol..

[175] Fernandes C., Grayton H., Poston L., Samuelsson A.-M., Taylor P.D., Collier D.A., Rodriguez A. (2011). Prenatal exposure to maternal obesity leads to hyperactivity in offspring. Mol. Psychiatry.

[176] Francis E.C., Kechris K., Jansson T., Dabelea D., Perng W. (2023). Novel Metabolic Subtypes in Pregnant Women and Risk of Early Childhood Obesity in Offspring. JAMA Netw. Open.

[177] Fuemmeler B.F., Zucker N., Sheng Y., Sanchez C.E., Maguire R., Murphy S.K., Kollins S.H., Hoyo C. (2019). Pre-Pregnancy Weight and Symptoms of Attention Deficit Hyperactivity Disorder and Executive Functioning Behaviors in Preschool Children. Int. J. Environ. Res. Public Health.

[178] Furigo I.C., Dearden L. (2022). Mechanisms mediating the impact of maternal obesity on offspring hypothalamic development and later function. Front. Endocrinol..

[179] Gaillard R., Rifas-Shiman S.L., Perng W., Oken E., Gillman M.W. (2016). Maternal inflammation during pregnancy and childhood adiposity. Obesity.

[180] Galley J.D., Bailey M., Dush C.K., Schoppe-Sullivan S., Christian L.M. (2014). Maternal Obesity Is Associated with Alterations in the Gut Microbiome in Toddlers. PLoS ONE.

[181] Grissom N.M., Herdt C.T., Desilets J., Lidsky-Everson J., Reyes T.M. (2014). Dissociable Deficits of Executive Function Caused by Gestational Adversity are Linked to Specific Transcriptional Changes in the Prefrontal Cortex. Neuropsychopharmacology.

[182] Guzzardi M.A., Ederveen T.H., Rizzo F., Weisz A., Collado M.C., Muratori F., Gross G., Alkema W., Iozzo P. (2022). Maternal pre-pregnancy overweight and neonatal gut bacterial colonization are associated with cognitive development and gut microbiota composition in pre-school-age offspring. Brain Behav. Immun..

[183] Harmancıoğlu B., Kabaran S. (2023). Maternal high fat diets: Impacts on offspring obesity and epigenetic hypothalamic programming. Front. Genet..

[184] Hasegawa Y., Zhang Z., Taha A.Y., Capitanio J.P., Bauman M.D., Golub M.S., Van de Water J., VandeVoort C.A., Walker C.K., Slupsky C.M. (2022). Impact of Maternal Obesity on the Gestational Metabolome and Infant Metabolome, Brain, and Behavioral Development in Rhesus Macaques. Metabolites.

[185] Hinkle S.N., Schieve L.A., Stein A.D., Swan D.W., Ramakrishnan U., Sharma A.J. (2012). Associations between maternal prepregnancy body mass index and child neurodevelopment at 2 years of age. Int. J. Obes..

[186] Huang L., Yu X., Keim S., Li L., Zhang L., Zhang J. (2014). Maternal prepregnancy obesity and child neurodevelopment in the Collaborative Perinatal Project. Leuk. Res..

[187] Keimpema E., Calvigioni D., Harkany T. (2013). Endocannabinoid signals in the developmental programming of delayed-onset neuropsychiatric and metabolic illnesses. Biochem. Soc. Trans..

[188] Kim T.-W., Park H.-S. (2018). Physical exercise improves cognitive function by enhancing hippocampal neurogenesis and inhibiting apoptosis in male offspring born to obese mother. Behav. Brain Res..

[189] Krakowiak P., Walker C.K., Bremer A.A., Baker A.S., Ozonoff S., Hansen R.L., Hertz-Picciotto I. (2012). Maternal Metabolic Conditions and Risk for Autism and Other Neurodevelopmental Disorders. Pediatrics.

[190] Krzeczkowski J.E., Boylan K., Arbuckle T.E., Dodds L., Muckle G., Fraser W., Favotto L.A., Van Lieshout R.J. (2018). Neurodevelopment in 3–4 year old children exposed to maternal hyperglycemia or adiposity in utero. Early Hum. Dev..

[191] Lee J.Y., Lee H.J., Jang Y.H., Kim H., Im K., Yang S., Hoh J.-K., Ahn J.-H. (2023). Maternal pre-pregnancy obesity affects the uncinate fasciculus white matter tract in preterm infants. Front. Pediatr..

[192] Levin B.E. (2010). Interaction of perinatal and pre-pubertal factors with genetic predisposition in the development of neural pathways involved in the regulation of energy homeostasis. Brain Res..

[193] Li X., Andres A., Shankar K., Pivik R.T., Glasier C.M., Ramakrishnaiah R.H., Zhang Y., Badger T.M., Ou X. (2016). Differences in brain functional connectivity at resting state in neonates born to healthy obese or normal-weight mothers. Int. J. Obes..

[194] Lippert R.N., Brüning J.C. (2022). Maternal Metabolic Programming of the Developing Central Nervous System: Unified Pathways to Metabolic and Psychiatric Disorders. Biol. Psychiatry.

[195] Liu X., Li X., Xia B., Jin X., Zou Q., Zeng Z., Zhao W., Yan S., Li L., Yuan S. (2021). High-fiber diet mitigates maternal obesity-induced cognitive and social dysfunction in the offspring via gut-brain axis. Cell Metab..

[196] Luo S., Angelo B., Chow T., Monterosso J.R., Xiang A.H., Thompson P.M., Page K.A. (2021). The role of maternal BMI on brain food cue reactivity in children: A preliminary study. Brain Imaging Behav..

[197] Menting M.D., van de Beek C., de Rooij S.R., Painter R.C., Vrijkotte T.G., Roseboom T.J. (2018). The association between pre-pregnancy overweight/obesity and offspring’s behavioral problems and executive functioning. Early Hum. Dev..

[198] Mina T.H., Lahti M., Drake A.J., Denison F.C., Räikkönen K., Norman J.E., Reynolds R.M. (2017). Prenatal exposure to maternal very severe obesity is associated with impaired neurodevelopment and executive functioning in children. Pediatr. Res..

[199] Mina T.H., Lahti M., Drake A.J., Räikkönen K., Minnis H., Denison F.C., Norman J.E., Reynolds R.M. (2016). Prenatal exposure to very severe maternal obesity is associated with adverse neuropsychiatric outcomes in children. Psychol. Med..

[200] Monthé-Drèze C., Rifas-Shiman S.L., Gold D.R., Oken E., Sen S. (2018). Maternal obesity and offspring cognition: The role of inflammation. Pediatr. Res..

[201] Morgan J.E., Lee S.S., Mahrer N.E., Guardino C.M., Davis E.P., Shalowitz M.U., Ramey S.L., Schetter C.D. (2020). Prenatal maternal C-reactive protein prospectively predicts child executive functioning at ages 4–6 years. Dev. Psychobiol..

[202] Na X., Phelan N., Tadros M., Wu Z., Andres A., Badger T., Glasier C., Ramakrishnaiah R., Rowell A., Wang L. (2021). Maternal Obesity during Pregnancy is Associated with Lower Cortical Thickness in the Neonate Brain. Am. J. Neuroradiol..

[203] Ou X., Thakali K.M., Shankar K., Andres A., Badger T.M. (2015). Maternal adiposity negatively influences infant brain white matter development. Obesity.

[204] Page K.A., Luo S., Wang X., Chow T., Alves J., Buchanan T.A., Xiang A.H. (2019). Children Exposed to Maternal Obesity or Gestational Diabetes Mellitus During Early Fetal Development Have Hypothalamic Alterations That Predict Future Weight Gain. Diabetes Care.

[205] Panagos P.G., Vishwanathan R., Penfield-Cyr A., Matthan N.R., Shivappa N., Wirth M.D., Hebert J.R., Sen S. (2016). Breastmilk from obese mothers has pro-inflammatory properties and decreased neuroprotective factors. J. Perinatol..

[206] Park S., Jang A., Bouret S.G. (2020). Maternal obesity-induced endoplasmic reticulum stress causes metabolic alterations and abnormal hypothalamic development in the offspring. PLoS Biol..

[207] Parsaei M., Hashemi S.M., Moghaddam H.S., Peterson B.S. (2024). A systematic review of MRI studies on the effects of maternal obesity on offspring brain structure and function. J. Neurosci. Res..

[208] Plucińska K., Barger S.W. (2018). Maternal obesity reprograms offspring’s executive brain centers in a sex-specific manner?. J. Neurochem..

[209] Rafiq T., Stearns J.C., Shanmuganathan M., Azab S.M., Anand S.S., Thabane L., Beyene J., Williams N.C., Morrison K.M., Teo K.K. (2023). Integrative multiomics analysis of infant gut microbiome and serum metabolome reveals key molecular biomarkers of early onset childhood obesity. Heliyon.

[210] Ross M.G., Desai M. (2014). Developmental Programming of Appetite/Satiety. Ann. Nutr. Metab..

[211] Salzwedel A.P., Gao W., Andres A., Badger T.M., Glasier C.M., Ramakrishnaiah R.H., Rowell A.C., Ou X. (2019). Maternal Adiposity Influences Neonatal Brain Functional Connectivity. Front. Hum. Neurosci..

[212] Samara A., Murphy T., Strain J., Rutlin J., Sun P., Neyman O., Sreevalsan N., Shimony J.S., Ances B.M., Song S.-K. (2020). Neuroinflammation and White Matter Alterations in Obesity Assessed by Diffusion Basis Spectrum Imaging. Front. Hum. Neurosci..

[213] Sanchez C.E., Barry C., Sabhlok A., Russell K., Majors A., Kollins S.H., Fuemmeler B.F. (2017). Maternal pre-pregnancy obesity and child neurodevelopmental outcomes: A meta-analysis. Obes. Rev..

[214] Sanders T.R., Kim D.W., Glendining K.A., Jasoni C.L. (2014). Maternal obesity and IL-6 lead to aberrant developmental gene expression and deregulated neurite growth in the fetal arcuate nucleus. Endocrinology.

[215] Sanguinetti E., Guzzardi M.A., Tripodi M., Panetta D., Selma-Royo M., Zega A., Telleschi M., Collado M.C., Iozzo P. (2019). Microbiota signatures relating to reduced memory and exploratory behaviour in the offspring of overweight mothers in a murine model. Sci. Rep..

[216] Sarker G., Peleg-Raibstein D. (2018). Maternal Overnutrition Induces Long-Term Cognitive Deficits across Several Generations. Nutrients.

[217] Saros L., Lind A., Setänen S., Tertti K., Koivuniemi E., Ahtola A., Haataja L., Shivappa N., Hébert J.R., Vahlberg T. (2023). Maternal obesity, gestational diabetes mellitus, and diet in association with neurodevelopment of 2-year-old children. Pediatr. Res..

[218] Schmidt R.J., Liang D., Busgang S.A., Curtin P., Giulivi C. (2021). Maternal Plasma Metabolic Profile Demarcates a Role for Neuroinflammation in Non-Typical Development of Children. Metabolites.

[219] Shapiro A.L.B., Moore B.F., Sutton B., Wilkening G., Stence N., Dabelea D., Tregellas J.R. (2020). In Utero Exposure to Maternal Overweight or Obesity is Associated with Altered Offspring Brain Function in Middle Childhood. Obesity.

[220] Skowronski A.A., Leibel R.L., LeDuc C.A. (2023). Neurodevelopmental Programming of Adiposity: Contributions to Obesity Risk. Endocr. Rev..

[221] Stachowiak E.K., Oommen S., Vasu V.T., Srinivasan M., Stachowiak M., Gohil K., Patel M.S. (2013). Maternal obesity affects gene expression and cellular development in fetal brains. Nutr. Neurosci..

[222] Sullivan E.L., Riper K.M., Lockard R., Valleau J.C. (2015). Maternal high-fat diet programming of the neuroendocrine system and behavior. Horm. Behav..

[223] Tanda R., Salsberry P.J., Reagan P.B., Fang M.Z. (2012). The Impact of Prepregnancy Obesity on Children’s Cognitive Test Scores. Matern. Child Health J..

[224] Torres-Espinola F.J., Berglund S.K., García-Valdés L.M., Segura M.T., Jerez A., Campos D., Moreno-Torres R., Rueda R., Catena A., Pérez-García M. (2015). Maternal Obesity, Overweight and Gestational Diabetes Affect the Offspring Neurodevelopment at 6 and 18 Months of Age—A Follow Up from the PREOBE Cohort. PLoS ONE.

[225] Urbonaite G., Knyzeliene A., Bunn F.S., Smalskys A., Neniskyte U. (2022). The impact of maternal high-fat diet on offspring neurodevelopment. Front. Neurosci..

[226] Walker C., Naef L., D’Asti E., Long H., Xu Z., Moreau A., Azeddine B. (2008). Perinatal Maternal Fat Intake Affects Metabolism and Hippocampal Function in the Offspring. Ann. N. Y. Acad. Sci..

[227] Widen E.M., Nichols A.R., Kahn L.G., Factor-Litvak P., Insel B.J., Hoepner L., Dube S.M., Rauh V., Perera F., Rundle A. (2019). Prepregnancy obesity is associated with cognitive outcomes in boys in a low-income, multiethnic birth cohort. BMC Pediatr..

[228] Wu T., Deng S., Li W.-G., Yu Y., Li F., Mao M. (2013). Maternal Obesity Caused by Overnutrition Exposure Leads to Reversal Learning Deficits and Striatal Disturbance in Rats. PLoS ONE.

[229] Yeung E.H., Sundaram R., Ghassabian A., Xie Y., Louis G.B. (2017). Parental Obesity and Early Childhood Development. Pediatrics.

[230] Zhu C., Han T.-L., Zhao Y., Zhou X., Mao X., Qi H., Baker P.N., Zhang H. (2018). A mouse model of pre-pregnancy maternal obesity combined with offspring exposure to a high-fat diet resulted in cognitive impairment in male offspring. Exp. Cell Res..

[231] Halkiopoulos C., Gkintoni E. (2025). The Role of Machine Learning in AR/VR-Based Cognitive Therapies: A Systematic Review for Mental Health Disorders. Electronics.

[232] Gkintoni E., Vassilopoulos S.P., Nikolaou G. (2025). Mindfulness-Based Cognitive Therapy in Clinical Practice: A Systematic Review of Neurocognitive Outcomes and Applications for Mental Health and Well-Being. J. Clin. Med..

[233] Stephenson J., Heslehurst N., Hall J., Schoenaker D.A.J.M., Hutchinson J., Cade J.E., Poston L., Barrett G., Crozier S.R., Barker M. (2018). Before the beginning: Nutrition and lifestyle in the preconception period and its importance for future health. Lancet.

[234] Miguel-Andrés I., Huerta-Franco M.R., García-González S.B., León-Rodríguez M., Barrera-Beltrán K., Ortiz-Lango L.A. (2023). The Importance of the Kinematic Evaluation Methods of the Upper Limbs in Women with Breast Cancer Mastectomy: A Literature Review. Healthcare.

[235] Crawford A., Serhal E. (2020). Digital Health Equity and COVID-19: The Innovation Curve Cannot Reinforce the Social Gradient of Health. J. Med. Internet Res..

[236] Gkintoni E., Vassilopoulos S.P., Nikolaou G. (2025). Next-Generation Cognitive-Behavioral Therapy for Depression: Integrating Digital Tools, Teletherapy, and Personalization for Enhanced Mental Health Outcomes. Medicina.

[237] Gkintoni E., Vantaraki F., Skoulidi C., Anastassopoulos P., Vantarakis A. (2024). Gamified Health Promotion in Schools: The Integration of Neuropsychological Aspects and CBT—A Systematic Review. Medicina.

[238] Patel M.L., Hopkins C.M., Brooks T.L., Bennett G.G. (2019). Comparing Self-Monitoring Strategies for Weight Loss in a Smartphone App: Randomized Controlled Trial. JMIR mHealth uHealth.

[239] Gkintoni E., Vantaraki F., Skoulidi C., Anastassopoulos P., Vantarakis A. (2024). Promoting Physical and Mental Health among Children and Adolescents via Gamification—A Conceptual Systematic Review. Behav. Sci..

[240] Daly L.M., Horey D., Middleton P.F., Boyle F.M., Flenady V. (2018). The Effect of Mobile App Interventions on Influencing Healthy Maternal Behavior and Improving Perinatal Health Outcomes: Systematic Review. JMIR MHealth UHealth.

[241] Johnson D., Deterding S., Kuhn K.-A., Staneva A., Stoyanov S., Hides L. (2016). Gamification for health and wellbeing: A systematic review of the literature. Internet Interv..

[242] Page M.J., McKenzie J.E., Bossuyt P.M., Boutron I., Hoffmann T.C., Mulrow C.D., Shamseer L., Tetzlaff J.M., Aki E.A., Brennan S.E. (2021). The PRISMA 2020 statement: An updated guideline for reporting systematic reviews. BMJ.

