# Supplementary material for: Neurodevelopmental Pathways from Maternal Obesity to Offspring Outcomes: An Umbrella Review of Cognitive and Behavioral Consequences Across Development
Source: Healthcare (Basel). 2025 Oct 21;13(20):2653. doi: 10.3390/healthcare13202653 (PMC12563868; doi:10.3390/healthcare13202653)
Supplement: Supplementary file 1 [file healthcare-13-02653-s001.zip › Table_S4.pdf]

Supplement Table S4. Summary of Domain-Specific Neurodevelopmental Outcomes Associated with Maternal Obesity

Domain	Specific Outcomes	Effect Size Range†	Number of Studies‡	Quality of Evidence§
**COGNITIVE FUNCTION**				
General Intelligence	Full-scale IQ reduction	-2.7 to -4.1 points	23	Moderate
	Verbal IQ reduction	-3.8 to -5.4 points	18	Moderate
	Performance IQ reduction	-1.9 to -3.1 points	15	Moderate
Language Development	Vocabulary (PPVT) delays	-3.5 to -8.0 points	18	High
	Expressive language delays	4-6 month delays	14	High
	Reading comprehension	SMD -0.33 (CI: -0.52, -0.14)	12	Moderate
Processing Speed	Cognitive processing	SMD -0.18 to -0.28	11	Low-Moderate
**EXECUTIVE FUNCTION**				
Working Memory	Verbal working memory	SMD -0.36 (CI: -0.53, -0.19)	13	High
	Spatial working memory	SMD -0.22 (CI: -0.38, -0.06)	11	Moderate
Inhibitory Control	Response inhibition	SMD -0.41 (CI: -0.59, -0.23)	12	High
	Interference control	SMD -0.39 (CI: -0.56, -0.22)	10	High
Attention	Sustained attention	SMD -0.44 (CI: -0.62, -0.26)	15	High
	Selective attention	SMD -0.38 (CI: -0.57, -0.19)	13	High
Cognitive Flexibility	Set-shifting abilities	SMD -0.29 (CI: -0.46, -0.12)	9	Moderate
**BEHAVIORAL OUTCOMES**				
ADHD Symptoms	Hyperactivity	OR 1.62 (CI: 1.45, 1.81)	22	Very High
	Inattention	OR 1.47 (CI: 1.32, 1.64)	22	High
	Impulsivity	OR 1.58 (CI: 1.41, 1.77)	22	High
	Combined type ADHD	OR 1.73 (CI: 1.52, 1.97)	22	Very High
Anxiety Disorders	General anxiety symptoms	OR 1.34 (CI: 1.18, 1.52)	16	Moderate
	Social anxiety	OR 1.48 (CI: 1.27, 1.73)	12	Moderate

Depression	Depressive symptoms (adolescence)	OR 1.29 (CI: 1.11, 1.50)	12	Moderate
Emotional Regulation	Dysregulation symptoms	OR 1.50-2.20	14	Moderate
	Emotion recognition deficits	SMD -0.31 (CI: -0.48, -0.14)	11	Moderate
Externalizing Behaviors	Aggression	OR 1.51 (CI: 1.33, 1.71)	17	High
	Oppositional behavior	OR 1.43 (CI: 1.26, 1.62)	15	High
Social Functioning	Social problem-solving	SMD -0.28 (CI: -0.45, -0.11)	9	Low-Moderate
**AUTISM SPECTRUM**				
ASD Diagnosis	Males	OR 1.54 (CI: 1.28, 1.86)	11	Moderate
	Females	OR 1.23 (CI: 0.98, 1.54)	11	Moderate
	Overall risk	OR 1.30-1.70	11	Moderate

Abbreviations: ADHD, attention deficit hyperactivity disorder; ASD, autism spectrum disorder; CI, confidence interval; OR, odds ratio; PPVT, Peabody Picture Vocabulary Test; SMD, standardized mean difference.

Footnotes:

†Effect Size Interpretation:

- For continuous outcomes (IQ points, delays): Negative values indicate impairment; point differences represent actual score reductions on standardized assessments
- For standardized mean differences (SMD): Values represent standard deviation units (0.2 = small, 0.5 = medium, 0.8 = large effect by Cohen's criteria)
- For odds ratios (OR): Values >1.0 indicate increased risk; OR 1.5 = 50% increased risk, OR 2.0 = 100% increased risk (doubled risk)
- All effect sizes compare maternal obesity (BMI ≥30 kg/m²) versus normal weight (BMI 18.5-24.9 kg/m²) reference groups
- Ranges represent variation across studies, age groups, and assessment methods
- 95% confidence intervals (CI) provided where available from meta-analyses

‡Number of Studies:

- Represents total number of studies examining each specific outcome domain

- Many studies assessed multiple outcomes within a domain
- Studies include prospective cohorts, longitudinal designs, and cross-sectional analyses
- Sample sizes ranged from n=88 to n=19,517 mother-child pairs per study
- Total unique participants across all studies: >650,000 mother-child pairs

§**Quality of Evidence Rating:**

- **Very High:** Consistent findings across ≥20 high-quality studies with low risk of bias; minimal heterogeneity; dose-response relationships demonstrated; multiple populations studied
- **High:** Consistent findings across multiple (10-19) high-quality studies with low-moderate risk of bias; some heterogeneity explained by methodological factors; replicated across populations
- **Moderate:** Generally consistent findings with some heterogeneity or methodological limitations; adequate number of studies (5-9) but variable quality; findings replicated in some but not all populations
- **Low-Moderate:** Limited number of studies (3-7) with mixed quality; some inconsistency in findings; preliminary evidence requiring further investigation
- **Low:** Few studies (<5) available; inconsistent findings; significant methodological concerns; or evidence primarily from single population

Clinical Significance: Effect sizes were evaluated for clinical meaningfulness using established criteria:

- **IQ reductions of 2.5+ points:** Meaningful at population level; 4+ points approach individual clinical significance
- **SMD ≥0.3:** Considered clinically meaningful for functional outcomes
- **OR ≥1.4:** Indicates substantial increased risk with population health implications
- **Combined ADHD OR ≥1.7:** Represents large clinical and public health impact given high prevalence
