# Supplementary material for: Neurodevelopmental Pathways from Maternal Obesity to Offspring Outcomes: An Umbrella Review of Cognitive and Behavioral Consequences Across Development
Source: Healthcare (Basel). 2025 Oct 21;13(20):2653. doi: 10.3390/healthcare13202653 (PMC12563868; doi:10.3390/healthcare13202653)
Supplement: Supplementary file 1 [file healthcare-13-02653-s001.zip › Table_S2_Abbreviation List.pdf]

Table S2. Abbreviation List

Biological Molecules and Markers

- AMPK - AMP-activated protein kinase
- APOD - Apolipoprotein D
- BCL2 - B-cell lymphoma 2 (anti-apoptotic gene)
- BDNF - Brain-derived neurotrophic factor
- CASP9 - Caspase 9 (pro-apoptotic gene)
- CRP - C-reactive protein
- DHA - Docosahexaenoic acid
- DNA - Deoxyribonucleic acid
- DNMT1 - DNA methylase
- EPA - Eicosapentaenoic acid
- FFA - Free fatty acids
- FOS - Fos proto-oncogene
- IGF - Insulin-like growth factor
- IFN - Interferon
- IL - Interleukin
- IL-1β - Interleukin-1 beta
- IL-6 - Interleukin-6
- IL-8 - Interleukin-8
- IL-10 - Interleukin-10
- IL-12 - Interleukin-12
- IL-13 - Interleukin-13
- IL-15 - Interleukin-15
- IL-17A - Interleukin-17A
- IL-2 - Interleukin-2
- mRNA - Messenger ribonucleic acid
- NF-κB - Nuclear factor-kappa B
- NPY - Neuropeptide Y
- PI3K - Phosphoinositide 3-kinase
- POMC - Pro-opiomelanocortin
- PPARA - Peroxisome proliferator-activated receptor alpha
- PUFA - Polyunsaturated fatty acids
- RNA - Ribonucleic acid
- ROS - Reactive oxygen species
- SIRT1 - Sirtuin 1
- STAT3 - Signal transducer and activator of transcription 3
- sTNFR1 - Soluble tumor necrosis factor receptor 1
- TGF - Transforming growth factor
- TNF - Tumor necrosis factor
- TNF-α - Tumor necrosis factor-alpha

Neurotransmitter Systems & Receptors

- CNR1 - Cannabinoid receptor 1
- COMT - Catechol-O-methyltransferase
- DAT - Dopamine transporter
- ESR1/ESR2 - Estrogen receptor 1/2
- GABA - Gamma-aminobutyric acid
- GABAergic - Gamma-aminobutyric acid-ergic
- HDAC1 - Histone deacetylase 1
- LTP - Long-term potentiation
- NMDA - N-methyl-d-aspartate
- OPRD1 - Opioid receptor delta 1
- Cell Types and Tissues
- MSC - Mesenchymal stem cells
- NW-MSC - Normal weight mothers' MSC
- Ob-MSC - Obese-derived mesenchymal stem cells
- PBMC - Peripheral blood mononuclear cells
- uMSC - Umbilical cord-derived mesenchymal stem cells

Anatomical and Physiological Systems

- ACC - Anterior cingulate cortex
- ARC - Arcuate nucleus
- CA1-4 - Cornu Ammonis regions 1-4 (hippocampus)

CNS - Central Nervous System
DG - Dentate gyrus
HPA - Hypothalamic-pituitary-adrenal
PFC - Prefrontal cortex
PVN - Paraventricular nucleus
STG - Superior temporal gyrus
UNC - Uncinate fasciculus
VMN - Ventromedial nucleus

Clinical and Medical Terms

ADHD - Attention Deficit Hyperactivity Disorder
ADHD-DSM-IV - ADHD symptoms (DSM-IV criteria)
AGA - Appropriate for gestational age
ASD - Autism spectrum disorder
BMI - Body Mass Index
BP - Blood pressure
DD - Developmental delays
DII - Dietary inflammatory index
DIO - Diet-induced obesity
DOHaD - Developmental Origins of Health and Disease
DR - Diet-resistant
GDM - Gestational diabetes mellitus
GWG - Gestational weight gain
SGA - Small for gestational age
T2DM - Type 2 diabetes mellitus

Neuroimaging and Assessment Tools

DBSI - Diffusion Basis Spectrum Imaging
DTI - Diffusion tensor imaging
DXA - Dual-energy X-ray absorptiometry
FA - Fractional anisotropy
fMRI - Functional magnetic resonance imaging
MRI - Magnetic resonance imaging
PET - Positron emission tomography
TBSS - Tract-based Spatial Statistics

Cognitive/Behavioral Assessment Tools

ADI - Autism Diagnostic Interview
ADOS - Autism Diagnostic Observation Schedule
ASQ - Ages and Stages Questionnaire
BASC - Behavior Assessment System for Children
BRIEF - Behavior Rating Inventory of Executive Function
BRIEF-P - Behavior Rating Inventory of Executive Function-Preschool
BSID/BSID-III - Bayley Scales of Infant Development (Third Edition)
CBCL - Child Behavior Checklist
CSHQ - Children's Sleep Habits Questionnaire
DAS - Differential Ability Scales
ESSENCE-Q - Early Symptomatic Syndrome Eliciting Neurodevelopmental Clinical Examination Questionnaire
HINE - Hammersmith Infant Neurological Examination
HTKS - Head-Toe-Knee-Shoulder task
IQ - Intelligence Quotient
K-ABC/KABC - Kaufman Assessment Battery for Children
KBIT - Kaufman Brief Intelligence Test
MABC - Movement Assessment Battery for Children
MDI - Mental Development Index
MSEL - Mullen Scales of Early Learning
NBAS - Neonatal Behavioral Assessment Scale
PDI - Psychomotor Development Index
PIAT - Peabody Individual Achievement Test
PPVT/PPVT-III - Peabody Picture Vocabulary Test (Third Edition)
SDQ - Strengths and Difficulties Questionnaire
SRS - Social Responsiveness Scale
TONI - Test of Nonverbal Intelligence
VABS - Vineland Adaptive Behavior Scales
WAIS - Wechsler Adult Intelligence Scale
WISC/WISC-IV - Wechsler Intelligence Scale for Children (Fourth Edition)

WPPSI - Wechsler Preschool and Primary Scale of Intelligence
WRAML - Wide Range Assessment of Memory and Learning
WRAVMA - Wide Range Assessment of Visual Motor Abilities

Molecular Biology Terms

BMIQ - Beta-Mixture Quantile (normalization method)
DMPs - Differentially methylated positions
DMRs - Differentially methylated regions
FAO - Fatty acid oxidation
PCR - Polymerase chain reaction
qPCR - Quantitative PCR
qRT-PCR - Quantitative Real-Time Polymerase Chain Reaction
RNA-Seq - RNA Sequencing
TLR - Toll-like receptor
UTR - Untranslated region

Study Cohorts and Projects

ABCD - Adolescent Brain Cognitive Development Study
ALSPAC - Avon Longitudinal Study of Parents and Children
BIB - Born in Bradford
CHAMACOS - Center for the Health Assessment of Mothers and Children of Salinas
CHARGE - Childhood Autism Risks from Genetics and Environment
CCCEH - Columbia Center for Children's Environmental Health
CCHN - Community Child Health Network
CHOP - Children's Hospital of Philadelphia
ECLS/ECLS-B - Early Childhood Longitudinal Study (Birth Cohort)
EDEN - Étude des Déterminants pré et postnatals du développement et de la santé de l'Enfant
ELGAN - Extremely Low Gestational Age Newborn
GUSTO - Growing Up in Singapore Towards healthy Outcomes
HAPO - Hyperglycemia and Adverse Pregnancy Outcome
HELIX - Human Early Life Exposome
HOME - Health Outcomes and Measures of the Environment
INMA - INfancia y Medio Ambiente
KPSC - Kaiser Permanente Southern California
MARBLES - Markers of Autism Risk in Babies-Learning Early Signs
MCS - Millennium Cohort Study
MoBa - Norwegian Mother, Father and Child Cohort Study
NEST - Newborn Epigenetics Study
NHS - Nurses' Health Study
NICHD - National Institute of Child Health and Human Development
PELAGIE - Perturbateurs Endocriniens: Étude Longitudinale sur les Anomalies de la Grossesse, l'Infertilité et l'Enfance
PING - Pediatric Imaging, Neurocognition, and Genetics
PISAC - Pisa birth Cohort
PREOBE - Prevention of Obesity in Early Life
Project Viva - Cohort study
RANN - Research on Autism and Neurodevelopment Network
RHEA - Greek birth cohort
START - SouTh Asian biRth cohorT

Statistical and Methodological Terms

ANOVA - Analysis of Variance
β - Beta coefficient
CI - Confidence Interval
ES - Effect Size
FDR - False discovery rate
GLM - General Linear Model
HR - Hazard Ratio
ICC - Intraclass Correlation Coefficient
IPW - Inverse probability weighting
IQR - Interquartile Range
OR - Odds Ratio
PCA - Principal Component Analysis
RR - Relative Risk
SD - Standard Deviation
SE - Standard Error

SES - Socioeconomic status
SMD - Standardized Mean Difference

Research Methodology & Databases

CINAHL - Cumulative Index to Nursing and Allied Health Literature
EMBASE - Excerpta Medica Database
GRADE - Grading of Recommendations, Assessment, Development and Evaluations
MAR - Missing at random
MEDLINE - Medical Literature Analysis and Retrieval System Online
MeSH - Medical Subject Headings
MSigDB - Molecular Signatures Database
OSF - Open Science Framework
PRISMA - Preferred Reporting Items for Systematic Reviews and Meta-Analyses
PsycINFO - Psychology Information database
REDCap - Research Electronic Data Capture
RoB - Risk of Bias
SYRCLE - Systematic Review Centre for Laboratory animal Experimentation

Body Composition/Metabolic Measures

BF - Body fat
BFM - Body fat mass
BMIAUC - BMI area under the curve
FFMI - Fat-free mass index
FMI - Fat mass index
SSFAUC - Skinfold thickness area under the curve

Software & Analysis Tools

FSL - FMRIB Software Library
IBM SPSS - Statistical Package for the Social Sciences
MATLAB - Matrix Laboratory
QIIME - Quantitative Insights Into Microbial Ecology
R - R statistical software
SAS - Statistical Analysis System
STATA - Statistics and Data
SUDAAN - Survey Data Analysis

Other Technical Abbreviations

CON/CTL - Control
DBA - Database
E17.5 - Embryonic day 17.5
FOV - Field of view
GA/GW - Gestational age/week
HC - High carbohydrate
HF/HFD - High-fat/High-fat diet
HP - Highly palatable
ICV - Intracranial volume
LFD - Low-fat diet
LP - Low-protein
MHFD - Maternal high-fat diet
NAC - N-Acetyl-Cysteine
TE - Echo time
TEA - Term-equivalent age
TI - Inversion time
TR - Repetition time
WM - Working Memory/White Matter
